# Determinants and relationships of climate change, climate change hazards, mental health, and well-being: a systematic review

**DOI:** 10.3389/fpsyt.2025.1601871

**Published:** 2025-08-19

**Authors:** Karolin Rückle, Mario Rohrer, Barbara Mihók, Maria Johansson, Hanna Andersson, Muhammad Saleem Pomee, Eleni Vergadi, Glykeria Rouva, Apoorv Agrawal, Balint Balázs, Erika Brattich, Maria Carelli, Claudia De Luca, Silvana Di Sabatino, Sruthi Krishnan V, Anna Molter, Francesco Pilla, Paolo Ruggieri, Anna Scolobig, Elke Hertig

**Affiliations:** ^1^ Regional Climate Change and Health, Faculty of Medicine, University of Augsburg, Augsburg, Germany; ^2^ Institute for Environmental Sciences, University of Geneva, Geneva, Switzerland; ^3^ Environmental Social Science Research (ESSRG) Group Nonprofit Ltd, Budapest, Hungary; ^4^ Institute of Ecological Economics, Faculty of Economics and Business Administration, University of Szeged, Szeged, Hungary; ^5^ Environmental Psychology, Department of Architecture and Built Environment, Lund University, Lund, Sweden; ^6^ Department of Paediatrics, Medical School, University of Crete, Heraklion, Greece; ^7^ Spatial Dynamics Lab, School of Architecture, Planning and Environmental Policy, University College Dublin, Dublin, Ireland; ^8^ Department of Physics and Astronomy “Augusto Righi”, Alma Mater Studiorum University of Bologna, Bologna, Italy; ^9^ Institutes for Comprehensive Cancer Patient Care and Research (IRCCS) Azienda Ospedaliero-Universitaria di Bologna, Bologna, Italy; ^10^ Department of Architecture, Alma Mater Studiorum University of Bologna, Bologna, Italy

**Keywords:** climate change, extreme events, mental health, well-being, systematic review

## Abstract

**Introduction:**

Impacts of climate change on human health receive increasing attention. However, the connections of climate change with well-being and mental health are still poorly understood.

**Objective:**

As part of the Horizon Europe project TRIGGER, we aim to deepen the understanding of the relationships between climate change and human mental health and well-being in Europe by focusing on environmental and socio-individual determinants.

**Methods:**

This study is a systematic literature review based on the PRISMA guidelines using Embase, Medline and Web of Science.

**Results:**

143 records were retrieved. The results show that climate change and its specific hazards (air pollution, floods, wildfires, meteorological variables, and temperature extremes) impact human well-being and mental health.

**Discussion:**

Mental health and well-being outcomes are complex, extremely individual, and can be long lasting. Determinants like the living surrounding, human’s life activities as well as socio-individual determinants alter the linkage between climate change and mental health. The same determinant can exert both a pathogenic and a salutogenic effect, depending on the outcome. Knowing the effects of the determinants is of high relevance to improve resilience. Several pathways were identified. For instance, higher level of education and female gender lead to perceiving climate change as a bigger threat but increase preparedness to climate hazards. Elderly, children and adolescents are at higher risks of mental health problems. On the other hand, social relation, cohesiveness and support from family and friends are generally protective. Green and blue spaces improve well-being and mental health. Overall, comparing the different hazard-outcome relationships is difficult due to varying definitions, measurement techniques, spatial and temporal range, scales, indicators and population samples.

**Systematic Review Registration:**

https://www.crd.york.ac.uk/PROSPERO/home, identifier CRD42023426758.

## 1 Introduction

Climate Change is one of the greatest challenges that humankind has to face in the 21st century (1). The world exceeded heat records globally in 2023 (2) and 2024 was the first year, above the 1.5°C target of the Paris Agreement (3). Projections reveal increases in climate impact drivers for the near future, combined with adverse impacts for people and the environment. The lives of people have been interrupted due to loss of income, social capacity or the destruction and loss of homes and property. Climate change and associated increases in extreme weather events have been linked with a general reduction in physical and mental health as well as overall human well-being (4).

Analysing the connection between climate change and human health is a fast-growing field of science and scientific knowledge and understanding enhances every day. For the first time in 2023 (mental) health and well-being were mentioned amongst the main topics at the Conference of the Parties (COP) 28 (2). Previous reviews, like Lawrance et al. (5), Charlson et al. (6), Middleton et al. (7), Hayes and Poland (8), Ma et al. (9), Frangione et al. (10) and Cianconi et al. (11) already highlighted aspects of the nexus between climate change and mental health globally. For example, direct and indirect pathways by which mental health can be affected (5), and the impacts of climate change on the mental health of Indigenous people (7) in various countries like Canada, Australia and the USA were reviewed.

However, to our knowledge, no review systematically assessed the environmental and socio-individual determinants in the relationships of climate change and its hazards with mental health and well-being, including a categorization into pathogenic and salutogenic. We defined environmental and socio-individual determinants in the link between climate change and human well-being that were positively associated and improved the situation of the people as salutogenic. While determinants that worsen an individual’s quality of life or circumstances were classified as pathogenic. Identifying, categorizing and characterizing these determinants is crucial for reducing exposure and vulnerability, and mitigating the mental health risks associated with climate change.

**Figure 1:**
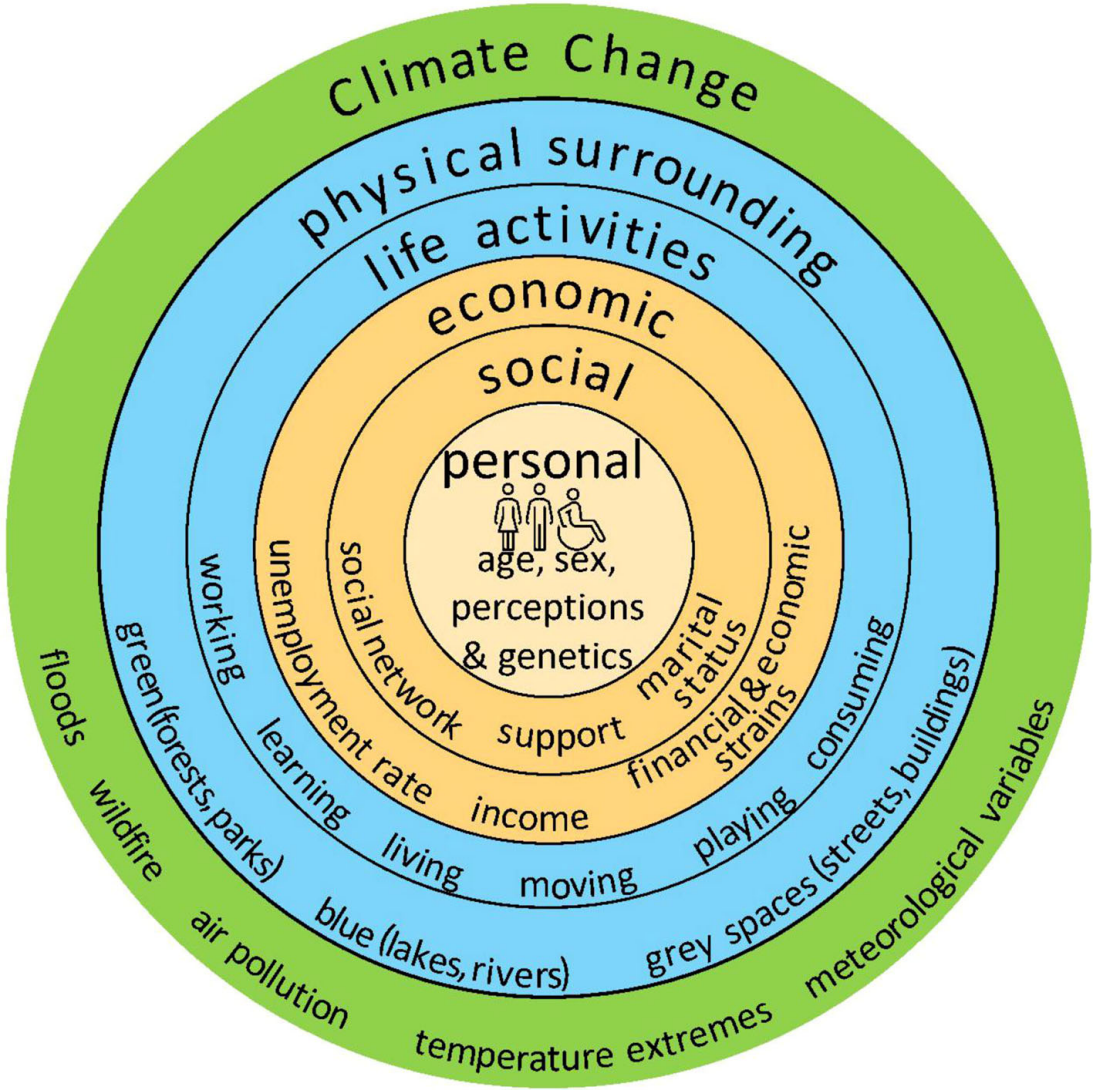
Well-being concept. Within the living surrounding people conduct their life activities (blue). These are called the environmental determinants of health and well-being and can be directly impacted by outer forces like climate change and related hazards (green). These determinants affect but also depend on economic, social and personal factors called socio-individual determinants in this study (yellow).

Many definitions and concepts of well-being attempt to define and summarise the complex correlations and layers of human health and well-being. Based on the WHO (World Health Organisation) definition, in this review “well-being is [defined as] a positive state experienced by individuals and societies. [ … ] It is a resource for daily life and is determined by social, economic and environmental conditions” (12),. We classify these conditions based on a modified version of the well-being concept from Barton and Grand called the “health map” (13). We divide the factors of (mental) health and well-being into environmental and socio-individual determinants which impact strongly on exposure, vulnerability and thus health outcomes. Note that the use of these terms is different from other fields like e.g. epidemiological exposure-outcome studies where these determinants are treated as confounders or modifiers. In this study environmental determinants represent the living surrounding (overall environment in which a human lives and conducts its daily life), like green (e.g., forests, parks), blue (e.g., lakes, sea) and grey (e.g., streets, buildings) spaces and human’s life activities (living, working, moving, consuming, learning, playing) within the living surrounding. Economic (e.g., unemployment rate), social (e.g., social network, marital status), and personal determinants (e.g., age, gender) are defined as socio-individual determinants (**Figure 1**).

This review is part of the Horizon Europe project TRIGGER [soluTions foR mItiGatinG climate-induced hEalth thReats, (14)]. It aims to deepen the current understanding of the link between climate change and health effects with a focus on Europe. The objective is to synthesise the current knowledge on the relationships of climate change and human well-being in Europe and to identify the environmental and socio-individual determinants in these relationships. The review also differentiates between pathogenic (“ill-making”) and salutogenic (“resilience-building and (mental) health-promoting”) variables (determinants) in the links between climate change and human well-being in the region of interest. The research question is:

How is human mental health and well-being in Europe related to climate change and its hazards and what role do the environmental and socio-individual determinants play?

## 2 Method

The review followed the PRISMA (Preferred Reporting Items of Systematic Reviews and Meta-analyses) guidelines (15) and was registered beforehand on PROSPERO (CRD42023426758).

### 2.1 Study characteristics: inclusion and exclusion criteria

Studies matching the criteria in **Table 1** were included. Conversely, studies dealing with non-climate-related natural hazards (earthquakes, volcanic eruption) were excluded. Additionally, topics such as justice and equality, pregnancy and neonatal issues, suicide, diet and nutrition, violence and crime were excluded, since the thematic scopes of these topics are too broad to be covered in this review or were already addressed in previously mentioned studies.

**Table 1:** Inclusion criteria.

General	- Written in English - Published between 2011 and January 2023 (time cut off due to comparison to another systematic review in the project) - Peer-reviewed
Hazards	- Addressing climate change, climate change hazards and climate induced changes like temperature extremes (heat waves and cold spells), floods, wildfires, air pollution and meteorological variables
Outcome	- Addressing outcomes of mental health and well-being, such as depression, anxiety related diseases, stress related diseases (e.g., post-traumatic stress disease (PTSD)) (further details see S1 and S3)
Population	- Human related studies for the population in Europe [TRIGGER project focus (14)], defined as the EU-member states, United Kingdom (UK), Switzerland, and Norway

### 2.2 Search strategy, data extraction and risk of bias assessment

The search strategy was based on four areas (climate change hazards, mental health outcomes, Europe, and determinants). For each area a list of terms was developed (see S1) and extended together with experts of the TRIGGER consortium in the fields of environmental psychology, climatology, sociology, economy, and medicine.

The search term list was complemented with index terms, used in the selected databases. A comprehensive filter to exclude animal studies based on the search syntax of Mierden et al. (16) was added. The search was conducted on July 16, 2023, in Embase, Medline and Web of Science. Search strategies are available in S2. For further verification of the search results, an additional search was conducted in Scopus on June 30, 2025.

Duplicates and papers published before 2011 were identified and removed via Citavi (version 6.17). Papers from 2011 to 2022 as well as January 2023 were included in the study. The preliminary and full text screening were performed in Rayyan (17). These screenings were conducted independently by two different collaborators and three additional experts were consulted to resolve conflicts. Data extraction was performed simultaneously by ten different collaborators in SRDR+ [(18) (Accessed at https://srdr.ahrq.gov/16.01.2024)].

For the risk of bias assessment (see S3), the Newcastle-Ottawa-Scale for cohorts, case-control and the adapted version for cross-sectional studies was used by two researchers in consultation with another researcher (19–21). Case-crossover studies were treated as cross-sectional studies. Additionally, the CASP [Critical Appraisal Skills Programme (22)] was used for qualitative studies.

## 3 Results

**Figure 2:**
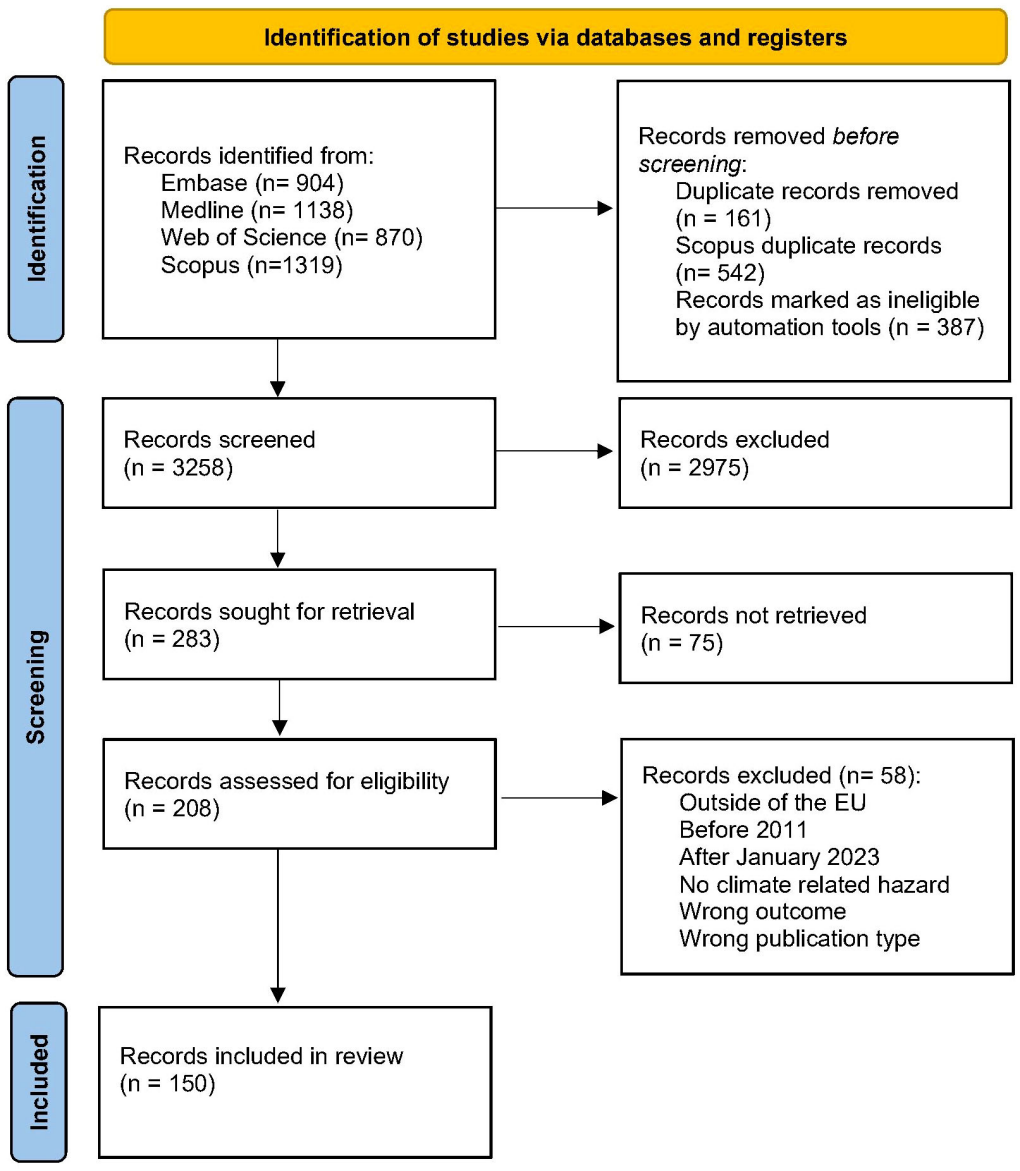
PRISMA flow diagram of the study selection.

A total of 150 papers were selected (see **Figure 2**, **Supplementary Table S6**, for characteristics). 78 studies analysed air pollution, 25 floods, 5 wildfires, 9 meteorological variables (e.g., precipitation, atmospheric pressure, wind speed) and 5 temperature extremes. Included were also climate change-related studies without specific hazards, 20 papers about climate change, investigating related perceptions and emotions and 28 which addressed the importance of the environment within the climate change-mental health-relationships.

Cross-sectional study designs were performed in 75 studies, 66 had a longitudinal approach and 9 conducted qualitative studies. The studies were mostly of moderate quality (81, medium risk), because of the used outcome measurements. The outcome scales were generally applied in a clinical context, but the results were not clinically validated and confirmed. Good quality with a low risk of bias had 56 studies, while 21 studies had a high risk. Some of the papers include cross-sectional as well as cohort analyses. Thus, the numbers in the quality analysis are higher than the actual record number.

**Figure 3:**
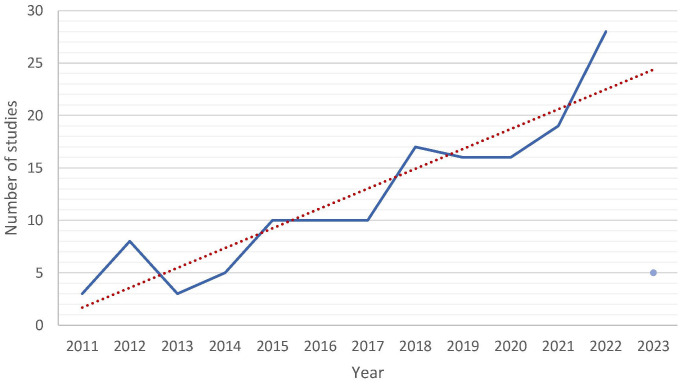
Number of studies on climate change and mental health published from 2011 until 2023. 2023 (only January) was not included in the trend analysis. The number of records of January 2023 is only illustrated as a point.

The data showed an upward trend in numbers of published records with 3 in 2011 reaching 28 records in 2022 (**Figure 3**).

**Figure 4:**
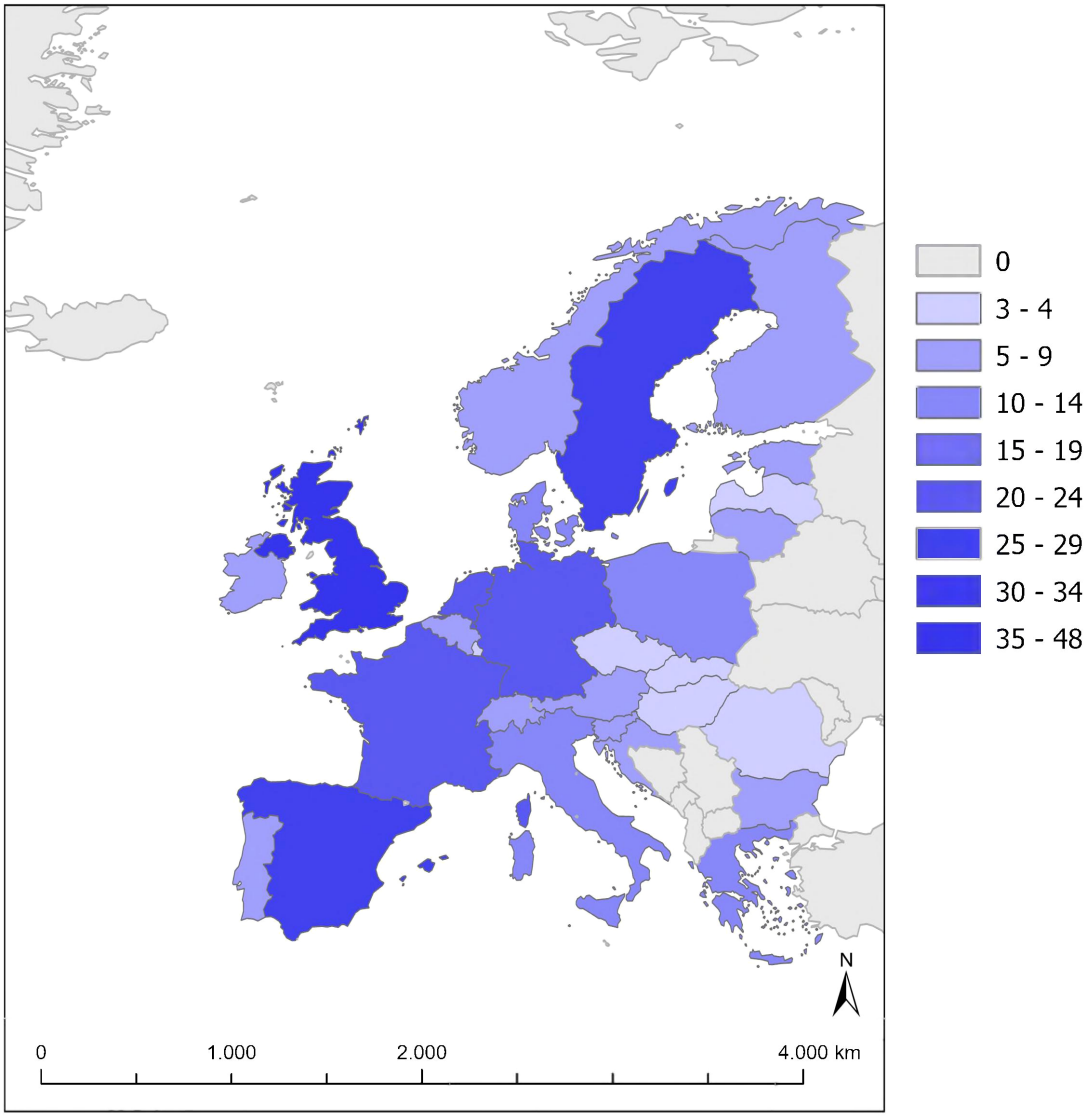
Map of Europe with colour scale representing the number of studies on climate change and human mental health and well-being.

The regional distribution is outlined in **Figure 4**. There is a high contribution from the United Kingdom (UK) (48 studies), Spain (23) and Sweden (24), while there are less studies conducted in Eastern Europe (e.g., Latvia (3), Romania, Hungary, and the Czech Republic (4 each)), other Nordic countries (e.g., Finland, Norway), Central Europe and the southern Mediterranean-Balkans.

**Figure 5:**
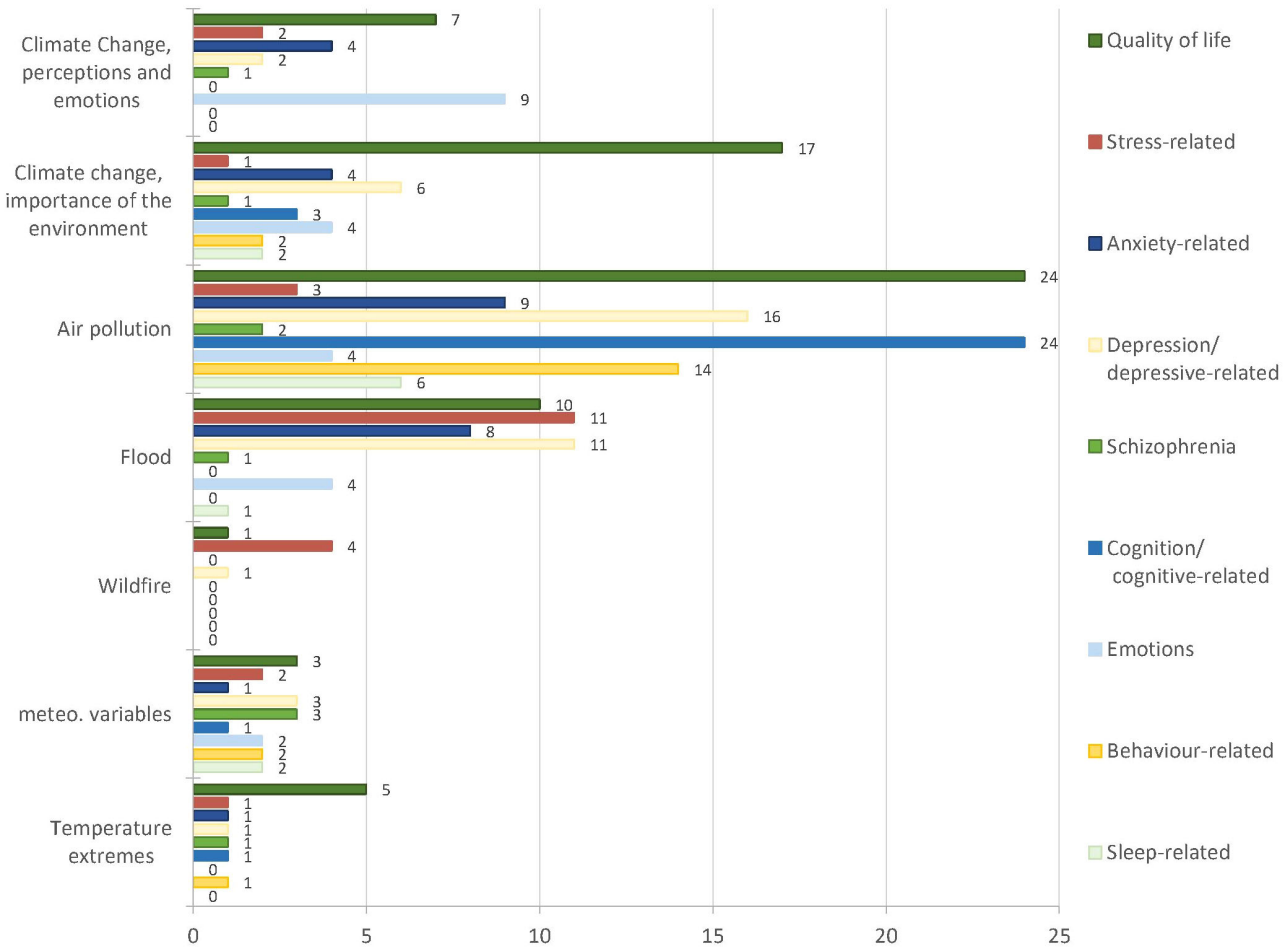
Studied outcomes per hazard.

Outcome categories (**Figure 5**) follow the WHO ICD 10 (International Classification of Diseases) codes for mental diseases, where applicable (details in S4). The most analysed exposure was air pollution. It was mainly related with overall quality of life, cognitive aspects and depression. The 3 records focusing on temperature extremes addressed almost all outcome categories, while only 3 categories (quality of life, depression and stress related diseases) were addressed in relation to wildfires.

### 3.1 Climate change, related perceptions and emotions

Climate change is a complex topic, seemingly far away from peoples’ daily reality (25). However, it can affect people’s emotions, decisions, and well-being, including climate anxiety, solastalgia or fear. This section includes 20 investigations on climate change without addressing a specific hazard like e.g., flood or wildfire.

#### 3.1.1 Outcomes

The most common outcome was the general interference of climate change with the overall quality of life and (mental) well-being, including risk perceptions and the influence of the psychological distance of climate change (24–27). This general impairment was often paired with emotions (23, 28, 29) like different levels of happiness (30), grief, worrying (31, 32), concerns, helplessness (33), solastalgia and ecological anxiety (34, 35). Other studies addressed schizophrenia and psychotic experiences (36).

Climate change appeared as a significant concern even at younger age. Thomas et al. (23) identified anger and frustration as predominant emotions, which reflects the burden felt by children facing climate-related challenges. Spence et al. (25) explored the psychological distance of climate change, revealing perceptions about climate change as both globally distant and locally relevant. Lawrance et al. (27), highlighted the distress many young people feel about climate change, suggesting a potential impact on their mental well-being and quality of life.

#### 3.1.2 Environmental determinants

**Figure 6:**
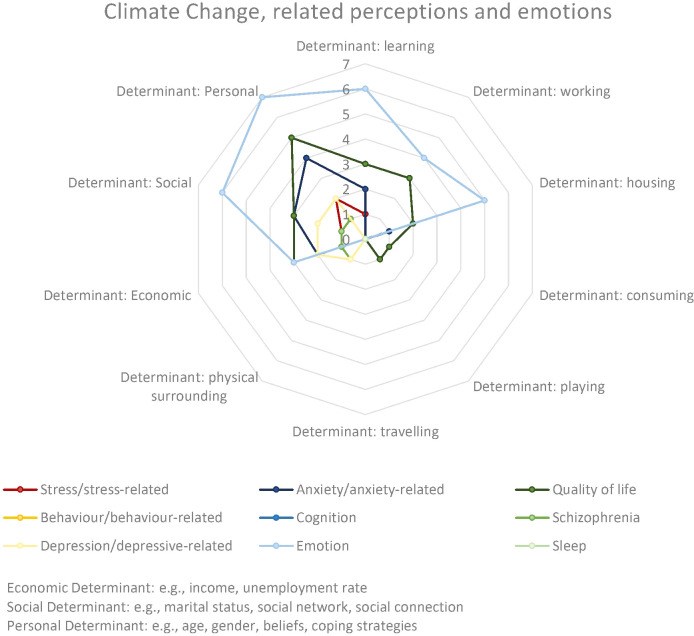
Distribution and frequency of different determinants by outcome group for climate change, related perceptions and emotions.

Mode of commuting, recreation activities and education can influence mental well-being (23, 27). Higher levels of education were negatively associated causing increased concerns about climate change (33). However, higher education enhanced perceptions of climate change among forest owners (24). **Figure 6** offers an overview on the addressed environmental and socio-individual determinants and related outcomes.

#### 3.1.3 Socio-individual determinants

The diversity of socio-individual determinants reflected a rather holistic approach of many studies. The relative socioeconomic position plays an important role in the mental health and well-being of children (25, 27). For example, Lawrance et al. (27) utilized the Family Affluence Scale-III to gauge relative socioeconomic position and assess psychological responses and mental health. Age and gender were identified to be the most relevant and pathogenic determinants especially for women and elderly. Females as well as older people tended to be more worried (32) and “1.491 times more likely to be concerned about climate change than males (33)”. For children studies considered parental characteristics such as age, education, smoking during pregnancy, and occupation. They acknowledging the potential impact of parental attributes on children’s well-being and cognitive development (23).

### 3.2 Climate change, importance of the environment

As mentioned previously physical surroundings are most directly impacted by climate change. Studies in this section (23) mostly highlighted the importance of a high-quality local environment as salutogenic factor for people’s quality of life and well-being. The papers included a broad range of qualities. They can be divided into those centering around green (and blue) spaces and those with broader perspectives, addressing diversity in the physical surroundings and considering a socio-economic context.

#### 3.2.1 Outcomes

Additionally, to the focus on human well-being and quality of life the papers addressed outcomes like different levels of happiness (37), grief and worrying (32), as well as stress (38, 39), anxiety and the morbidity of anxiety disorder and depression (40–46).

In a study of adolescents, a distinction was made between emotional symptoms, behavior (e.g., hyperactivity-inattention), conduct and peer relationship problems (47). Measurements used include health-related quality of life questionnaires like ESPRINT-15 (Cuestionario ESPañol del Calidad de Vida en RINiTis) (48), CASP-19 (The Control, Autonomy, Self-realization, Pleasure) and EQ-5D (47). Muñoz-Cano et al. (48) measured sleep with the Medical Outcomes Study Sleep Scale (MOS Sleep Scale), and anxiety and depression with the Hospital Anxiety and Depression Scale (HAD). Others were the 12-item General Health Questionnaire (GHQ-12) (40), the Mental Health Continuum Short Form (MHC-SF) (49), the 36-Item Short-Form Health Survey (SF-36) (50), SF12 Mental Wellbeing, the General Health Questionnaire score (GHQ) (51) and the Strength and Difficulties Questionnaire (SDQ-R) (47). Rathmann et al. (52) combined self-report assessments of subjective well-being with analysis of Heart Rate, Inter-Beat-Interval and Galvanic Skin Conductance, and Blood Volume Pressure. Triguero-Mas et al. (39) employed self-reports of mood, stress and perceived restoration experience with salivary cortisol tests, and cognitive tests.

#### 3.2.2 Environmental determinants

The presence, amount and types of green spaces were discussed as well as access, distance, and visibility (38–40, 43, 44, 47, 49, 53, 54). They were mostly measured by the Normalized Difference Vegetation Index (NDVI) using buffers of 100, 300 and/or 500 metres around residence (38, 40, 47, 53). A few papers also included people’s perceptions of green spaces, using an index for perceived amount of nature near home (49) or perceived restorative quality of the neighborhood (Perceived Restorativeness Scale (PRS) (40).

Cruz et al. (44) showed a mix of social and ecological determinants as relevant for the protection of mental health. Higher concentration of air pollutants, higher distance to green spaces with lakes, deprivation, higher numbers of ethnic minorities were identified as pathogenic determinants and increased the prevalence for serious mental diseases. The studies pointed to the importance for well-being but also revealed the complexity of disentangling associations between the physical surroundings and well-being outcomes. Hart et al. (37) showed that neighborhoods with higher aesthetics and more water and green were salutogenic (higher levels of happiness). Combined effects of health satisfaction, social life, ontological security, greenspace proximity, and time in nature also impact well-being positively (46). Living in residences with higher vegetated surroundings over the lifetime reduced the prevalence of experiencing premenstrual symptoms like anxiety and tension, feeling depressed and hopeless (38).

Zock et al. (43) concluded that high diversity in land use may be beneficial for physical and mental health. Presence of greenspace in the neighborhood was weakly associated with less morbidity for anxiety disorder and depression. In Dzhambrov’s (40) cross-sectional analysis the effect of residential green space on mental health was mediated by physical activity and restorative quality. While in the longitudinal analysis more green space exerted a direct effect on mental health. Triguero-Mas et al. (39) showed that perceived psychological restoration experience increased physical activity. Decreased air pollution mediated the effect of green spaces on psycho-physiological health indicators. Two papers had a narrower focus considering a specific green setting together with the activity performed, i.e., participation in community gardening (54), and forest walks (52).

Several papers took a broader perspective, i.e., land use diversity and socio-economic context. Based on multiple Dutch cohort studies (n = 32.487), Generaal et al. (42) found that urbanization level, socioeconomic, physical, and social neighborhood characteristics were associated with prevalence of depression. Distinct urban-rural inequalities were identified with remote rural regions and inner-city areas as environments with poor and more pathogenic health-related features. Cerletti et al. (50) referred to people’s perception of their proximity (in minutes) to supermarkets, local services, restaurants and cafés, public transportation services, sports facilities, parks, green spaces and quiet places. Perceived access to outdoor open space, facilities for walking and cycling plus aesthetics of the built environment were mentioned (37, 55, 56). Residential street improvements in the UK had an impact on elderlies perceptions of street walkability and safety at night, but not on the overall levels of physical activity or wellbeing (55).

The studies further evaluated the significance of physical surroundings, such as urban environmental factors and green spaces, in shaping cognitive functions (57–60). Brons et al. (47) identified a stronger influence of the school environment on the mental health of adolescents than the residential environment. Further the mode of commuting, recreation activities and education can also influence mental well-being (59).

#### 3.2.3 Socio-individual determinants

Socio-economic conditions were considered at a local scale, through neighborhood (41, 42, 47) and school socio-economic status (SES) (47). Household determinants (11 studies) included family’s socio-economic capital (53), income (41, 42, 45, 54, 56), SES (38, 46, 51), income deprivation (44) and Index of Multi Deprivation (51). Binter et al. (57) included a deprivation index comprising income level, employment rate, and educational level from each country under study. This indicator reflects the economic status of the regions and helped to assess the impact of economic disparities on children’s cognitive and motor functions. Other studies incorporated neighborhood and family SES, including average income, unemployment rate, parental education and socioeconomic vulnerability. Thereby, the studies highlighted the importance of economic background in understanding how green spaces and air pollution impact mental well-being during adolescence (58, 60).

Social conditions were considered at different socio-spatial scales, the largest scale referred to perceived neighborhood social cohesion, social network, level of trust and safety (37, 40–42, 56) and social engagement (50). The second level referred to family and household. This level included household structure/composition (46, 54), contact with family and friends and social interaction (39, 53). According to Maitre et al. (53) was “a rich social capital of the family [ … ] protective” for behavior problems of children. Family structure and family support (47), marital status, family status (45), children in the household (37) and health status of others (51) were also important. Neighborhoods with more social contacts, a stronger social cohesion and trustworthy neighbours, were linked with higher levels of happiness (37).

Among the personal determinants, age and gender were considered in almost all studies and in 6 studies together with an ethnicity indicator (40, 42–44, 51, 55). Several personal determinants were health-related variables, e.g., body mass index (BMI) (38, 54), menstrual regularity, cycle length (38), restrictions in carrying out activities of daily life (43), illness in the past year (46) and chronic diseases (55). Two studies included self-reported connectedness/relatedness to nature (49, 54).

The potential impact of parental attributes on children’s well-being and cognitive development were also acknowledged, including parental age, education and occupation (57–60). Binter et al. (57), also delved into individual demographic factors like maternal age, educational level, ethnicity, pre-pregnancy body mass index, and others.

### 3.3 Air pollution

Due to climate change weather patterns and chemical processes in the atmosphere related to air pollution are changing. The review includes therefore 78 papers which investigated the impact of air pollution on mental health and well-being. Some studies involved multiple cities (61–63). The majority (20) focused on data from the UK, with the fewest from Poland (1). The spatial location varied from local to international, with most undertaken at a local scale (32 publications). The target population of 20 studies were the working-age and the elderly [like (64, 65)], 5 targeted only the working-age population [e.g., (41, 66)], and 37 included children/adolescents [e.g., (67, 68)]. Regression modelling, e.g., logistic/linear regression was used in 57 studies, with the majority testing at 95% confidence interval [e.g., (61, 69–71)].

Articles discussed exposure to particulate matter (PM_10_ and PM_2.5_), nitrogen dioxide (NO_2_), sulphur dioxide (SO_2_) and ozone (O_3_) (72–76). The threshold levels were compared to the standards specified by WHO air quality guidelines (AQGs) (69, 77) and EU air quality standards (78). The data were estimated using different methods, including climate models (77), land use regression models (75, 76, 79), monitoring stations (80), personal wearables (66) and GIS (geographical information system) based assessments (81). The air pollution exposure was measured continuously across time (raging minutes to years) by 30 studies, intermittently and multiple times by 20 and 65 addressed long-term exposure.

#### 3.3.1 Outcomes

Studies demonstrated a strong correlation between air pollution and the outcomes, mainly cognitive, behavioral and well-being (67, 82, 83). These include impacts on cognitive development in children/adolescents and cognitive decline in the elderly (57, 59, 80, 84–86). Mortamais et al. (67) and Oudin et al. (87) demonstrated that prenatal exposure to PM_2.5_, particularly during the last trimester, may induce structural brain changes in children. This enhanced the risk for developing autism spectrum disorder (ASD) and attention deficit hyperactive disorder (ADHD). Similarly, Rivas et al. (83) showed that exposure to PM_2.5_ during early life was negatively associated with fundamental cognitive abilities at school age, such as working memory and the attentional conflict network. PM from traffic was associated with important reductions in cognitive growth and increased risk of ASD (80, 81, 84). Furthermore, exposure to air pollution can lead to neurodevelopmental deceleration, leading to cognitive dementia, such as inattentiveness, verbal memory, reasoning, and verbal influence [e.g., (88–92)]. However, some studies found no evidence for the association of air pollution with an increased risk for neurodevelopmental disorders in children (62, 93, 94). Behavioral issues were linked to air pollution, particularly in children (6 papers). Studies reported a higher incidence of ASD, ADHD, behavioral development and other behavioral problems (53, 62, 93–96). Bloemsma et al. (58) revealed that higher exposure to ambient air pollution was related to poorer mental well-being in adolescents. However, this association attenuated after adjustment for green space. Conversely, Jorcano et al. (97) and Midouhas et al. (98), presented conflicting evidence, suggesting no significant association between air pollution exposure and emotional or behavioral problems in children.

**Figure 7:**
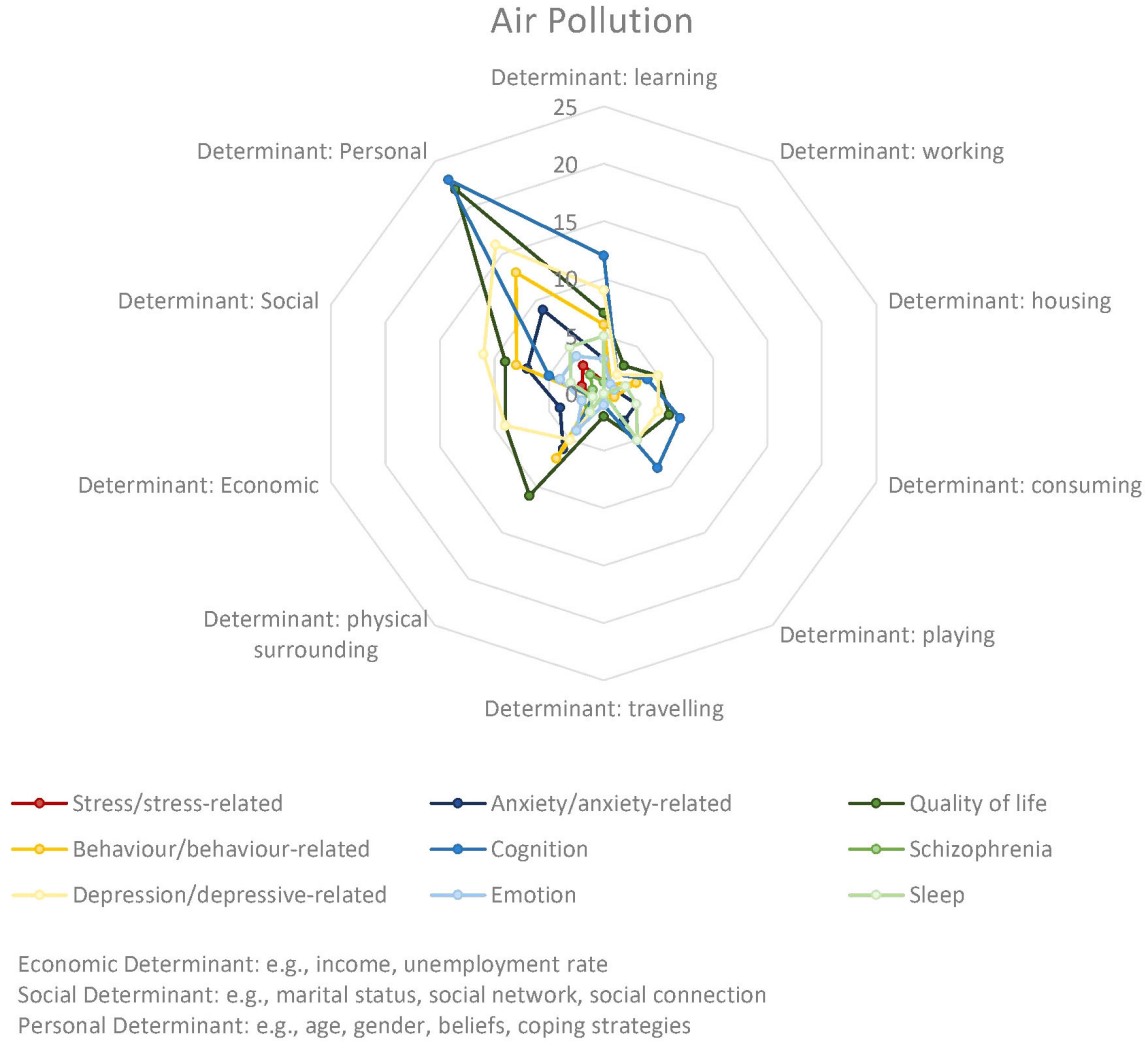
Distribution and frequency of environmental and socio-individual determinants by outcome group for air pollution.

Rittner et al. (72), supported that reduction of PM_2.5_ emissions would have a substantial impact on mortality and adverse health effects, improving overall quality of life. Several studies revealed a concerning link between air pollution and adverse psychological outcomes in children (68, 70, 77, 99, 100). These studies observed a correlation between childhood exposure to ambient air pollution and an increased risk of depression onset, psychopathology, and overall psychiatric illness in late adolescence. Additionally, Newbury et al. (36), demonstrated that socioenvironmental risk for schizophrenia during upbringing is correlated with a genetic risk (e.g. family psychiatric history). This correlation can be seen in **Figure 7** where most outcomes are related to personal determinants.

Further, air pollution was closely associated with depression in 10 studies [e.g., (42, 45, 65, 77)], anxiety in 9 studies [e.g., (43, 70, 97, 101)], and stress in 2 studies (102, 103). Additionally, 6 studies found a correlation with sleep quality (53, 70, 104–107), insomnia (104) and sleep duration (106, 107), leading to reduced life satisfaction. Long-term effects of air pollution on well-being were significant, with chronic exposure leading to sustained mental health issues. This includes chronic depression and anxiety, highlighted by Orru et al. (108) and Ronaldson et al. (109).

Allergens and allergies (4 paper), can be a severe personal predisposition for a person’s quality of life, causing limited quality of life (physical activity) with differences between perennial and seasonal allergies (48, 110–113). Additionally, specific mental health aspects (47) such as stress (114), anxiety (48, 114), sleep quality disturbances (48, 113, 115), depression (48) and overall satisfaction with life were identified. The reported confounders in studies were inconsistent. Highlighted were sleep quality and duration with quality of life and cognitive outcomes (40, 82, 115), depression connected to cognition and schizophrenia (36, 79, 86) and satisfaction with life (quality of life) (46).

The data sources varied, as many (41 studies) relied on self-reported questionnaires [e.g., (45–47, 58, 65)]. This included general health questionnaires, behavioural and cognitive tests, depression and anxiety scales, sleep quality surveys, and other health-related questionnaires. Six studies used clinical tests and screenings to assess cognitive functions, mental health, and other health-related outcomes (57, 59, 85, 107) as well as the SF-12 Health Survey and the 31-item Rhinosinusitis Outcome Measure (RSOM-31) (110), the Paediatric Asthma Quality of Life Questionnaire (PAQLQ), Paediatric Asthma Caregiver’s Quality of life Questionnaire (PACQLQ) (111). Others utilised data from national surveys and statistic databases (29 studies) such as UK Biobank, Lithuanian Health Information Centre databases, Betula [e.g., (74, 79, 89, 109)]. Verheyen et al. (103) measured hair cortisol concentrations to assess stress levels and their correlation with environmental determinants.

#### 3.3.2 Environmental determinants

Various studies revealed a multifaceted set of determinants. Studies included green space [e.g., (44, 47, 59, 82)], noise level (41, 45, 64, 70, 109), traffic noise annoyance (40, 95), traffic density (62), urbanization grade (42), and proximity to major roads (103). These variables were significant in determining the level of noise pollution, air quality, and the amount of green space. Dadvand et al. (59), Hiscock et al. (46) and Orru et al. (116) mentioned that the primary mode of commuting contributes, too.

Studies highlighted that education is linked to SES and influences lifestyle choices, which influence mental well-being (104). Years of education (42, 45, 64, 74, 82, 105) and educational qualification (115, 117) played a role in determining mental health outcomes. Smoking, alcohol intake, and proximity to fast-food outlets (101) was additionally related to lifestyle choices.

The living environment, including indoor air quality and housing conditions, was crucial to mental health. Studies indicated that living areas (urban/rural) (62, 65, 71, 86, 117), house value (42), household size (53, 61) and home satisfaction (46) were also significant environmental determinants.

Determinants such as physical activity [e.g., (40, 76, 79, 82, 104, 109, 118)], time spent outside (46, 85), and engagement in recreational activities (101) were salutogenic as they are related to stress relief, mood enhancement, and overall well-being. The amount and frequence of the addressed determinants can be seen in S5.

#### 3.3.3 Socio-individual determinants

Numerous studies (16 studies) underscored the pivotal role of household income as an economic determinant [e.g., (42, 61, 119–121)]. Generaal et al. (41) indicated that lower-income individuals might face more severe adverse effects due to constraints in accessing cleaner living environments or protective resources. Interestingly, Oudin et al. (91) discovered an increased susceptibility to asthma linked to air pollution among children from higher-income families. Additionally, Abed Al Ahad et al. (119) and Shiue (71) highlighted financial situations as crucial, significantly influencing the impact of air pollution.

Critical social determinants identified by 16 studies in the context of air pollution’s impact on well-being included marital status (45, 59, 83, 84, 116) family-related factors such as household composition (46), family support (47), parental education (67, 83, 95, 98), siblings at birth (95), and ethnicity (40, 122). Likewise, SES and community-related factors like social cohesion and safety (41, 43), civil status (105), social benefits (41, 82) and social isolation (79) were highlighted. Shiue (71) further investigated the influence of social participation, the quality of social services, and the social exclusion index on the effects of air pollution on adult mental health. Collectively, these studies underlined the multifaceted nature of social determinants in mediating the mental health consequences of air pollution.

Personal determinants, like demographic attributes, including age, sex, and ethnicity, were identified as key influencers of individual responses to air pollution (44, 47, 58, 88, 122). Furthermore, low birth weight, health conditions, particularly chronic diseases and asthma, significantly modified the impact [e.g., (66, 86, 95, 104, 123)]. Individuals with pre-existing health issues were found to be more adversely affected (74, 108, 124). Lifestyle choices, such as alcohol consumption, smoking habits (65, 88, 107), and BMI (75, 76, 101, 103, 109, 118), also played a crucial role.

Genetic factors, notably the presence of the APOEϵ4 allele, is a well-defined risk factor for Alzheimer’s disease. In children with the APOEϵ4 allele, exposure to traffic-related pollution was linked to higher behavioral problem scores, less cognitive improvement, and smaller brain volumes (88). Adults exposed to air pollution showed reductions in grey matter volume and increases in white matter volume in areas associated with Alzheimer’s progression (64). Long-term exposure to PM_2.5_ was also linked to an increased risk of dementia, regardless of APOE genotype (90). Additionally, a study involving the UK Biobank (74) found that a combination of air pollutants and the APOEε4 alleles increased the risk of dementia. Ethnicity did not significantly impact dementia risk factors, despite differences in APOE allele frequencies (79). Lastly, the impact of air pollution on cognitive function was partly mediated by lung function, with non-APOEε4 carriers showing a slightly higher mediation (86).

Other notable socio-individual determinants included neighbourhood-level, family SES [e.g., (58, 70, 77, 83, 85, 86, 100)], school/home/urban vulnerability index (41, 79, 90), and area-level deprivation index (51, 57, 65, 67, 103, 123), highlighting the importance of the broader socio-economic environment. Besides, Gao et al. (117) and Ma et al. (74) considered the Townsend deprivation index as significant contributor.

### 3.4 Floods

24 papers analyzed the effect of floods as extreme events to well-being and mental health. Approximately two-thirds of the research (14) were conducted in the UK. Others were from Germany (3), the Netherlands (2), Ireland, Croatia and France (1-1-1). 2 studies were international (multi-country).

In 7 papers children/adolescents were the affected target group, while others included adults and the general population. Only 5 studies covered smaller (< 100 people) cohorts using qualitative methods, while the rest reported larger population sizes (between 447 and 20.982).The largest sample comprised more than half a million habitants.

Most of the research focused on specific flood events, like the aftermaths of the 2013/2014 floods happening in many regions of Europe and in the UK (12 paper, [e.g., (125–129)]). Lorenzoni et al. (130) and Grimm et al. (131) included other disastrous events besides flood in multi-case, multi-country studies. Impact of flood events from excessive rainfall (132, 133) or coastal storms (134, 135) were also subject of investigations. The flood events resulted in displacements in some cases [e.g., Mort et al. (127), Munro et al. (136)].

Few studies (33, 137, 138) explored the general perspectives of people on risk of flooding, possible community actions and preparedness in regions potentially affected. Cruz et al. (44) included the distance from the flood zone as a variable of mental illness prevalence.

#### 3.4.1 Outcomes

The primary outcomes from exposure to floods were the deterioration of quality of life, mental health in general for people affected (127, 129, 130, 134, 139–141), and the higher prevalence of stress-related symptoms, PTSD, anxiety and depression [e.g., (126, 136, 142–144)]. Lamond et al. (143) suggested, that “households experiencing one symptom frequently are more likely to experience other and multiple psychological impacts on a regular basis”, which refers to a high-level co-morbidity.

In studies exploring the impact of particular flood events, quality of life included various aspects (e.g. mental health, resilience, or well-being) and was assessed via different scales, such as the Satisfaction with Life Scale (125), the health-related EQ-5D-5L (145), or via questionnaires/surveys (138, 140, 141). Qualitative interviews (130, 139), art-based and participatory inquiries were used to explore experiences in-depth (127).

Stress, anxiety, PTSD, depression were investigated via questionnaires/surveys using different scales, e.g. Impact of Event Scale-Revised (IES-R) (131), PTSD Checklist civilian version (PCL-C) [e.g., (132, 136, 142)], Generalized Anxiety Disorder Scale (GAD-2) (136, 142, 144, 146) and the Patient Health Questionnaire (PHQ-2) for depression (e.g. 136, 146).

Most of the studies investigated the impacts of the flood after the event (few months or years retrospective). Motreff et al. (134) analyzed the acute effect of a storm event in France via psychotropic drug deliveries. Three weeks after the flood event, drug deliveries showed an increase. Bunyan et al. (33) suggested that a lack of understanding about the interplay between climate change and flooding contributed to concerns among residents, including children and adolescents. The temporal pattern of psychological distress was an essential aspect of understanding the impacts: Hieronimi et al. (139) noted “interviewees mentioned two points of time of greatest psychological stress: the time of the flooding, and the time when the physical exhaustion decreased, and the processing [ … ] began”.

Jermacane et al. (142), Bakic and Ajdukovic (125), and several others showed that negative effects of flooding can persist for years after the actual event, leading to increased risk of psychological morbidity, PTSD, depression, and anxiety among affected individuals. Mental health issues can continue for at least 3 years post-flooding [significantly for depression and PTSD (146)], with a decrease in prevalence over time. Respondents also reported experiencing problems with performing usual activities following flooding (145). Even six years after the flooding, “some young people in Simbach am Inn still struggle with recurring anxiety during heavy rain” (139). Displacement due to flooding also had a significant impact on mental health, with displaced individuals experiencing worse outcomes compared to those who remained at home (136). Especially children/adolescents who may struggle with detachment from familiar places and resources (material and social) suffer more (127). Even those not directly affected by flooding can experience increased psychological morbidity (144). O’Neill et al. (138) demonstrated that flood risk perception has more impact on the emotional component of floods than the actual location.

#### 3.4.2 Environmental determinants

The three most frequently addressed determinants were education/learning (14 papers), housing/living (15 papers) and working (12 papers) (compare S5).

Results showed that level of education might have no or little influence on the outcome (e.g. 131, 136). Waite et al. (144) highlighted the disruption to education caused the increase of psychological morbidity in the disrupted population. Bakic and Ajdukovic (125) found that higher education was related to fewer symptoms of PTSD after the flooding. According to Bunyan et al. (33), respondents with lower levels of education were less likely to be concerned about flooding and climate change.

Since flooding has a profound adverse impact on people’s homes, housing and changes in living conditions were highly important determinants and tend to be rather pathogenic. Disaster Property Loss was scored in Wind et al. (132) as an indicator of the severity of the individual disaster experience concerning the damage and loss of possessions. Experiencing storm- or flood-related exposure and damage in the home increased the risk of having common mental disorder (126). This was in line with findings of Jermacane et al. (142) and Mulchandani et al. (146), showing that those experiencing persistent damage to their homes had greater odds of psychological morbidity. Determinants associated with increased odds included the depth of floodwater, loss of utility, or more than 24h of flooding of the home (144).

Walker-Springett et al. (129) indicated that flood events can alter residents’ sense of place as the once private home becomes less secure and no longer a place of refuge after being invaded by water. After repeated floods in Germany, Kuhlicke et al. (140) found that with each flood event experienced, households developed certain flood-related capacities. These helped them to reduce damage and restore buildings faster than after the previous flood. In their study, Bunya et al. (33) found that residents of Portsmouth living outside the city centre were less likely to perceive helplessness in tackling climate change and flooding, both individually and through local collective action. Poorer people were more prone to flood damage due to location of their homes (higher risk areas) and lack of ownership or insurance. They became more vulnerable to permanent dislocation or lack of resources for refurbishment (127). Employment status did not play a significant role in the outcome, rather it seemed to have an indirect deteriorating effect, in terms of financial difficulties (126).

#### 3.4.3 Socio-individual determinants

Social determinants such as community and interpersonal resources, social support and perceived social support (125, 132, 133),, community resilience (130), dislocation, family separation (127, 139), social capital, collective efficacy (132, 133), social resilience, network and community, community support and community relation (129) were used. Personal determinants included were e.g., age, gender, individual resources, war veteran status, ethnical background, general health, previous illnesses, trust and personal resilience. Economic determinants covered mostly the household income and the level of deprivation (126).

Bunyan et al. (33) identified gender to play a multifaceted role in increasing concern and perception of risk about flooding and climate change for females. At the same time females were less likely to perceive themselves and the city council to be helpless. This is in line with Graham et al. (126) findings. Other rather pathogenic determinants besides being female were aged 16–24, living in a deprived neighbourhood, being in debt arrears and having poorer general health. Furthermore, females were at higher risk for peritraumatic and posttraumatic stress but had lower risk perception (131). Motreff et al. (134) similarly found women to be at higher risk for mental disorders after the storm event.

As Bakic and Ajdukovic (125) found, individual resources, community economic development, and trust in community leadership played an important role in improving community mental health after a disaster. Individual resilience and interpersonal resources (support from family, friends, and partners) had direct salutogenic effects on mental health and life satisfaction. Social capital, relational networks, perceptions of agency, self-efficacy and capacities for sense-making positively influenced post-flooding well-being. Existing social capital was important, nevertheless, flood events can result in new networks to form (129). Children and adolescents’ mental well-being was specifically dependent on the social support and their parents’ well-being, preparedness and coping mechanisms (127, 139).

Trust towards local community was also more helpful to recreate life after the event when there was less trust towards professional organized support, or it was not accessible (130). On the other side, fracturing community networks, separation due to displacements, loss of access to social services and capital showed to be pathogenic and evidently increased the prevalence of mental disorders [e.g., (132, 133)]. Interestingly, Wind et al. (133) found that social capital and the feelings of cohesiveness may protect against depression (salutogenic). However, participation in social structures may increase the prevalence of anxiety disorders. In addition, more social support was related to higher prevalence of depression.

Mental health deterioration was negatively correlated to household income (lower income levels) (143). Other determinants were the amount of timely (at least 12 h) warning before the flood events and displacement as being a protective salutogenic determinant against mental disorders (136). Concerns about health, problems with relationships and loss of sentimental items were associated with poor psychological outcomes (128).

### 3.5 Wildfires

In the selected sample 5 papers (147–151) focused on wildfires, despite the severe consequences on health and well-being of these events in the short, medium, and long term. The papers primarily investigated PTSD after exposure to wildfires in adolescent/children or firefighters (148–151). Two studies were conducted respectively in Greece and Portugal. One study was a multinational study (Sweden, Italy, Spain, Poland, France) on the risk perception of wildfire (147).

Past research showed that high percentages [ranging from 9% to 29% (150)] of those experiencing a wildfire suffered from PTSD that impacted their lives and well-being in the long term regardless of age. Exposure was measured primarily as experience of a wildfire, but no thresholds were used to measure wildfire intensity.

#### 3.5.1 Outcomes

The main outcomes addressed were PTSD, anxiety or depression after exposure to wildfires, primarily found through questionnaires (self-reported) or interviews. Examples of standardized tools used for these studies include: the Children’s Revised Impact of Event Scale (CRIES-13) (148); 25-item Strengths and Difficulties Questionnaire (SDQ), Quality of life test: Kidscreen-10 (150); or clinical psychiatric interviews protocols for anxiety disorders (151). The research was conducted maximum 6 months after wildfire exposure. Fonseca et al. (149) explored the psychometric properties of the Portuguese version of the Child Post-traumatic Cognitions Inventory, revealing that children and adolescents, exposed to a wildfire disaster, exhibited significantly higher scores on stress-related cognitions. Pereira et al. (150) further investigated the dimensionality and measurement invariance of the CRIES-13 in a sample of Portuguese children and adolescents. They found a link between wildfire events and increased stress, particularly PTSD symptoms. The Greek study by Papadatou et al. (148) highlighted adolescents, experiencing elevated levels of stress and depression. Pre-disaster life events and post-disaster losses contributed to higher symptoms of both PTSD and depression.

#### 3.5.2 Environmental determinants

The most common determinants (see S5) included relocation, household damages, or household close to the flames. However, Papadatou et al. (148) stated that it was not the presence in the area or the proximity to the wildfire itself, which was associated with the prevalence of PTSD, it was the subjective threat of one’s own or significant others life and property. Other determinants were education and occupation (148–151). For firefighters seasonal employment was also a determinant (151).

#### 3.5.3 Socio-individual determinants

Most studies (147–150) identified age and gender as determinants in the connection to wildfires. Theleritis and colleagues (151) found that coping strategies like risk minimisation or blame (e.g., blaming others or the government for the events) were associated with greater likelihood of PTSD in firefighters. Other determinants included perceived social support, which simultaneously was associated with a higher prevalence of PTSD symptoms (greater perceived social support), decrease in depression symptoms (lower perceived social support) and previous experience with wildfires (148). Children seemed to have higher stress and poorer life quality if they perceive themselves as fragile, which could cause maladaptation and lower prosocial behaviour (149).

### 3.6 Temperature extremes and other meteorological exposures

11 papers investigated the relationship between exposure to meteorological variables and adverse mental outcomes, while only 5 analysed the impact of temperature extremes. The studies on meteorological variables were conducted in France, Ireland, Germany, Switzerland (2 each), United Kingdom (1) and 2 multinational. Additionally, studies on temperature extremes were mostly (2 of 5) multinational studies. In general, the studies vary in exposure definition and source of exposure data. For temperature extremes, 4 studies (152–155) utilized station observations, while one (147) utilized a questionnaire administered once. Also, the two studies based on station data differed in terms of exposure duration: findings of Arbuthnott et al. (152) were based on long-term temperature (1996-2011), Pogacar et al. (153) utilized only one year temperature records. Messeri et al. (154) created a microclimatic monitoring by installing a weather station at the study location. The definition of thresholds also differed. The papers are based on climate indices, one using average daily maximum and minimum temperatures (152), heat wave, measured with mean temperature, maximum temperature, hottest days (155) and the approximately 97^th^ (1986-2015)/96^th^ (2006-2015) percentiles of average temperatures (153). Messeri et al. (154) used the biometeorological indices Universal Thermal Climate Index (UTCI) and Wet Bulb Globe Temperature (WBGT). Considering other meteorological variables, a wide variability of data source, exposure duration, measurement frequency and exposure definition was found. Most studies (9) used station observations, and 2 studies data from personal wearable sensors. 4 studies investigated continuous long-term exposure data, considering different lengths in data records (from 12 months up to 30 years). Short term exposures, though continuous, differed widely in measurement frequency. 3 studies defined the exposure using thresholds from percentiles of the data distribution, 6 studies used climate indices (in 1 case multiple indices were employed), while the remaining studies used the Foehn index and wet and dry spell definitions.

#### 3.6.1 Outcomes

Studies highlighted outcomes such as: reduced quality of life/well-being, years of life loss, mortality (152, 156), increase in heat stress (153–155), psychosis (157), depression and mania (158), cognition problems like dementia (157) and emotional problems (159). The outcome data source varied from national survey, statistical data (4 studies), hospital records (3 studies), and self-reported questionnaires (3 studies). Most studies (8) did not investigate biological mechanisms. Three studies discussed that uncomfortable weather conditions during summer can trigger psychiatric hospitalizations (158). Others reported linkages between serotonin production and exposure to bright sunlight and the role of sleep as mediator between some meteorological factors and psychiatric admissions (160). Specifically, this study associated more continuous sleep patterns with high barometric pressure, less precipitation, and lower temperatures. Exposure to temperature extremes was positively associated with a reduced life expectancy (152) or likewise in daily years of life disparities (156) and relative risk of death (157). High temperatures were also associated with an increasing risk of mental health hospitalizations (160, 161). The association of adverse outcomes with other meteorological stressors was less significant, inconclusive or inconsistent (160), although an association between barometric pressure and mania was found (158). Further Mikutta et al. (124) suggested that external meteorological stressors, such as foehn winds, could alter the mental status of the most vulnerable populations.

#### 3.6.2 Environmental determinants

10 of 16 papers addressed determinants but only 4 discussed their role (see S5). Interestingly, the reported determinants varied across the studies. Specifically, McWilliams et al. (160) analysed the effects of determinants related with the working conditions and work dynamics, while Pogacar et al. (153) analyzed the number of hours spent working outdoor and educational level. Page et al. (157) analyzed the effects of living conditions, particularly the residential region. Finally, Peisker (159) analyzed physical surroundings, educational level, employment condition and presence of children in households. Results suggested that alcohol consumption increases the possibility to suffer from mental diseases, and the level of education and working-class occupation is positively associated with higher environmental concerns. The number of hours spent working outdoor did not seem as relevant. More important for the negative outcome was the potential and degree of outdoor workers acclimatization.

#### 3.6.3 Socio-individual determinants

The most common personal determinants were sex/gender and age (10), one study included the presence of chronic diseases (153). Social determinants were family dynamics (160), share of age under 35, urban population and urban community (159). Peisker (159) considered also economic determinants and found that gross domestic product, energy prices, consumer price inflation, unemployment rate, industrial greenhouse gas intensity and income inequality were identified as drivers of concern. Benmarhnia et al. (156) analyzed the effects of neighborhood SES. Age was positively correlated with a higher risk of adverse outcomes from temperature extremes, but also with reduced environmental concerns. One study (157) also suggested that people with mental illness represent a vulnerable group during heat, particularly younger patients with alcohol and substance misuse living in specific environmental settings. Furthermore, previous medication intake, impacting the physiological homeostasis can be related to higher mortality and morbidity in the context of temperature extremes (161). Environmental concerns rise also in correspondence with working class, SES and educational level, and type of environmental surroundings, which significantly increased the vulnerability to extreme temperatures. Messeri et al. (154) analyzed the ethnic differences in heat stress perception and received information about heat protection by native and migrant workers in Italy.

## 4 Discussion

The papers included in this review focused on the effects of climate change on perceptions and emotions as well as on the importance of the environment. With respect to specific hazards, most studies analysed air pollution and floods, while temperature extremes and fluctuations in meteorological variables were largely neglected. The review also shows that wildfires and their outcomes were not addressed frequently. Nevertheless, it was evident that all hazards were associated with a broad spectrum of mental health and well-being outcomes.

There is no consistent methodology to define the hazards, due to their different nature. Air pollution represents a continuous environmental burden (with peak episodes and spatial variations), while floods, wildfires, and heat waves are singular extreme events. However, there are also large differences in the definition of the same hazard, i.e., different measurement techniques (e.g., station data, wearables), indicators (e.g., continuous time series, percentile-based thresholds), periods (singular events, short and long time series), and spatial range of influence. Furthermore, some hazards such as rare, singular extreme events, and observational data are still scarce. While none of the hazards in the studies was directly attributed to climate change, these hazards are increasing and intensifying rapidly under ongoing climate change, thus more studies with standardized exposure definitions are of high importance. The scope of climate change and its relevance for perceptions and emotions as well as the importance of the environment represent special cases in this review since a precise hazard definition is even more difficult. Relevant to mention in this context is eco- or climate anxiety. Not yet defined as an actual mental illness, eco-anxiety can affect anyone regardless of a direct or indirect climate change impact or how resilient one is. The perception of being threatened can be enough. Thus, the storytelling, reporting and communication around climate change in science, media and by politicians is important. To a certain decree anxiety is necessary to become active and show pro-adaptive behavior, yet when it becomes too much, it can turn into maladaptation combined with anger, grief, fear and cause “freeze” responses. Scientists still debate whether eco-anxiety tends to encourage adaptation or results in maladaptation (162–164).

Different concepts and definitions on mental health and well-being, plus differences in the targeted outcomes, outcome definitions, measurements, scales, and study population limit the comparability between the studies. The data set is characterised by elevated heterogeneity (qualitative interview and quantitative data); consequently, quantitative or meta-analytic procedures as well as a GRADE analysis [Grading of Recommendations, Assessment, Development and Evaluation (165)] were not applicable within the scope of this review. The scales to measure the outcomes were also crucial for the risk of bias assessment. Since their results were not clinically validated, only probability statements could be made. Furthermore, outcomes can vary highly, from short-term acute events to long-term effects and are embedded in an individual’s personal setting. The general personal well-being as well as the well-being related to each life activity fluctuates over time (166). In the context of extreme events such as floods, authors [e.g., (139, 145, 146)] stated that years later anxiety, depression and PTSD can still be detected. This suggests that long-term monitoring and analysis can generate further information on well-being determinants.

Mental health and well-being are highly individual and complex aspects of a person’s life as well as the environmental and socio-individual determinants that each life is embedded. Therefore, the determinants which govern exposure, vulnerability and finally health risk from climate change are not always clear and are difficult to be generalized. Many studies included determinants as covariates without stating their effect size in the dynamic. Likewise, classifying the determinants into salutogenic and pathogenic variables tends to be complex. However, based on the collected data, it is possible to highlight some common potential pathways and indications towards pathogenic or salutogenic, see **Table 2**.

**Table 2:** Relationships of climate change (hazards) and mental health and well-being outcomes.

Environmental Determinants	1. The learning environment (education) plays a role with respect to exposure and vulnerability. Persons with higher education tend to evaluate climate change as a higher risk and concern, yet education supports better preparedness and less helplessness in tackling climate change especially in females (23, 24, 33, 144, 147, 153). 2. Green and blue spaces can enhance mental health, while grey spaces and higher urbanicity tend to have pathogenic impacts (38, 39, 42–44, 55). Around 42 studies addressed the mediation potential or impact of the physical surrounding on mental health and well-being, which makes the physical surrounding the most frequently addressed determinant.
Socio-individual determinants	3. Age and gender are relevant determinants for all exposures. In this regard, females are at higher risk than males (32, 33, 126, 131, 134, 156). Females do worry more and have higher concerns about climate change than men (32, 33) and generally have a higher risk perception than men of the same age (147). Furthermore, they are of higher risk of mental diseases as well as peri- and posttraumatic stress in the context of floods (131). 4. Social determinants are in close relation to personal determinants (e.g., gender, age, coping strategies, how vulnerable one views oneself) and are contributing factors for depression and stress-related disorders. Factors like social support, network, relation, cohesiveness and support from family and friends are in general protective variables (37, 47, 125, 129, 143, 148). 5. Financial strains and lower income are risk factors for mental health after floods and act as determinants in the relationship of air pollution and quality of life linked through often higher pollution in the residential area (71, 119, 126, 127).

Socio-individual determinants play a central role for a person’s well-being and can be salutogenic or pathogenic, promoting resilience or intensifying vulnerability. Some determinants which act to protect and strengthen the resilience for one mental health outcome can increase the vulnerability to another mental health outcome. Thus, in the context of floods and wildfires, social determinants like perceived social support, social relation, social capital, and the feelings of cohesiveness can be salutogenic and resilience factors for well-being and mental health (i.e., depression), but can be pathogenic factors for the prevalence and existence of anxiety disorders. Determinants related to life activities, in particular consuming, playing (recreation) and moving were rarely researched. This might be caused by 1. limitations of the review. Consuming could be addressed more often in combination with diet and nutrition, which was excluded. 2. minor correlation with the exposure or 3. insufficient consideration within the presented studies. The life activities learning and working were often combined with social and economic determinants such as family, neighbourhood, or individual SES. A comparison of these combined determinants is only possible to a limited extent, as no standardised definitions exist, and different variables were used for the calculation. For example, Benmarhnia et al. (156) used the neighbourhood level of education as SES, while Brons et al. (47) applied unemployment rate, standardized median household income, and share of households with a standardized income below the poverty line.

The regional distribution and number of the studies does not necessarily match with the frequency of hazards. For instance, the high number of studies on floods does not mean that the UK is suffering from an unusual number of floods compared to other EU countries. The most analysed events where the floods in 2013/2014 and 2015/2016 as representative for previous events [like 2012 and 2000/2001 (167, 168)]. Their investigation is of high relevance due to ongoing climate change.

To ensure the participation of all members of the research team in the review process, a common denominator was employed regarding the language of the papers (English). This decision to limit the selection to studies published in English entails the risk of overlooking relevant locally published studies, especially in countries with low numbers of records. Those studies could provide a better understanding and view of the local context and hazard frequency. Nevertheless, it would have required always a minimum of two native or fluent speakers to address one article (four eyes principle). Further, the use of back and forward citation search or expert consultation as well as more databases, e.g., PsycInfo, could also have broadened the scope and reduced the risk of overlooking relevant published studies.

Several strengths of this review can also be highlighted. Climate change is a global phenomenon, yet the impacts are experienced locally. Resources to cope, mitigate and adapt to the consequences differ regionally. Thus, the regional European focus offers a more detailed analysis of the connections and the salutogenic and pathogenic determinants in a Western setting, since the challenges are different compared to other regions in the world. Dividing the determinants into salutogenic and pathogenic is a further strength since it has not been done before and provides a clearer distinction of each determinant. This helps to design tailored mitigation and adaptation strategies. The socio-environmental system approach of this review presents a holistic perspective of human life’s including all facets of it. This was further strengthened by developing the search terms and strategy with a large interdisciplinary team.

## 5 Conclusion

This review is the first to comprehensively collect and identify the relationships and determinants between climate change, its associated hazards and human mental health and well-being outcomes in Europe. It is evident that there is a direct correlation between climate change, mental health and well-being. This correlation is characterized by impacts on the overall quality of life and mental well-being, grief, worry, solastalgia and ecological anxiety, as well as stress. These findings point out the need to address and act on the negative impacts via adequate information of the populations, including empowerment and “positive storytelling”. Also psychological guidance and adequate coping strategies in addition to strengthen community cohesion and resources are required. Thus, effective communication, prevention and intervention strategies need to be designed to understand and address needs of different groups, and types of experienced psychological impairment adequately.

The results underline that mental health and well-being were influenced by an extremely complex interplay of different determinants, varying on population group to an individual’s scale. Indirect linkages of climate change and mental health were moderated by climate change related environmental hazards, such as air pollution, floods, wildfires, and heat waves. The outcomes vary with respect to the considered hazard. For a better comparison of the different exposure outcome connections standardized methods or frameworks should be developed for each hazard.

In some cases, the direct experience of an extreme event or even the perception of being threatened by climate change was enough to develop a mental disorder like depression, PTSD or climate-anxiety. In most cases, however, a combination of a direct exposure coupled with effects of determinants, e.g., the immediate living environment or the social environment and the predispositions, constituted the main risk factors. Salutogenic and pathogenic determinants were identified which are associated with resilience building and impairment, respectively. The knowledge of these determinants may help to strengthen individual and societal resilience. The current study suggests which of the determinants are relevant and identifies those which are still neglected and should be further considered and analyzed. Thereby, we emphasize to focus more on social and personal determinants in the context of most hazards. Additionally, the analysis of the individual effect sizes of each determinant should be intensified. This offers a better understanding of the impact of determinants and helps in the interpretation of complex multivariate analyses. Furthermore, a common framework including definitions of methods and thresholds for each hazard, outcome and determinant respectively should be anticipated.

Children and adolescents were identified as a particular vulnerable group. To protect this group capacities must be built to be better prepared for climate change related extreme events, which are projected to significantly increase in the future. The research field on climate change and mental health has been growing recently but more research is necessary to better understand the connections.

## Data Availability

The original contributions presented in the study are included in the article/**Supplementary Material**. Further inquiries can be directed to the corresponding author.
