# Supplementary material for: Determinants and relationships of climate change, climate change hazards, mental health, and well-being: a systematic review
Source: Front Psychiatry. 2025 Aug 19;16:1601871. doi: 10.3389/fpsyt.2025.1601871 (PMC12402000; doi:10.3389/fpsyt.2025.1601871)
Supplement: Supplementary file 1 [file DataSheet1.pdf]

Supplementary Material

Contents

S1. The list of keywords used for the search strategy per search area 2

S2. Search syntax for each database.. 5

S3. Risk of bias assessment scales 19

S4. Keywords and disease categorised according to the used outcome groups 24

S5. Distribution and frequency of environmental and socio-individual determinants by outcome group for the hazards climate change, importance of the environment, flood, wildfire and meteorological variables and temperature extremes 25

S6. Basic characteristics of the studies selected 27

S1. The list of keywords used for the search strategy per search area

Outcomes

- anxiety
- anxiety disorder
- anxiety attack
- climate anxiety
- eco- anxiety
- emotional well-being
- human well-being
- well-being
- physical wellbeing
- psychological well-being
- mental *
  - disorder
  - health
  - illness
  - issue
  - problem
  - strain
  - stress
  - weakness
  - wellness
- psychological *
  - disorder
  - illbeing
  - illness
  - issue
  - problem
  - stress
  - wellbeing
  - wellness
- psychosocial *
  - disability
  - disorder
  - health
  - wellbeing
- behaviour *
  - disorder
  - problems
- sleep *
  - comfort
  - disorder
  - disturbance
  - duration
  - quality
  - relaxation
- stress post-traumatic stress disorder / PTSD
- post-traumatic stress
- stress-related disease
- stress disorder
- post natural disaster trauma
- depression
- depressive episode
- major depressive disorder /MDD
- seasonal major repressive disorder
- depressive mood swings
- mood *
  - swings
  - change
- episodic mood disorder
- trauma
- cognitive deficits
- cognition
- cognitive *
  - function
  - dysfunction/malfunction
  - impairment
- psychiatric *
  - disorder
  - illness
  - issue
  - problem
- phobia
- poor sleep
- insomnia
- neurotic disorder
- psychosis
- solastalgia
- conduct disorder
- hyperactivity
- hyper sensitive
- happiness
- quality of life
- life quality
- physical stress
- grief
- sorrow
- sad
- sadness
- exhaustion
- lost
- lonely
- loneliness
- overwhelming
- overwhelmed
- attachment disorder
- aggressive behaviour
- adjustment problems
- learning problems
- exhaustion
- panic
- sense of safety
- sense of security
- psychological restoration
- mental restoration

Environmental Determinants

**Physical surrounding**	- surrounding greenery<br>- (urban) green space<br>- urban green<br>- natural space<br>- green belt<br>- vegetation<br>- forest<br>- parks<br>- green infrastructure<br>- natural environment<br>- forest area<br>- natural park	- woodland<br>- natural forest crops<br>- agricultural area<br>- livestock waste dumps<br>- land use mix<br>- blue space<br>- (urban) wetland<br>- (urban) water body<br>- blue infrastructure<br>- pond<br>- lake<br>- beach	- coastal water<br>- communal pool<br>- river<br>- river bed built-up area<br>- built environment<br>- grey space<br>- streets<br>- neighbourhood quality<br>- landscape surroundings<br>- surrounding area<br>- living area<br>- housing area

**Education/learning**	- education<br>- education level<br>- learning	- educational attainment<br>- educational status<br>- uneducated

**Housing/living**	- living situation<br>- housing situation<br>- ownership rights<br>- ownership status<br>- house location<br>- living space<br>- ventilation	- air conditioning/ air cooling<br>- Neighbourhood conditions<br>- household *<br>• type (single person, multi-person, institution)<br>• size

**Recreation/playing**	- exercise<br>- sport<br>- playing	- hobbies<br>- physical activity<br>- leisure time activity

**Working/Employment**	- employment<br>- employment status<br>- employed<br>- unemployed<br>- under employment<br>- retired/retirement	- * labour<br>• manual<br>• non manual<br>• technical<br>- occupation<br>- occupational social class	- outdoor worker<br>- commuter<br>- office worker<br>- occupational status<br>- work alienation<br>- work contract<br>- profession

**Moving/Modes of Transportation**	- cycling<br>- running<br>- * transport<br>• active<br>• public<br>• passive<br>- Choice of Transportation	- travel by foot<br>- active movement<br>- physical activity behaviour<br>- travel behaviour<br>- walking<br>- travel time<br>- independent mobility<br>- independent travel

**Shopping/consumption**	- goods<br>- subsistence	- consumption pattern<br>- household consumption

Socio-individual Determinants

**Economic Determinants**	- income<br>- salary level<br>- financial stress	- financial strains<br>- economic strains<br>- unemployment rate<br>- livelihood

**Social Determinants**	- civil status<br>- marital status<br>- community *<br>• network<br>• support<br>• participation<br>• interaction<br>- support system<br>- isolation<br>- sense of loneliness<br>- parental<br>• anxiety<br>• stress<br>• depression	- society<br>- social *<br>• connections<br>• support<br>• relations<br>• well-being<br>• network<br>• isolation<br>- family *<br>• situation<br>• instability<br>• stability<br>• history<br>• size<br>- lifestyle

**Personal Determinants**	- genetics<br>- inherited disorder<br>- motivation<br>- illness<br>- awareness<br>- Environmental<br>• Concern<br>• sensibility<br>• value	- age<br>- sex/gender<br>- prematurity<br>- low birth weight<br>- breastfeeding<br>- formula feeding<br>- beliefs<br>- Connectedness with nature

**Others**	- socio-economic status/ SES<br>- social *<br>• stand<br>• status<br>• class<br>• position

S2. Search syntax for each database

	String	Results
	Embase	
#1	exp animal experiment/ or exp animal model/ or exp experimental animal/ or exp transgenic animal/ or exp male animal/ or exp female animal/ or exp juvenile animal/ or animal/ or chordata/ or vertebrate/ or tetrapod/ or exp fish/ or amniote/ or exp amphibia/ or mammal/ or exp reptile/ or exp sauropsid/ or therian/ or exp monotreme/ or placental mammal/ or exp marsupial/ or Euarchontoglires/ or exp Afrotheria/ or exp Boreoeutheria/ or exp Laurasiatheria/ or exp Xenarthra/ or primate/ or exp Dermoptera/ or exp Glires/ or exp Scandentia/ or Haplorhini/ or exp prosimian/ or simian/ or exp tarsiiform/ or Catarrhini/ or exp Platyrrhini/ or ape/ or exp Cercopithecidae/ or hominid/ or exp hylobatidae/ or exp chimpanzee/ or exp gorilla/ or exp orang utan/ or exp cephalopod/ or (rat or rats or animal or animals or mice or "in vivo" or mouse or rabbit or rabbits or murine or pig or pigs or dog or dogs or bovine or fish or vertebrate or vertebrates or cat or cats or rodent or rodents or mammal or mammals or chicken or chickens or monkey or monkeys or sheep or canine or canines or porcine or cattle or bird or birds or hamster or hamsters or primate or primates or cow or cows or chick or horse or horses or avian or avians or calf or swine or swines or xenopus or turkeys or bear or bears or frog or frogs or zebrafish or goat or goats or equine or calves or poultry or macaque or macaques or mole or moles or ovine or lamb or lambs or fishes or diptera or amphibian or amphibians or snake or snakes or ruminant or ruminants or hen or hens or piglet or piglets or feline or felines or simian or simians or laevis or trout or trouts or teleost or teleosts or salmon or salmons or seal or seals or bull or bulls or ewe or ewes or hedgehog or hedgehogs or macaca or macacas or proteus or pigeon or pigeons or bat or bats or duck or ducks or chimpanzee or chimpanzees or baboon or baboons or deer or deers or rana or ranas or carp or carps or heifer or swallow or swallows or lizard or lizards or canis or sow or sows or cynomolgus or quail or quails or reptile or reptiles or turtle or turtles or buffalo or gerbil or gerbils or boar or boars or squirrel or squirrels or oncorhynchus or mus or toad or toads or fowl or fowls or rerio or danio or ara or aras or musculus or tadpole or tadpoles or mulatta or salmo or ram or eagle or eagles or ferret or ferrets or goldfish or catfish or whale or whales or fox or foxes or ape or apes or elephant or elephants or bos or marmoset or marmosets or cod or cods or shark or sharks or wolf or eel or eels or auratus or rattus or zebra or zebras or tilapia or tilapias or gilt or camel or camels or squid or gallus or marsupial or marsupials or vole or voles or fascicularis or ovis or salmonid or salmonids or tiger or tigers or dolphin or dolphins or robin or robins or carpio or opossum or opossums or cyprinus or salamander or salamanders or felis or mink or minks or swan or swans or norvegicus or bufo or torpedo or bass or lamprey or lampreys or sus or python or pythons or tetrapod or tetrapods or shrew or shrews or lion or lions or hog or hogs or songbird or songbirds or oreochromis or starling or starlings or caprine or carassius or owl or owls or newt or newts or papio or scrofa or hare or hares or gorilla or gorillas or flounder or flounders or goose or herring or herrings or therian or buffaloes or canary or sparrow or sparrows or microtus or octopus or troglodytes or tuna or amphibia or chinchilla or chinchillas or ide or oryzias or cervus or kangaroo or kangaroos or armadillo or armadillos or callithrix or "pan troglodytes" or saimiri or cichlid or cichlids or donkey or donkeys or bream or char or chars or finch or raccoon or raccoons or bothrops or anguilla or perch or cricetus or seabird or seabirds or buck or bucks or naja or coturnix or salmonids or geese or minnow or minnows or raptor or raptors or merione or meriones or rodentia or elaphus or amniote or amniotes or elasmobranch or emu or emus or peromyscus or hominid or hominids or bubalus or crotalus or gull or gulls or anas or anura or lemur or lemurs or crow or crows or camelus or gibbon or gibbons or waterfowl or parrot or parrots or eels or cob or stickleback or sticklebacks or columba or mesocricetus or ambystoma or raven or ravens or gadus or penguin or penguins or orangutan or orangutans or sturgeon or sturgeons or cuniculus or aves or virginianus or cephalopod or cephalopods or cebus or sparus or tortoise or tortoises or guttata or morhua or unguiculatus or dogfish or vulpes or mallard or mallards or apodemus or alligator or alligators or oryctolagus or llama or llamas or reindeer or mustela or duckling or ducklings or wolves or sander or amazona or zebu ORbadger or badgers or dove or doves or ictalurus or capra or capras or equus or camelid or camelids or poecilia or mule or mules or perciformes or salvelinus or labrax or cyprinidae or ariidae or crocodile or crocodiles or fundulus or dicentrarchus or clarias or cercopithecus or chiroptera or alpaca or alpacas or pike or pikes or paralichthys or puma or pumas or didelphis or pisces or macropus or triturus or bison or bisons or epinephelus or gasterosteus or panthera or acipenser or mackerel or mackerels or tamarin or tamarins or ostrich or anolis or vervet or vervets or wallaby or glareolus or beaver or beavers or dromedary or catus or killifish or pimephales or promelas or aotus or phoca or panda or pandas or porpoise or porpoises or myotis or yak or yaks or agkistrodon or vipera or otter or otters or turbot or turbots or squamate or carnivora or mullet or mullets or hawk or hawks or taeniopygia or seahorse or seahorses or "poecilia reticulata" or falcon or falcons or prosimian or prosimians or parus or perca or fingerling or fingerlings or antelope or antelopes or tupaia or passeriformes or sepia or saguinus or coyote or coyotes or pongo or meleagris or reptilia or lepus or psittacine or hagfish or warbler or warblers or "russell s viper" or "russell s vipers" or smolt or smolts or budgerigar or sardine or sardines or cavia or cavias or hyla or pleurodeles or siluriformes or "great tit" or "great tits" or guppy or bonobo or bonobos or rutilus or trichosurus or muridae or phodopus or channa or squalus or lynx or sturnus or petromyzon or vitulina or monodelphis or cuttlefish or adder or adders or lepomis or canaria or gambusia or guppies or xiphophorus or flatfish or koala or koalasOR labeo or stingray or stingrays or chelonia or lampetra or spermophilus or crocodilian or "passer domesticus" or sciurus or artiodactyla or ranidae or corvus or necturus or platypus or canaries or bovid or lagopus or trimeresurus or gariepinus or marten or martens or drosophilidae or mugil or sunfish or porcellus or cypriniformes or alouatta or scophthalmus or anser or electrophorus or putorius or iguana or iguanas or lama or lamas or takifugu or circus or eptesicus or flycatcher or galago or galagos or Trachemys or lungfish or characiformes or shorebird or shorebirds or giraffe or giraffes or micropterus or scyliorhinus or cichlidae or loligo or porcupine or porcupines or chub or chubs or solea or pleuronectes or hylidae or viperidae or echis or sorex or anchovy or lagomorph or ostriches or vulture or vultures or whitefish or Araneus or jird or jirds or tern or esox or drake or drakes or elapidae or gallopavo or chordata or myodes or caretta or serinus or grouse or misgurnus or meles or blackbird or blackbirds or coregonus or bobwhite or bobwhites or heteropneustes or mammoth or mammoths or turdus or rhinella or ateles or characidae or clupea or bungarus or brill or "Struthio camelus" or sloth or sloths or pteropus or sculpin or anthropoids or pollock or pollocks or morone or "pan paniscus" or litoria or chipmunk or chipmunks or balaenoptera or marmota or melopsittacus or hyrax or lemming or lemmings or halibut or hylobates or lates or caiman or caimans or sigmodon or stenella or barbel or barbels or sterna or parakeet or parakeets or phocoena or leptodactylus or canidae or buteo or harengus or gopher or gophers or marmot or marmots or gosling or goslings or platichthys or gar or gars or sebastes or marsupialia or notophthalmus or gazelle or gazelles or insectivora or paridae or felidae or russula or galliformes or bombina or colobus or echidna or echidnas or seabass or syncerus or plaice or "blue tit" or "blue tits" or pagrus or catfishes or cetacea or barbus or cygnus or ficedula or chamois or colubridae or perches or coelacanth or fitch or urodela or cynops or martes or halichoerus or aix or salmonidae or leuciscus or magpie or magpies or silurus or whiting or whitings or Anseriformes or colinus or rhea or chlorocebus or octodon or acinonyx or mouflon or mouflons or ibex or tetraodon or bufonidae or equidae or jackal or cephalopoda or dendroaspis or glama or muskrat or muskrats or sable or sables or wildebeest or streptopelia or albifrons or vespertilionidae or woodpecker or woodpeckers or muntjac or muntjacs or archosaur or branta or cricetulus or megalobrama or poeciliidae or desmodus or snakehead or snakeheads or tench or teal or teals or bandicoot or bandicoots or apteronotus or phyllostomidae or crocidura or buzzard or buzzards or larimichthys or cercocebus or pipistrellus or erithacus or impala or impalas or rousettus or haddock or haddocks or tinca or ratite or calidris or cynoglossus or hypophthalmichthys or bullock or bullocks or dromedaries or alectoris or filly or salamandra or cingulata or bitis or grus or ammodytes or macaw or macaws or hypoleuca or sapajus or cyprinodontiformes or hippopotamus or pelophylax or capybara or capybaras or weasel or weasels or cairina or cynomys or lutra or cockatoo or cockatoos or lachesis or lagomorpha or rupicapra or daboia or "orang utan" or "orang utans" or platyrrhini or charadriiformes or micrurus or psittaciformes or spalax or loris or mustelidae or sylvilagus or vitticeps or cockatiel or mustelus or cottus or erythrocebus or dipodomys or platessa or callicebus or loricariidae or catostomus or cuneata or cyanistes or cyprinodon or sigmodontinae or elasmobranchii or Trichechus or sauropsid or xenarthra or dormouse or perissodactyla or nautilus or cirrhinus or gulo or gulos or tragelaphus or merula or numida or sciaenidae or cerastes or sciuridae or gibbosus or octopuses or eland or elands or phyllomedusa or pogona or walrus or agamidae or leptodactylidae or ridibundus or leontopithecus or anteater or anteaters or pelodiscus or cebidae or columbianus or "pelteobagrus fulvidraco" or hominoidea or mandrillus or "zonotrichia leucophrys" or agama or gobiocypris or "bearded dragon" or "bearded dragons" or sarotherodon or talpa or discoglossus or	8223152

	hagfishes or sphenodon or gudgeon or amphiuma or aythya or tenrec or tenrec or hominidae or risoria or salamandridae or camelidae or columbiformes or latimeria or plover or plovers or afrotheria or "falco sparverius" or polecat or polecats or crotalinae or salvadora or tarsier or lucioperca or anchovies or lungfishes or terrapin or "dromaius novaehollandiae" or lateolabrax or eigenmannia or pelamis or theropithecus or murinae or gander or gymnotus or pseudacris or gymnophiona or gymnotiformes or laticauda or falconiformes or dugong or dugongs or pintail or pintails or rook or rooks or lasiurus or catshark or catsharks or micropogonias or "red junglefowl" or paddlefish or ophiophagus or hollandicus or nymphicus or pimelodidae or aepyceros or cobitidae or strigiformes or cobitis or dormice or alytes or calloselasma or guanaco or guanacos or phasianidae or "round goby" or trichogaster or catarrhini or eelpout or eelpouts or galaxias or gaur or pungitius or suslik or susliks or flatfishes or percidae or caprinae or todarodes or osmerus or ameiurus or anthropoidea or "castor canadensis" or pouting or poutings or tetraodontiformes or arvicolinae or siamang or siamangs or "castor fiber" or nomascus or "red knot" or "red knots" or syngnathidae or iguanidae or eretmochelys or ursidae or callimico or columbidae or microhylidae or anaxyrus or menidia or pipistrelle or greylag or pipidae or scandentia or bowfin or bowfins or dendrobatidae or zenaida or bushbaby or harrier or harriers or macropodidae or pygerythrus or clupeidae or odorrana or corvidae or jerboa or jerboas or canutus or hylobatidae or clupeiformes or "great cormorant" or "great cormorants" or scorpaeniformes or chondrostean or garfish or proboscidea or psetta or diapsid or serotinus or tetrao or walruses or carcharhiniformes or leucoraja or pumpkinseed or dosidicus or acipenseriformes or daubentonii or emberizidae or gadiformes or hyraxes or stizostedion or wolverine or wolverines or lissotriton or acanthurus or centrarchidae or gloydius or laurasiatheria or limosa or psittacula or leporidae or proteidae or zander or zanders or arapaima or bagridae or cyprinodontidae or mithun or pandion or jackdaw or jackdaws or procyonidae or carus or jaculus or salmoniformes or "common sole" or "common soles" or protobothrops or calamita or brachyteles or trionyx or turdidae or boidae or luscinia ORpugnax or euarchontoglires or saithe or saithes or symphalangus or aardvark or aardvarks or oystercatcher or oystercatchers or arius or corydoras or poacher or poachers or aurochs or cebuella or crecca or lemuridae or sirenia or lemmus or perdixOR glires or lepidosaur or muskox or deinagkistrodon or Pholidota or holocephali or cercopithecinae or clariidae or agapornis or doryteuthis or tyrannidae or dicroglossidae or godwit or godwits or monedula or pongidae or atheriniformes or colobinae or lophocebus or atelidae or cottidae or leucopsis or acanthuridae or didelphimorphia or elver or elvers or lapponica or dermoptera or "european hake" or "european hakes" or gerbillinae or banteng or hartebeest or hartebeests or hogget or haematopus or "anguis fragilis" or "grey heron" or "grey herons" or "blue whiting" or "blue whitings" or furnariidae or macrovipera or socidae or "lapwing OR lapwings OR mylopharyngodon OR wallabia OR beloniformes OR potoroo ORpotoroos OR athene noctua" or pleuronectidae or bushbabies or muscicapidae or alligatoridae or fuligula or "bush baby" or guineafowl or spoonbill or spoonbills or viverridae or catostomidae or zebrafishes or ibexes or vendace or estrildidae or monotremata or sepiella or ambystomatidae or shelduck or shelducks or treeshrew or treeshrews or hoplobatrachus or pochard or hoolock or hoolocks or lynxes or antilope or antilopes or blackbuck or blackbucks or cricetinae or paramisgurnus or skylark or skylarks or soleidae or allobates or "northern wheatear" or "northern wheatears" or pitheciidae or takin or theria or vanellus or galaxiidae or lorisidae or ostralegus or palaeognathae or "stone loach" or alauda or callitrichinae or caniformia or duttaphrynus or ictaluridae or osteoglossiformes or poultries or curema or "ruddy turnstone" or "ruddy turnstones" or sheatfish or sunfishes or centropomidae or hemachatus or platalea or thamnophilidae or "song thrush" or atherinopsidae or siluridae or tadorna or chroicocephalus or ermine or ermines or gavialis or ruff or tupaiidae or diprotodontia or hyaenidae or antilopinae or crocodylidae or herpestidae or hippopotamidae or "northern shoveler" or "round gobies" or cheirogaleidae or indriidae or fundulidae or pythonidae or rhynchocephalian or anodorhynchus or "red-backed shrike" or "red-backed shrikes" or triakidae or phalangeridae ORaoudad or boreoeutheria or "eurasian jay" or "eurasian jays" or feliformia or haplorhini or osteoglossidae or paenungulata or struthioniformes or ferina or sanderling or sanderlings or spheniscidae or cuttlefishes or cygnet or dasycneme or gadwall or gadwalls or "pelobates fuscus" or wryneck or wrynecks or afrosoricida or culaea or "dover sole" or "dover soles" or paralichthyidae or passeridae or osteolaemus or "song thrushes" or bluethroat or bluethroats or hydrophiidae or megrim or mephitidae or strepsirhini or tomistoma or epidalea or osmeriformes or "bush babies" or tarsiiform or atelinae or bufotes or "eurasian coot" or "eurasian coots" or galagidae or geopelia or philomachus or tubulidentata or bombinatoridae or pelobatidae or tachysurus or ailuridae or woodlark or woodlarks or alcelaphinae or redshank or redshanks or salientia or "sand smelt" or "sand smelts" or woodmice or woodmouse or dasyproctidae or "eurasian wigeon" or "eurasian wigeons" or garganey or garganeys or "lemon sole" or "lemon soles" or "common dab" or "common dabs" or graylag or graylags or leucorodia or osphronemidae or bewickii or "common moorhen" or "common moorhens" or decapodiformes or gobbler or gobblers or odontophoridae or paddlefishes or eutheria or salmonine or esociformes or "eurasian woodcock" or "eurasian woodcocks" or "european smelt" or "european smelts" or goldfishes or tenches or tyranni or "common chaffinch" or "common chaffinchs" or "common redstart" or "common redstarts" or "common roach" or "common roachs" or "great knot" or "great knots" or potoroidae or alytidae or coregonine or dipteral or leveret or "poeciliopsis gracilis" or amphiumidae or batrachoidiformes or "bighead goby" or heteropneustidae or lullula or "norway pout" or "norway pouts" or sipunculida or dogfishes or sebastidae or tarsiidae or alethinophidia or "common nase" or "common nases" or "common sandpiper" or "common sandpipers" or "eurasian blackcap" or "eurasian blackcaps" or pterocnemia or syngnathiformes or "common chaffinches" or eupleridae or octopodiformes or phascolarctidae or scophthalmidae or "starry smooth-hound" or "starry smooth-hounds" or whitefishes or cuniculidae or "european sprat" or "european sprats" or "rosy bitterling" or "rosy bitterlings" or "common dace" or "common daces" or "lesser weever" or "lesser weevers" or scaldfish or "water rail" or "water rails" or alouattinae or centrarchiformes or "common whitethroat" or "common whitethroats" or gavialidae or "grey gurnard" or "grey gurnards" or lateolabracidae or rheiformes or "tub gurnard" or "tub gurnards" or "common chiffchaff" or "common chiffchaffs" or garfishes or "lesser whitethroat" or "lesser whitethroats" or myoxidae or seabasses or spariformes or umbridae or "yellow boxfish" or anabantiformes or aotidae or "common bleak" or "common bleaks" or "common rudd" or "common rudds" or "greater pipefish" or hapale or nandiniidae or "stone loaches" or whinchat or whinchats or acanthuriformes or "brotula barbata" or "common ling" or "common lings" or "common roaches" or cottonrat or cottonrats or douroucoulis or dromaiidae or fitches or fitchew or galaxiiformes or laprine or saimiriinae or solenette or tarsii or "tompot blenny" or "common dragonet" or "common dragonets" or "longspined bullhead" or "longspined bullheads" or monotremate or monotremates or pempheriformes or perdicinae or presbytini or smegmamorpha or "bighead gobies" or "carangaria incertae sedis" or coiidae or "fivebeard rockling" or foulmart or foumart or grasskeet or "greater pipefishes ORibices" or millionfish or muguliformes or "Norwegian topknot" or peewit or "red sea sailfin tang" or rupicapras or sheatfishes or "tompot blennies" or "twait shad" or "yellow boxfishes").ti,ab,kw.	
**#2**	("well?being" or "psychological restoration" or "mental restoration" or "life adj2 quality" or "life adj2 satisfaction" or ("mental*" or "psychological*" or "psychos*" or "psychiatric*") or "sleep*" or "insomnia" or "post-traumatic*" or "PTSD" or "trauma" or "physical adj2 stress*" or "psychological adj2 stress*" or "mental adj2 stress*").ti,ab,kw. or "stress disorder*".mp. or "stress disease*".ti,ab,kw. or "depressi*".ti,ab,kw. or "MDD".ti,ab,kw. or "mood*".ti,ab,kw. or ("cognitive*" or "cognition").ti,ab,kw. or "disorder*".ti,ab,kw. or "mania".ti,ab,kw. or "phobia".ti,ab,kw. or "anxiet*".ti,ab,kw. or "behavio* adj2 disorder".ti,ab,kw. or "behavio* adj2 problem*".ti,ab,kw. or "behavio* adj2 change*".ti,ab,kw. or "hyperactiv*".ti,ab,kw. or "hyper sensitive".ti,ab,kw. or ("grief" or "sorrow" or "sad" or "sadness" or "fear" or "anger" or "frustration" or "guilt" or "helpless*" or "powerless*" or "exhaustion" or "lost" or "lonel*" or "overwhelm*" or "panic*").ti,ab,kw. or "sense of safety".ti,ab,kw. or "sense of security".ti,ab,kw. or "happiness".ti,ab,kw. or "solastalgia".ti,ab. or ("adjustment problem*" or "learning problem*").ti,ab,kw. or exp "mental health"/ or "anxiety disorders"/ or "mood disorders"/ or "quality of life"/ or "satisfaction"/ or exp "sleep quality"/ or exp "emotional well-being"/ or exp "physical well-being"/ or exp "social well-being"/ [mp=title, abstract, heading word, drug trade name, original title, device manufacturer, drug manufacturer, device trade name, keyword heading word, floating subheading word, candidate term word]	5003693
**#3**	("climate change*" or "global warming" or "climate variability*" or "anthropogenic warming" or "environmental change*" or "climatic change*" or "burning fossil fuel*" or "atmospheric warm*" or "increased humidity" or "climate hazard*" or "climate crisis" or "rising temperature*" or "precipitation change*" or "sea level rise" or "environmental impact*" or "environmental disaster*" or "climate adaptation*" or "albedo change*" or "rising sea levels" or "earth warming" or "temperature change*" or "environmental amenit*" or "environmental dynamic*" or "increased CO2 emission*" or "carbon dioxide emission*" or "methane emission*" or "CH4 emission*" or "hot climate*" or "NO2 emission*" or "nitrogen dioxide emission*" or "warming planet" or "north Atlantic oscillation*" or "NAO" or "humidity change*" or "increased radiative forcing" or "radiative forcing scenario*" or "dirty fuel*" or "atmospheric blocking" or "fossil fuel combustion*" or "urban	549579

	heat island" or "UHI" or "environmental stressor" or "heat wave*" or "cold wave*" or "drought*" or "flood*" or ("wildfire*" or "bushfire*" or "forest fire*") or "heavy precipitation" or "extreme weather event*" or "dust storm*" or "dry period*" or "extreme meteorological event*" or "allergen" or "smog" or "sand storm*" or "heavy snow" or "extreme heat event*" or "aridity" or "air pollution*" or "Ozone" or "particulate matter" or "PM" or "PM2.5" or "ozone*" or "natural disasters" or "particulate matter").ti,ab,kw. or exp "climate change"/ or exp "air pollution"/ or exp "extreme weather"/ (549579)	
**#4**	("Europ*" or "EU" or "European Union" or "continental Europe" or "European region*" or "European countr*").ti,ab,kw. or exp "Europe"/	2146963
**#5**	("surrounding*" or "natural space*" or "natural environment" or "park*" or "green*" or "vegetation" or "forest*" or "woodland*" or "meadow*" or "grassland" or "shrubland" or "crop*" or "agricultural area*" or "cultivated land*" or "land use mix" or "blue*" or "wetland*" or ("water body" or "water bodies") or "pond*" or "lake*" or "beach*" or "coastal water*" or "pool" or "inland-blue space" or "river*" or "canal*" or "coastal-blue space*" or "esplanade*" or ("built environment*" or "built?up area*") or "grey space*" or "street*" or "residential area*" or ("housing adj2 area*" or "neighbo?rhood*" or "living adj2 area*") or "livestock*" or "waste dump*").ti,ab,kw. or exp "built environment"/ or exp "fresh water"/	1732883
**#6**	("education*" or "learning").ti,ab,kw. or exp "education"/	2381376
**#7**	("living situation" or "housing situation" or "ownership right*" or "ownership status" or "house*" or "household*" or "living adj2 space" or ("air condition*" or "air cooling") or "heating" or "ventilation").ti,ab,kw. or exp "residence characteristics"/ or exp "home environment"/	586840
**#8**	("exercise*" or "sport*" or "playing" or " hobb*" or "physical* activ*" or "leisure" or "leisure time activity" or "recreation*").ti,ab,kw. or exp "leisure"/	854445
**#9**	("employ*" or "unemploy*" or "under?employment" or "work contract*" or "retire*" or "profession*" or "labo?r*" or "occupation*" or "work alienation" or "outdoor worker*" or "commuter*" or "office worker*").ti,ab,kw. or exp "employment"/	2712291
**#10**	("Cycling" or "running" or ("active transport*" or "public transport" or "passive transport") or "travel*" or "active movement*" or "walking" or "travel adj2 time" or "choice of transportation" or ("independent mobility" or "independent travel")).ti,ab,kw.	404525
**#11**	("goods" or "subsistence*" or "consumption*").ti,ab,kw.	473577
**#12**	("Income" or "salary adj2 level" or "financial*" or "economic strains" or "unemployment adj2 rate*" or "livelihood*").ti,ab,kw. or exp "income"/ or exp "economic status"/ or exp "income group"/	561702
**#13**	("civil status" or "marital status" or "societ*" or "communit*" or "support*" or "social*" or "famil*" or "lifestyle" or "isolation" or "sense of loneliness" or ("parental anxiety*" or "parental stress" or "parental depression*")).ti,ab,kw. or exp "social environment"/ or exp "social class"/	6991814
**#14**	(genetic* or "inherited*" or motivation* or illness* or awareness or "allerg*" or "breastfeed*" or "formula feeding" or "belief*" or "age" or ("sex" or "gender") or ("prematurity" or "birth weight") or "environmental value*" or "environmental concern*" or "environmental sensitivity" or "connectedness with nature").ti,ab,kw. or exp "family structure"/ or exp "family separation"/	7281051
**#15**	("socio-economic status" or "SES").ti,ab,kw.	44449
**#16**	#5 or #6 or #7 or #8 or #9 or #10 or #11 or #12 or #13 or #14 or #15	16657032
**#17**	#2 and #3 and #4	4056
**#18**	#16 and #17	3382
**#19**	limit #18 to dd=20110101-20230131	1773
**#20**	limit #18 to rd=20110101-20230131	1371
**#21**	#19 or #20	3144
**#22**	#21 not #1	2730
**#23**	limit #22 to english language	2491
**#24**	#23 not ("systematic review" or "review").mp. [mp=title, abstract, heading word, drug trade name, original title, device manufacturer, drug manufacturer, device trade name, keyword heading word, floating subheading word, candidate term word]	2106
**#25**	#24 not ("RCT" or "randomi?ed control trail").mp. [mp=title, abstract, heading word, drug trade name, original title, device manufacturer, drug manufacturer, device trade name, keyword heading word, floating subheading word, candidate term word]	2096
**#26**	limit #25 to "remove medline records"	904
Medline		
**#1**	exp animal experimentation/ or exp models, animal/ or Animals.sh. or exp animal population groups/ or chordata.sh. or vertebrates.sh. or exp amphibians/ or exp birds/ or exp fishes/ or exp reptiles/ or mammals.sh. or primates.sh. or eutheria.sh. or exp artiodactyla/ or exp carnivora/ or exp cephalopoda/ or exp cetacea/ or exp chiroptera/ or exp elephants/ or exp hyraxes/ or exp insecta/ or exp lagomorpha/ or exp marsupialia/ or exp monotremata/ or exp perissodactyla/ or Proboscidea Mammal.sh. or exp rodentia/ or exp scandentia/ or exp sirenia/ or haplorhini.sh. or exp strepsirhini/ or exp platyrrhini/ or exp tarsii/ or catarrhine.sh. or exp cercopithecidae/ or exp hylobatidae/ or Hominidae.sh. or exp gorilla gorilla/ or exp pan paniscus/ or exp pan troglodytes/ or exp pongo/ or (rat or rats or animal or animals or mice or "in vivo" or mouse or rabbit or rabbits or murine or pig or pigs or dog or dogs or bovine or fish or vertebrate or vertebrates or cat or cats or rodent or rodents or mammal or mammals or chicken or chickens or monkey or monkeys or sheep or canine or canines or porcine or cattle or bird or birds or hamster or hamsters or primate or primates or cow or cows or chick or horse or horses or avian or avians or calf or swine or swines or xenopus or turkeys or bear or bears or frog or frogs or zebrafish or goat or goats or equine or calves or poultry or macaque or macaques or mole or moles or ovine or lamb or lambs or fishes or diptera or amphibian or amphibians or snake or snakes or ruminant or ruminants or hen or hens or piglet or piglets or feline or felines or simian or simians or laevis or trout or trouts or teleost or teleosts or salmon or salmons or seal or seals or bull or bulls or ewe or ewes or hedgehog or hedgehogs or macaca or macacas or proteus or pigeon or pigeons or bat or bats or duck or ducks or chimpanzee or chimpanzees or baboon or baboons or deer or deers or rana or ranas or carp or carps or heifer or swallow or swallows or lizard or lizards or canis or sow or sows or cynomolgus or quail or quails or reptile or reptiles or turtle or turtles or buffalo or gerbil or gerbils or boar or boars or squirrel or squirrels or oncorhynchus or mus or toad or toads or fowl or fowls or rerio or danio or ara or aras or musculus or tadpole or tadpoles or mulatta or salmo or ram or eagle or eagles or ferret or ferrets or goldfish or catfish or whale or whales or fox or foxes or ape or apes or elephant or elephants or bos or marmoset or marmosets or cod or cods or shark or sharks or wolf or eel or eels or auratus or rattus or zebra or zebras or tilapia or tilapias or gilt or camel or camels or squid or gallus or marsupial or marsupials or vole or voles or fascicularis or ovis or salmonid or salmonids or tiger or tigers or dolphin or dolphins or robin or robins or carpio or opossum or opossums or cyprinus or salamander or salamanders or felis or mink or minks or swan or swans or norvegicus or bufo or torpedo or bass or lamprey or lampreys or sus or python or pythons or tetrapod or tetrapods or shrew or shrews or lion or lions or hog or hogs or songbird or songbirds or oreochromis or starling or starlings or caprine or carassius or owl or owls or newt or newts or papio or scrofa or hare or hares or gorilla or gorillas or flounder or flounders or goose or herring or herrings or therian or buffaloes or canary or sparrow or sparrows or microtus or octopus or troglodytes or tuna or amphibia or chinchilla or chinchillas or ide or oryzias or cervus or kangaroo or kangaroos or armadillo or armadillos or callithrix or "pan troglodytes" or saimiri or cichlid or cichlids or donkey or donkeys or bream or char or chars or finch or raccoon or raccoons or bothrops or anguilla or perch or cricetus or seabird or seabirds or buck or bucks or naja or coturnix or salmonids or geese or minnow or minnows or raptor or raptors or merione or meriones or rodentia or elaphus or amniote or amniotes or elasmobranch or emu or emus or peromyscus or hominid or hominids or bubalus or crotalus or gull or gulls or anas or anura or lemur or lemurs or crow or crows or camelus or gibbon or gibbons or waterfowl or parrot or parrots or eels or cob or stickleback or sticklebacks or columba or mesocricetus or ambystoma or raven or ravens or gadus or penguin or penguins or orangutan or orangutans or sturgeon or sturgeons or cuniculus or aves or virginianus or cephalopod or cephalopods or cebus or sparus or tortoise or tortoises or guttata or morhua or unguiculatus or dogfish or vulpes or mallard or mallards or apodemus or alligator or alligators or oryctolagus or llama or llamas or reindeer or mustela or duckling or ducklings or wolves or sander or amazona or zebu ORbadger or badgers or dove or doves or ictalurus or capra or capras or equus or camelid or camelids or poecilia or mule or	8194482

	mules or perciformes or salvelinus or labrax or cyprinidae or ariidae or crocodile or crocodiles or fundulus or dicentrarchus or clarias or cercopithecus or chiroptera or alpaca or alpacas or pike or pikes or paralichthys or puma or pumas or didelphis or pisces or macropus or triturus or bison or bisons or epinephelus or gasterosteus or panthera or acipenser or mackerel or mackerels or tamarin or tamarins or ostrich or anolis or vervet or vervets or wallaby or glareolus or beaver or beavers or dromedary or catus or killifish or pimephales or promelas or aotus or phoca or panda or pandas or porpoise or porpoises or myotis or yak or yaks or agkistrodon or vipera or otter or otters or turbot or turbots or squamate or carnivora or mullet or mullets or hawk or hawks or taeniopygia or seahorse or seahorses or "poecilia reticulata" or falcon or falcons or prosimian or prosimians or parus or perca or fingerling or fingerlings or antelope or antelopes or tupaia or passeriformes or sepia or saguinus or coyote or coyotes or pongo or meleagris or reptilia or lepus or psittacine or hagfish or warbler or warblers or "russell s viper" or "russell s vipers" or smolt or smolts or budgerigar or sardine or sardines or cavia or cavias or hyla or pleurodeles or siluriformes or "great tit" or "great tits" or guppy or bonobo or bonobos or rutilus or trichosurus or muridae or phodopus or channa or squalus or lynx or sturnus or petromyzon or vitulina or monodelphis or cuttlefish or adder or adders or lepomis or canaria or gambusia or guppies or xiphophorus or flatfish or koala or koalasOR labeo or stingray or stingrays or chelonia or lampetra or spermophilus or crocodilian or "passer domesticus" or sciurus or artiodactyla or ranidae or corvus or necturus or platypus or canaries or bovid or lagopus or trimeresurus or gariepinus or marten or martens or drosophilidae or mugil or sunfish or porcellus or cypriniformes or alouatta or scophthalmus or anser or electrophorus or putorius or iguana or iguanas or lama or lamas or takifugu or circus or eptesicus or flycatcher or galago or galagos or Trachemys or lungfish or characiformes or shorebird or shorebirds or giraffe or giraffes or micropterus or scyliorhinus or cichlidae or loligo or porcupine or porcupines or chub or chubs or solea or pleuronectes or hylidae or viperidae or echis or sorex or anchovy or lagomorph or ostriches or vulture or vultures or whitefish or Araneus or jird or jirds or tern or esox or drake or drakes or elapidae or gallopavo or chordata or myodes or caretta or serinus or grouse or misgurnus or meles or blackbird or blackbirds or coregonus or bobwhite or bobwhites or heteropneustes or mammoth or mammoths or turdus or rhinella or ateles or characidae or clupea or bungarus or brill or "Struthio camelus" or sloth or sloths or pteropus or sculpin or anthropoids or pollock or pollocks or morone or "pan paniscus" or litoria or chipmunk or chipmunks or balaenoptera or marmota or melopsittacus or hyrax or lemming or lemmings or halibut or hylobates or lates or caiman or caimans or sigmodon or stenella or barbel or barbels or sterna or parakeet or parakeets or phocoena or leptodactylus or canidae or buteo or harengus or gopher or gophers or marmot or marmots or gosling or goslings or platichthys or gar or gars or sebastes or marsupialia or notophthalmus or gazelle or gazelles or insectivora or paridae or felidae or russula or galliformes or bombina or colobus or echidna or echidnas or seabass or syncerus or plaice or "blue tit" or "blue tits" or pagrus or catfishes or cetacea or barbus or cygnus or ficedula or chamois or colubridae or perches or coelacanth or fitch or urodela or cynops or martes or halichoerus or aix or salmonidae or leuciscus or magpie or magpies or silurus or whiting or whitings or Anseriformes or colinus or rhea or chlorocebus or octodon or acinonyx or mouflon or mouflons or ibex or tetraodon or bufonidae or equidae or jackal or cephalopoda or dendroaspis or glama or muskrat or muskrats or sable or sables or wildebeest or streptopelia or albifrons or vespertilionidae or woodpecker or woodpeckers or muntjac or muntjacs or archosaur or branta or cricetulus or megalobrama or poeciliidae or desmodus or snakehead or snakeheads or tench or teal or teals or bandicoot or bandicoots or apteronotus or phyllostomidae or crocidura or buzzard or buzzards or larimichthys or cercocebus or pipistrellus or erithacus or impala or impalas or rousettus or haddock or haddocks or tinca or ratite or calidris or cynoglossus or hypophthalmichthys or bullock or bullocks or dromedaries or alectoris or filly or salamandra or cingulata or bitis or grus or ammodytes or macaw or macaws or hypoleuca or sapajus or cyprinodontiformes or hippopotamus or pelophylax or capybara or capybaras or weasel or weasels or cairina or cynomys or lutra or cockatoo or cockatoos or lachesis or lagomorpha or rupicapra or daboia or "orang utan" or "orang utans" or platyrrhini or charadriiformes or micrurus or psittaciformes or spalax or loris or mustelidae or sylvilagus or vitticeps or cockatiel or mustelus or cottus or erythrocebus or dipodomys or platessa or callicebus or loricariidae or catostomus or cuneata or cyanistes or cyprinodon or sigmodontinae or elasmobranchii or Trichechus or sauropsid or xenarthra or dormouse or perissodactyla or nautilus or cirrhinus or gulo or gulos or tragelaphus or merula or numida or sciaenidae or cerastes or sciuridae or gibbosus or octopuses or eland or elands or phyllomedusa or pogona or walrus or agamidae or leptodactylidae or ridibundus or leontopithecus or anteater or anteaters or pelodiscus or cebidae or columbianus or "pelteobagrus fulvidraco" or hominoidea or mandrillus or "zonotrichia leucophrys" or agama or gobiocypris or "bearded dragon" or "bearded dragons" or sarotherodon or talpa or discoglossus or hagfishes or sphenodon or gudgeon or amphiuma or aythya or tenrec or tenrec or hominidae or risoria or salamandridae or camelidae or columbiformes or latimeria or plover or plovers or afrotheria or "falco sparverius" or polecat or polecats or crotalinae or salvadora or tarsier or lucioperca or anchovies or lungfishes or terrapin or "dromaius novaehollandiae" or lateolabrax or eigenmannia or pelamis or theropithecus or murinae or gander or gymnotus or pseudacris or gymnophiona or gymnotiformes or laticauda or falconiformes or dugong or dugongs or pintail or pintails or rook or rooks or lasiurus or catshark or catsharks or micropogonias or "red junglefowl" or paddlefish or ophiophagus or hollandicus or nymphicus or pimelodidae or aepyceros or cobitidae or strigiformes or cobitis or dormice or alytes or calloselasma or guanaco or guanacos or phasianidae or "round goby" or trichogaster or catarrhini or eelpout or eelpouts or galaxias or gaur or pungitius or suslik or susliks or flatfishes or percidae or caprinae or todarodes or osmerus or ameiurus or anthropoidea or "castor canadensis" or pouting or poutings or tetraodontiformes or arvicolinae or siamang or siamangs or "castor fiber" or nomascus or "red knot" or "red knots" or syngnathidae or iguanidae or eretmochelys or ursidae or callimico or columbidae or microhylidae or anaxyrus or menidia or pipistrelle or greylag or pipidae or scandentia or bowfin or bowfins or dendrobatidae or zenaida or bushbaby or harrier or harriers or macropodidae or pygerythrus or clupeidae or odorrana or corvidae or jerboa or jerboas or canutus or hylobatidae or clupeiformes or "great cormorant" or "great cormorants" or scorpaeniformes or chondrostean or garfish or proboscidea or psetta or diapsid or serotinus or tetrao or walruses or carcharhiniformes or leucoraja or pumpkinseed or dosidicus or acipenseriformes or daubentonii or emberizidae or gadiformes or hyraxes or stizostedion or wolverine or wolverines or lissotriton or acanthurus or centrarchidae or gloydius or laurasiatheria or limosa or psittacula or leporidae or proteidae or zander or zanders or arapaima or bagridae or cyprinodontidae or mithun or pandion or jackdaw or jackdaws or procyonidae or carus or jaculus or salmoniformes or "common sole" or "common soles" or protobothrops or calamita or brachyteles or trionyx or turdidae or boidae or luscinia ORpugnax or euarchontoglires or saithe or saithes or symphalangus or aardvark or aardvarks or oystercatcher or oystercatchers or arius or corydoras or poacher or poachers or aurochs or cebuella or crecca or lemuridae or sirenia or lemmus or perdixOR glires or lepidosaur or muskox or deinagkistrodon or Pholidota or holocephali or cercopithecinae or clariidae or agapornis or doryteuthis or tyrannidae or dicroglossidae or godwit or godwits or monedula or pongidae or atheriniformes or colobinae or lophocebus or atelidae or cottidae or leucopsis or acanthuridae or didelphimorphia or elver or elvers or lapponica or dermoptera or "european hake" or "european hakes" or gerbillinae or banteng or hartebeest or hartebeests or hogget or haematopus or "anguis fragilis" or "grey heron" or "grey herons" or "blue whiting" or "blue whitings" or furnariidae or macrovipera or socidae or "lapwingORlapwingsORmylopharyngodonORwallabiaORbeloniformesORpotoroo ORpotoroosOR athene noctua" or pleuronectidae or bushbabies or muscicapidae or alligatoridae or fuligula or "bush baby" or guineafowl or spoonbill or spoonbills or viverridae or catostomidae or zebrafishes or ibexes or vendace or estrildidae or monotremata or sepiella or ambystomatidae or shelduck or shelducks or treeshrew or treeshrews or hoplobatrachus or pochard or hoolock or hoolocks or lynxes or antilope or antilopes or blackbuck or blackbucks or cricetinae or paramisgurnus or skylark or skylarks or soleidae or allobates or "northern wheatear" or "northern wheatears" or pitheciidae or takin or theria or vanellus or galaxiidae or lorisidae or ostralegus or palaeognathae or "stone loach" or alauda or callitrichinae or caniformia or duttaphrynus or ictaluridae or osteoglossiformes or poultries or curema or "ruddy turnstone" or "ruddy turnstones" or sheatfish or sunfishes or centropomidae or hemachatus or platalea or thamnophilidae or "song thrush" or atherinopsidae or siluridae or tadorna or chroicocephalus or ermine or ermines or gavialis or ruff or tupaiidae or diprotodontia or hyaenidae or antilopinae or crocodylidae or herpestidae or hippopotamidae or "northern shoveler" or "round gobies" or cheirogaleidae or indriidae or fundulidae or pythonidae or rhynchocephalian or anodorhynchus or "red-backed shrike" or "red-backed shrikes" or triakidae or phalangeridae ORaoudad or boreoeutheria or "eurasian jay" or "eurasian jays" or feliformia or haplorhini or osteoglossidae or paenungulata or struthioniformes or ferina or sanderling or sanderlings or spheniscidae or cuttlefishes or cygnet or dasycneme or gadwall or gadwalls or "pelobates fuscus" or wryneck or wrynecks or afrosoricida or culaea or "dover sole" or "dover soles" or paralichthyidae or passeridae or osteolaemus or "song thrushes" or bluethroat or bluethroats or hydrophiidae or megrim or mephitidae or strepsirhini or tomistoma or epidalea or osmeriformes or "bush babies" or tarsiiform or atelinae or bufotes or "eurasian coot" or "eurasian coots" or galagidae or geopelia or philomachus or tubulidentata or bombinatoridae or pelobatidae or	

	tachysurus or ailuridae or woodlark or woodlarks or alcelaphinae or redshank or redshanks or salientia or "sand smelt" or "sand smelts" or woodmice or woodmouse or dasyproctidae or "eurasian wigeon" or "eurasian wigeons" or garganey or garganeys or "lemon sole" or "lemon soles" or "common dab" or "common dabs" or graylag or graylags or leucorodia or osphronemidae or bewickii or "common moorhen" or "common moorhens" or decapodiformes or gobbler or gobblers or odontophoridae or paddlefishes or eutheria or salmonine or esociformes or "eurasian woodcock" or "eurasian woodcocks" or "european smelt" or "european smelts" or goldfishes or tenches or tyranni or "common chaffinch" or "common chaffinchs" or "common redstart" or "common redstarts" or "common roach" or "common roachs" or "great knot" or "great knots" or potoroidae or alytidae or coregonine or dipteral or leveret or "poeciliopsis gracilis" or amphiumidae or batrachoidiformes or "bighead goby" or heteropneustidae or lullula or "norway pout" or "norway pouts" or sipunculida or dogfishes or sebastidae or tarsiidae or alethinophidia or "common nase" or "common nases" or "common sandpiper" or "common sandpipers" or "eurasian blackcap" or "eurasian blackcaps" or pterocnemia or syngnathiformes or "common chaffinches" or eupleridae or octopodiformes or phascolarctidae or scophthalmidae or "starry smooth-hound" or "starry smooth-hounds" or whitefishes or cuniculidae or "european sprat" or "european sprats" or "rosy bitterling" or "rosy bitterlings" or "common dace" or "common daces" or "lesser weever" or "lesser weevers" or scaldfish or "water rail" or "water rails" or alouattinae or centrarchiformes or "common whitethroat" or "common whitethroats" or gavialidae or "grey gurnard" or "grey gurnards" or lateolabracidae or rheiformes or "tub gurnard" or "tub gurnards" or "common chiffchaff" or "common chiffchaffs" or garfishes or "lesser whitethroat" or "lesser whitethroats" or myoxidae or seabasses or spariformes or umbridae or "yellow boxfish" or anabantiformes or aotidae or "common bleak" or "common bleaks" or "common rudd" or "common rudds" or "greater pipefish" or hapale or nandiniidae or "stone loaches" or whinchat or whinchats or acanthuriformes or "brotula barbata" or "common ling" or "common lings" or "common roaches" or cottonrat or cottonrats or douroucoulis or dromaiidae or fitches or fitchew or galaxiiformes or laprine or saimiriinae or solenette or tarsii or "tompot blenny" or "common dragonet" or "common dragonets" or "longspined bullhead" or "longspined bullheads" or monotremate or monotremates or pempheriformes or perdicinae or presbytini or smegmamorpha or "bighead gobies" or "carangaria incertae sedis" or coiidae or "fivebeard rockling" or foulmart or foumart or grasskeet or "greater pipefishes ORibices" or millionfish or muguliformes or "Norwegian topknot" or peewit or "red sea sailfin tang" or rupicapras or sheatfishes or "tompot blennies" or "twait shad" or "yellow boxfishes").ti,ab,kw.	
**#2**	("well?being" or "psychological restoration" or "mental restoration" or "life adj2 quality" or "life adj2 satisfaction" or ("mental*" or "psychological*" or "psychos*" or "psychiatric*") or "sleep*" or "insomnia" or "post-traumatic*" or "PTSD" or "trauma" or "physical adj2 stress*" or "psychological adj2 stress*" or "mental adj2 stress*" or "stress disorder*" or "stress disease*" or "depressi*" or "MDD" or "mood*" or ("cognitive*" or "cognition") or "disorder*" or "mania" or "phobia" or "anxiet*" or "behavio* adj2 disorder" or "behavio* adj2 problem*" or "behavio* adj2 change*" or "hyperactiv*" or "hyper sensitive" or ("grief" or "sorrow" or "sad" or "sadness" or "fear" or "anger" or "frustration" or "guilt" or "helpless*" or "powerless*" or "exhaustion" or "lost" or "lonel*" or "overwhelm*" or "panic*") or "sense of safety" or "sense of security" or "happiness").ti,ab,kw. or "solastalgia".ti,ab. or ("adjustment problem*" or "learning problem*").ti,ab,kw. or exp "mental health"/ or exp "anxiety disorders"/ or exp "mood disorders"/ or exp "quality of life"/ or exp "personal satisfaction"/ or exp "behavioral symptoms"/ or exp "sleep"/	3348290
**#3**	("climate change*" or "global warming" or "climate variability*" or "anthropogenic warming" or "environmental change*" or "climatic change*" or "burning fossil fuel*" or "atmospheric warm*" or "increased humidity" or "climate hazard*" or "climate crisis" or "rising temperature*" or "precipitation change*" or "sea level rise" or "environmental impact*" or "environmental disaster*" or "climate adaptation*" or "albedo change*" or "rising sea levels" or "earth warming" or "temperature change*" or "environmental amenit*" or "environmental dynamic*" or "increased CO2 emission*" or "carbon dioxide emission*" or "methane emission*" or "CH4 emission*" or "hot climate*" or "NO2 emission*" or "nitrogen dioxide emission*" or "warming planet" or "north Atlantic oscillation*" or "NAO" or "humidity change*" or "increased radiative forcing" or "radiative forcing scenario*" or "dirty fuel*" or "atmospheric blocking" or "fossil fuel combustion*" or "urban heat island" or "UHI" or "environmental stressor" or "heat wave*" or "cold wave*" or "drought*" or "flood*" or ("wildfire*" or "bushfire*" or "forest fire*") or "heavy precipitation" or "extreme weather event*" or "dust storm*" or "dry period*" or "extreme meteorological event*" or "allergen" or "smog" or "sand storm*" or "heavy snow" or "extreme heat event*" or "aridity" or "air pollution*" or "Ozone" or "particulate matter" or "PM" or "PM2.5" or "ozone*" or "natural disasters" or "particulate matter").ti,ab,kw. or exp "climate change"/ or exp "air pollution"/ or exp "extreme weather"/	327127
**#4**	("Europ*" or "EU" or "European Union" or "continental Europe" or "European region*" or "European countr*").ti,ab,kw. or exp "Europe"/	1742086
**#5**	("surrounding*" or "natural space*" or "natural environment" or "park*" or "green*" or "vegetation" or "forest*" or "woodland*" or "meadow*" or "grassland" or "shrubland" or "crop*" or "agricultural area*" or "cultivated land*" or "land use mix" or "blue*" or "wetland*" or ("water body" or "water bodies") or "pond*" or "lake*" or "beach*" or "coastal water*" or "pool" or "inland-blue space" or "river*" or "canal*" or "coastal-blue space*" or "esplanade*" or ("built environment*" or "built?up area*") or "grey space*" or "street*" or "residential area*" or ("housing adj2 area*" or "neighbo?rhood*" or "living adj2 area*") or "livestock*" or "waste dump*").ti,ab,kw. or exp "environment, controlled"/ or exp "built environment"/ or exp "fresh water"/	1523195
**#6**	("education*" or "learning").ti,ab,kw. or exp "educational status"/	914853
**#7**	("living situation" or "housing situation" or "ownership right*" or "ownership status" or "house*" or "household*" or "living adj2 space" or ("air condition*" or "air cooling") or "heating" or "ventilation").ti,ab,kw. or exp "residence characteristics"/ or exp "home environment"/	433290
**#8**	("exercise*" or "sport*" or "playing" or " hobb*" or "physical* activ*" or "leisure" or "leisure time activity" or "recreation*").ti,ab,kw. or exp "leisure activities"/	696831
**#9**	("employ*" or "unemploy*" or "under?employment" or "work contract*" or "retire*" or "profession*" or "labo?r*" or "occupation*" or "work alienation" or "outdoor worker*" or "commuter*" or "office worker*").ti,ab,kw. or exp "employment"/	1806834
**#10**	("Cycling" or "running" or ("active transport*" or "public transport" or "passive transport") or "travel*" or "active movement*" or "walking" or "travel adj2 time" or "choice of transportation" or ("independent mobility" or "independent travel")).ti,ab,kw.	257307
**#11**	("goods" or "subsistence*" or "consumption*").ti,ab,kw.	320744
**#12**	("Income" or "salary adj2 level" or "financial*" or "economic strains" or "unemployment adj2 rate*" or "livelihood*").ti,ab,kw. or exp "income"/ or "economics".sh.	304397
**#13**	("civil status" or "marital status" or "societ*" or "communit*" or "support*" or "social*" or "famil*" or "lifestyle" or "isolation" or "sense of loneliness" or ("parental anxiety*" or "parental stress" or "parental depression*")).ti,ab,kw. or exp "social environment"/ or exp "social conditions"/ or exp "social class"/	4010067
**#14**	(genetic* or "inherited*" or motivation* or illness* or awareness or "allerg*" or "breastfeed*" or "formula feeding" or "belief*" or "age" or ("sex" or "gender") or ("prematurity" or "birth weight") or "environmental value*" or "environmental concern*" or "environmental sensitivity" or "connectedness with nature").ti,ab,kw. or exp "family characteristics"/ or exp "family relations"/ or exp "family separation"/	4541902
**#15**	("socio-economic status" or "SES").ti,ab,kw.	26560
**#16**	#5 or #6 or #7 or #10 or #11 or #12 or #13 or #14 or #15	9436671
**#17**	#2 and #3 and #4	2304
**#18**	#16 and #17	1812
**#19**	limit #18 to dt=20110101-20230131	1157
**#20**	limit #18 to rd=20110101-20230131	1602
**#21**	#19 or #20	1634
**#22**	#21 not #1	1414
**#23**	limit #22 to english language	1254

**#24**	#23 not ("systematic review" or "review").mp. [mp=title, book title, abstract, original title, name of substance word, subject heading word, floating sub-heading word, keyword heading word, organism supplementary concept word, protocol supplementary concept word, rare disease supplementary concept word, unique identifier, synonyms, population supplementary concept word, anatomy supplementary concept word]	1142
**#25**	#24 not ("RCT" or "randomi?ed control trail").mp. [mp=title, book title, abstract, original title, name of substance word, subject heading word, floating sub-heading word, keyword heading word, organism supplementary concept word, protocol supplementary concept word, rare disease supplementary concept word, unique identifier, synonyms, population supplementary concept word, anatomy supplementary concept word]	1138
	Web of Science	
**#1**	TS=(rat OR rats OR animal OR animals OR mice OR "in vivo" OR mouse OR rabbit OR rabbits OR murine OR pig OR pigs OR dog OR dogs OR bovine OR fish OR vertebrate OR vertebrates OR cat OR cats OR rodent OR rodents OR mammal OR mammals OR chicken OR chickens OR monkey OR monkeys OR sheep OR canine OR canines OR porcine OR cattle OR bird OR birds OR hamster OR hamsters OR primate OR primates OR cow OR cows OR chick OR horse OR horses OR avian OR avians OR calf OR swine OR swines OR xenopus OR turkeys OR bear OR bears OR frog OR frogs OR zebrafish OR goat OR goats OR equine OR calves OR poultry OR macaque OR macaques OR mole OR moles OR ovine OR lamb OR lambs OR fishes OR diptera OR amphibian OR amphibians OR snake OR snakes OR ruminant OR ruminants OR hen OR hens OR piglet OR piglets OR feline OR felines OR simian OR simians OR laevis OR trout OR trouts OR teleost OR teleosts OR salmon OR salmons OR seal OR seals OR bull OR bulls OR ewe OR ewes OR hedgehog OR hedgehogs OR macaca OR macacas OR proteus OR pigeon OR pigeons OR bat OR bats OR duck OR ducks OR chimpanzee OR chimpanzees OR baboon OR baboons OR deer OR rana OR ranas OR carp OR carps OR heifer OR swallow OR swallows OR lizard OR lizards OR canis OR sow OR sows OR cynomolgus OR quail OR quails OR reptile OR reptiles OR turtle OR turtles OR buffalo OR gerbil OR gerbils OR boar OR boars OR squirrel OR squirrels OR oncorhynchus OR mus OR toad OR toads OR fowl OR fowls OR rerio OR danio OR ara OR aras OR musculus OR tadpole OR tadpoles OR mulatta OR salmo OR ram OR eagle OR eagles OR ferret OR ferrets OR goldfish OR catfish OR whale OR whales OR fox OR foxes OR ape OR apes OR elephant OR elephants OR bos OR marmoset OR marmosets OR cod OR cods OR shark OR sharks OR wolf OR eel OR eels OR auratus OR rattus OR zebra OR zebras OR tilapia OR tilapias OR gilt OR camel OR camels OR squid OR gallus OR marsupial OR marsupials OR vole OR voles OR fascicularis OR ovis OR salmonid OR salmonids OR tiger OR tigers OR dolphin OR dolphins OR robin OR robins OR carpio OR opossum OR opossums OR cyprinus OR salamander OR salamanders OR felis OR mink OR minks OR swan OR swans OR norvegicus OR bufo OR torpedo OR bass OR lamprey OR lampreys OR sus OR python OR pythons OR tetrapod OR tetrapods OR shrew OR shrews OR lion OR lions OR hog OR hogs OR songbird OR songbirds OR oreochromis OR starling OR starlings OR caprine OR carassius OR owl OR owls OR newt OR newts OR papio OR scrofa OR hare OR hares OR gorilla OR gorillas OR flounder OR flounders OR goose OR herring OR herrings OR therian OR buffaloes OR canary OR sparrow OR sparrows OR microtus OR octopus OR troglodytes OR tuna OR amphibia OR chinchilla OR chinchillas OR ide OR oryzias OR cervus OR kangaroo OR kangaroos OR armadillo OR armadillos OR callithrix OR "pan troglodytes" OR saimiri OR cichlid OR cichlids OR donkey OR donkeys OR bream OR char OR chars OR finch OR raccoon OR raccoons OR bothrops OR anguilla OR perch OR cricetus OR seabird OR seabirds OR buck OR bucks OR naja OR coturnix OR salmonids OR geese OR minnow OR minnows OR raptor OR raptors OR merione OR meriones OR rodentia OR elaphus OR amniote OR amniotes OR elasmobranch OR emu OR emus OR peromyscus OR hominid OR hominids OR bubalus OR crotalus OR gull OR gulls OR anas OR anura OR lemur OR lemurs OR crow OR crows OR camelus OR gibbon OR gibbons OR waterfowl OR parrot OR parrots OR eels OR cob OR stickleback OR sticklebacks OR columba OR mesocricetus OR ambystoma OR raven OR ravens OR gadus OR penguin OR penguins OR orangutan OR orangutans OR sturgeon OR sturgeons OR cuniculus OR aves OR virginianus OR cephalopod OR cephalopods OR cebus OR sparus OR tortoise OR tortoises OR guttata OR morhua OR unguiculatus OR dogfish OR vulpes OR mallard OR mallards OR apodemus OR alligator OR alligators OR oryctolagus OR llama OR llamas OR reindeer OR Mustela OR duckling OR ducklings OR wolves OR sander OR amazona OR zebu OR badger OR badgers OR dove OR doves OR ictalurus OR capra OR capras OR equus OR camelid OR camelids OR poecilia OR mule OR mules OR perciformes OR salvelinus OR labrax OR cyprinidae OR ariidae OR crocodile OR crocodiles OR fundulus OR dicentrarchus OR clarias OR cercopithecus OR chiroptera OR alpaca OR alpacas OR pike ORpikes OR paralichthys OR puma OR pumas OR didelphis OR pisces OR Macropus OR triturus OR bison OR bisons OR epinephelus OR gasterosteus OR panthera OR acipenser OR mackerel OR mackerels OR tamarin OR tamarins OR ostrich OR anolis OR vervet OR vervets OR wallaby OR glareolus OR beaver OR beavers OR dromedary ORcatus ORkillifish ORpimephales ORpromelas OR aotus ORphoca ORpanda ORpandas ORporpoise OR porpoises OR myotis OR yak OR yaks OR agkistrodon OR vipera OR otter OR otters OR turbot OR turbots OR squamate OR carnivora OR mullet OR mulletsOR hawk OR hawks OR taeniopygia OR seahorse OR seahorses OR "poecilia reticulata" OR falcon OR falcons OR prosimian OR prosimians OR parus OR perca OR fingerling OR fingerlings OR antelope OR antelopes OR tupaia OR passeriformes OR sepia OR saguinus OR coyote OR coyotes OR pongo OR meleagris OR reptilia OR lepus OR psittacine OR hagfish OR warbler OR warblers OR "russell s viper" OR "russell s vipers" OR smolt OR smolts OR budgerigar OR sardine OR sardines OR cavia OR cavias OR hyla OR pleurodeles OR siluriformes OR "great tit" OR "great tits" OR guppy OR bonobo OR bonobos OR rutilus OR trichosurus OR muridae OR phodopus OR channa OR squalus OR lynx OR sturnus OR petromyzon OR vitulina OR monodelphis OR cuttlefish OR adder OR adders OR lepomis OR canaria OR gambusia OR guppies OR xiphophorus OR flatfish OR koala OR koalas OR labeo OR stingray OR stingrays OR chelonia OR lampetra OR spermophilus OR crocodilian OR "passer domesticus" OR sciurus OR artiodactyla OR ranidae OR corvus OR necturus OR platypus OR canaries OR bovid OR lagopus OR trimeresurus OR gariepinus OR marten OR martens OR drosophilidae OR mugil OR sunfish OR porcellus OR cypriniformes OR alouatta OR scophthalmus OR anser OR electrophorus OR putorius OR iguana OR iguanas OR lama OR lamas OR takifugu OR circus OR eptesicus OR flycatcher OR galago OR galagos OR Trachemys OR lungfish OR characiformes OR shorebird OR shorebirds OR giraffe OR giraffes OR micropterus OR scyliorhinus OR cichlidae OR loligo OR porcupine OR porcupines OR chub OR chubs OR solea OR pleuronectes OR hylidae OR viperidae OR echis OR sorex OR anchovy OR lagomorph OR ostriches OR vulture OR vultures OR whitefish OR araneus OR jird OR jirds OR tern OR esox OR drake OR drakes OR elapidae OR gallopavo OR chordata OR myodes OR caretta OR serinus OR grouse OR misgurnus OR meles OR blackbird OR blackbirds OR coregonus OR bobwhite OR bobwhites OR heteropneustes OR mammoth OR mammoths OR turdus OR rhinella OR ateles OR characidae OR clupea OR bungarus OR brill OR "Struthio camelus" ORsloth ORsloths OR pteropus OR sculpin Ranthropoids OR pollock OR pollocks OR morone OR "pan paniscus" OR litoria OR chipmunk OR chipmunks OR balaenoptera OR marmota OR melopsittacus OR hyrax OR lemming OR lemmings OR halibut OR hylobates OR lates OR caiman OR caimans OR sigmodon OR stenella OR barbel OR barbels OR sterna OR parakeet OR parakeets OR phocoena OR leptodactylus OR canidae OR buteo OR harengus OR gopher OR gophers OR marmot OR marmots OR gosling OR goslings OR platichthys OR gar OR gars OR sebastes OR marsupialia OR notophthalmus OR gazelle OR gazelles OR insectivora OR paridae OR felidae OR russula OR galliformes OR bombina OR colobus OR echidna OR echidnas OR seabass OR syncerus OR plaice OR "blue tit" OR "blue tits" OR pagrus OR catfishes OR cetacea OR barbus OR cygnus OR ficedula OR chamois OR colubridae OR perches OR coelacanth OR fitch OR urodela OR cynops OR martes OR halichoerus OR aix OR salmonidae OR leuciscus OR magpie OR magpies OR silurus OR whiting OR whitings OR Anseriformes OR colinus OR rhea OR chlorocebus OR octodon OR acinonyx OR mouflon OR mouflons OR ibex OR tetraodon OR bufonidae OR equidae OR jackal OR cephalopoda OR dendroaspis OR glama OR muskrat OR muskrats OR sable OR sables OR wildebeest OR streptopelia OR albifrons OR vespertilionidae OR woodpecker OR woodpeckers OR muntjac OR muntjacs OR archosaur OR branta OR cricetulus OR megalobrama OR poeciliidae OR desmodus OR snakehead OR snakeheads OR tench OR teal OR teals OR bandicoot OR bandicoots OR apteronotus OR phyllostomidae OR crocidura OR buzzard OR buzzards OR larimichthys OR cercocebus OR pipistrellus OR erithacus OR impala OR impalas OR rousettus OR haddock OR haddocks OR tinca ORratite OR calidris OR cynoglossus OR hypophthalmichthys OR bullock OR bullocks OR dromedaries OR alectoris OR filly OR salamandra OR cingulata OR bitis OR grus OR ammodytes OR macaw OR macaws OR hypoleuca OR sapajus OR cyprinodontiformes OR hippopotamus OR pelophylax OR capybara OR capybaras OR weasel OR weasels OR cairina OR cynomys OR lutra OR cockatoo OR cockatoos OR lachesis OR lagomorpha OR rupicapra OR daboia OR "orang utan" OR "orang utans" OR platyrrhini OR charadriiformes OR micrurus OR psittaciformes OR spalax OR loris OR mustelidae OR sylvilagus OR vitticeps OR cockatiel OR mustelus OR cottus OR erythrocebus OR dipodomys OR platessa OR callicebus OR loricariidae OR catostomus OR cuneata OR cyanistes	9846983

	OR cyprinodon OR sigmodontinae OR elasmobranchii OR Trichechus OR sauropsid OR xenarthra OR dormouse OR perissodactyla OR nautilus OR cirrhinus OR gulo OR gulos OR tragelaphus OR merula OR numida OR sciaenidae OR cerastes OR sciuridae OR gibbosus OR octopuses OR eland OR elands OR phyllomedusa OR pogona OR walrus OR agamidae OR leptodactylidae OR ridibundus OR leontopithecus OR anteater OR anteaters OR pelodiscus OR cebidae OR columbianus OR "pelteobagrus fulvidraco" OR hominoidea OR mandrillus OR "zonotrichia leucophrys" OR agama OR gobiocypris OR "bearded dragon" OR "bearded dragons" OR sarotherodon OR talpa OR discoglossus OR hagfishes OR sphenodon OR gudgeon OR amphiuma OR aythya OR tenrec OR tenrec OR hominidae OR risoria OR salamandridae OR camelidae OR columbiformes OR latimeria OR plover OR plovers OR afrotheria OR "falco sparverius" OR polecat OR polecats OR crotalinae OR salvadora OR tarsier OR lucioperca OR anchovies OR lungfishes OR terrapin OR "dromaius novaehollandiae" OR lateolabrax OR eigenmannia OR pelamis OR theropithecus OR murinae OR gander OR gymnotus OR pseudacris OR gymnophiona OR gymnotiformes OR laticauda OR falconiformes OR dugong OR dugongs OR pintail OR pintails OR rook OR rooks OR lasiurus OR catshark OR catsharks OR micropogonias OR "red junglefowl" OR paddlefish OR eutheria OR ophiophagus OR hollandicus OR nymphicus OR pimelodidae OR aepyceros OR cobitidae OR strigiformes OR cobitis OR dormice OR alytes OR calloselasma OR guanaco OR guanacos OR phasianidae OR "round goby" OR trichogaster OR catarrhini OR eelpout OR eelpouts OR galaxias OR gaur OR pungitius OR suslik OR susliks OR flatfishes OR percidae OR caprinae OR todarodes OR osmerus OR ameiurus OR anthropoidea OR "castor canadensis" OR pouting OR poutings OR tetraodontiformes OR arvicolinae OR siamang OR siamangs OR "castor fiber" OR nomascus OR "red knot" OR "red knots" OR syngnathidae OR iguanidae OR eretmochelys OR ursidae OR callimico OR columbidae OR microhylidae OR anaxyrus OR menidia OR pipistrelle OR greylag OR pipidae OR scandentia OR bowfin OR bowfins OR dendrobatidae OR zenaida OR bushbaby OR harrier OR harriers OR macropodidae OR pygerythrus OR clupeidae OR odorrana OR corvidae OR jerboa OR jerboas OR canutus OR hylobatidae OR clupeiformes OR "great cormorant" OR "great cormorants" OR scorpaeniformes OR chondrostean OR garfish OR proboscidea OR psetta OR diapsid OR serotinus OR tetrao OR walruses OR carcharhiniformes OR leucoraja OR pumpkinseed OR dosidicus OR acipenseriformes OR daubentonii OR emberizidae OR gadiformes OR hyraxes OR stizostedion OR wolverine OR wolverines OR lissotriton OR acanthurus OR centrarchidae OR gloydius OR laurasiatheria OR limosa OR psittacula OR leporidae OR proteidae OR zander OR zanders OR arapaima OR bagridae OR cyprinodontidae OR mithun OR pandion OR jackdaw OR jackdaws OR procyonidae OR carus OR jaculus OR salmoniformes OR "common sole" OR "common soles" OR protobothrops OR calamita OR brachyteles OR trionyx OR turdidae OR boidae OR luscinia OR pugnax OR euarchontoglires OR saithe OR saithes OR symphalangus OR aardvark OR aardvarks OR oystercatcher OR oystercatchers OR arius OR corydoras OR poacher OR poachers OR aurochs OR cebuella OR crecca OR lemuridae OR sirenia OR lemmus OR perdix OR glires OR lepidosaur OR muskox OR deinagkistrodon OR Pholidota OR holocephali OR cercopithecinae OR clariidae OR agapornis OR doryteuthis OR tyrannidae OR dicroglossidae OR godwit OR godwits OR monedula OR pongidae OR atheriniformes OR colobinae OR lophocebus OR atelidae OR cottidae OR leucopsis OR acanthuridae OR didelphimorphia OR elver OR elvers OR lapponica OR dermoptera OR "european hake" OR "european hakes" OR gerbillinae OR banteng OR hartebeest OR hartebeests OR hogget OR haematopus OR "anguis fragilis" OR"grey heron" OR"grey herons" OR"blue whiting" OR"blue whitings" ORfurnariidae ORmacrovipera OResocidae OR lapwing OR lapwings OR mylopharyngodon OR wallabia OR beloniformes OR potoroo OR potoroos OR "athene noctua" OR pleuronectidae OR bushbabies OR muscicapidae OR alligatoridae OR fuligula OR "bush baby" OR guineafowl OR spoonbill OR spoonbills OR viverridae OR catostomidae OR zebrafishes OR ibexes OR vendace OR estrildidae OR monotremata OR sepiella OR ambystomatidae OR shelduck OR shelducks OR treeshrew OR treeshrews OR hoplobatrachus OR pochard OR hoolock OR hoolocks OR lynxes OR antilope OR antilopes OR blackbuck OR blackbucks OR cricetinae OR paramisgurnus OR skylark OR skylarks OR soleidae OR allobates OR "northern wheatear" OR "northern wheatears" OR pitheciidae OR takin OR theria OR vanellus OR galaxiidae OR lorisidae OR ostralegus OR palaeognathae OR "stone loach" OR alauda OR callitrichinae OR caniformia OR duttaphrynus OR ictaluridae OR osteoglossiformes OR poultries OR curema OR "ruddy turnstone" OR "ruddy turnstones" OR sheatfish OR sunfishes OR centropomidae OR hemachatus OR platalea OR thamnophilidae OR "song thrush" Ratherinopsidae OR siluridae OR tadorna O Rchroicocephalus OR ermine OR ermines OR gavialis OR ruffe OR tupaiidae OR diprotodontia OR hyaenidae OR antilopinae OR crocodylidae OR herpestidae OR hippopotamidae OR "northern shoveler" OR "round gobies" OR cheirogaleidae OR indriidae OR fundulidae OR pythonidae OR rhynchocephalian OR anodorhynchus OR "red-backed shrike" OR "red-backed shrikes" OR triakidae OR phalangeridae OR aoudad OR boreoeutheria OR "eurasian jay" OR "eurasian jays" OR feliformia OR haplorhini OR osteoglossidae OR paenungulata OR struthioniformes OR ferina OR sanderling OR sanderlings OR spheniscidae OR cuttlefishes OR cygnet OR dasycneme OR gadwall OR gadwalls OR "pelobates fuscus" OR wryneck OR wrynecks OR afrosoricida OR culaea OR "dover sole" OR "dover soles" OR paralichthyidae OR passeridae OR osteolaemus OR "song thrushes" OR bluethroat OR bluethroats OR hydrophiidae OR megrim OR mephitidae OR strepsirhini OR tomistoma OR epidalea OR osmeriformes OR "bush babies" OR tarsiiform OR atelinae OR bufotes OR "eurasian coot" OR "eurasian coots" OR galagidae OR geopelia OR philomachus OR tubulidentata OR bombinatoridae OR pelobatidae OR tachysurus OR ailuridae OR woodlark OR woodlarks OR alcelaphinae OR redshank OR redshanks OR salientia OR "sand smelt" OR "sand smelts" OR woodmice OR woodmouse OR dasyproctidae OR "eurasian wigeon" OR "eurasian wigeons" OR garganey OR garganeys OR "lemon sole" OR "lemon soles" OR "common dab" OR "common dabs" OR graylag OR graylags OR leucorodia OR osphronemidae OR bewickii OR "common moorhen" OR "common moorhens" OR decapodiformes OR gobbler OR gobblers OR odontophoridae OR paddlefishes OR salmonine OR esociformes OR "eurasian woodcock" OR "eurasian woodcocks" OR "european smelt" OR "european smelts" OR goldfishes OR tenches OR tyranni OR "common chaffinch" OR "common chaffinchs" OR "common redstart" OR "common redstarts" OR "common roach" OR "common roachs" OR "great knot" OR "great knots" OR potoroidae OR alytidae OR coregonine OR dipteral OR leveret OR "poeciliopsis gracilis" OR amphiumidae OR batrachoidiformes OR "bighead goby" OR heteropneustidae OR lullula OR "norway pout" OR "norway pouts" OR sipunculida OR dogfishes OR sebastidae OR Tarsiidae OR alethinophidia OR "common nase" OR "common nases" OR "common sandpiper" OR "common sandpipers" OR "eurasian blackcap" OR "eurasian blackcaps" OR pterocnemia OR syngnathiformes OR "common chaffinches" OR eupleridae OR octopodiformes OR phascolarctidae OR scophthalmidae OR "starry smooth-hound" OR "starry smooth-hounds" OR whitefishes OR cuniculidae OR "european sprat" OR "european sprats" OR "rosy bitterling" OR "rosy bitterlings" OR "common dace" OR "common daces" OR "lesser weever" OR "lesser weevers" OR scaldfish OR "water rail" OR "water rails" OR alouattinae OR centrarchiformes OR "common whitethroat" OR "common whitethroats" OR gavialidae OR "grey gurnard" OR "grey gurnards" OR lateolabracidae OR rheiformes OR "tub gurnard" OR "tub gurnards" OR "common chiffchaff" OR "common chiffchaffs" OR garfishes OR "lesser whitethroat" OR "lesser whitethroats" OR myoxidae OR seabasses OR spariformes OR umbridae OR "yellow boxfish" OR anabantiformes OR aotidae OR "common bleak" OR "common bleaks" OR "common rudd" OR "common rudds" OR "greater pipefish" OR hapale OR nandiniidae OR "stone loaches" OR whinchat OR whinchatsOR acanthuriformes OR "brotula barbata" OR "common ling" OR "common lings" OR "common roaches" OR cottonrat OR cottonrats OR douroucoulis OR dromaiidae OR fitches OR fitchew OR galaxiiformes OR laprine OR saimiriinae OR solenette OR tarsii OR "tompot blenny" OR "common dragonet" OR "common dragonets" OR "longspined bullhead" OR "longspined bullheads" OR monotremate OR monotremates OR pempheriformes OR perdicinae OR presbytini OR smegmamorpha OR "bighead gobies" OR "carangaria incertae sedis" OR coiidae OR "fivebeard rockling" OR foulmart OR foumart OR grasskeet OR "greater pipefishes" OR ibices OR millionfish OR muguliformes OR "Norwegian topknot" OR peewit OR "red sea sailfin tang" OR rupicapras OR sheatfishes OR "tompot blennies" OR "twait shad" OR "yellow boxfishes")	
**#2**	TS=("well?being" OR "psychological restoration" OR "life NEAR/2 quality" OR "life NEAR/2 satisfaction" OR ((((mental*) OR (psychological*)) OR ("psychos*")) OR ("psychiatric*")) OR sleep* OR "insomnia" OR "post-traumatic*" OR "PTSD" OR "trauma" OR "physical /NEAR2 stress *" OR "psychological /NEAR 2stress *" OR "mental /NEAR2 stress *" OR "stress disorder*" OR "stress disease*" OR "depressi*" OR "MDD" OR "mood*" OR (("cognitive*") OR ("cognition")) OR "disorder*" OR "mania" OR "phobia" OR "anxiet*" OR "behavio* /NEAR2 disorder" OR "behavio* /NEAR 2 problem*" OR "behavio* /NEAR2 change*" OR "hyperactiv*" OR "hyper sensitive" OR ((((((((((((("grief") OR ("sorrow")) OR (("sad")) OR ("sadness"))) OR ("fear")) OR ("anger")) OR ("frustration")) OR ("guilt")) OR ("helpless*")) OR ("powerless*")) OR ("exhaustion")) OR ("lost")) OR ("lonel*")) OR ("overwhelm*")) OR ("panic*")) OR "sense of safety" OR "sense of security" OR "happiness" OR "solastalgia" OR (("adjustment problem*") OR ("learning problem*")))	4545245

**#3**	TS=("climate change*" OR "global warming" OR "climate variability*" OR "anthropogenic warming" OR "environmental change*" OR "climatic change*" OR "burning fossil fuel*" OR "atmospheric warm*" OR "increased humidity" OR "climate hazard*" OR "climate crisis" OR "rising temperature*" OR "precipitation change*" OR "sea level rise" OR "environmental impact*" OR "environmental disaster*" OR "climate adaptation*" OR "albedo change*" OR "rising sea levels" OR "earth warming" OR "temperature change*" OR "environmental amenit*" OR "environmental dynamic*" OR "increased CO2 emission*" OR "natural disasters" OR "carbon dioxide emission*" OR "methane emission*" OR "CH4 emission*" OR "hot climate*" OR "NO2 emission*" OR "nitrogen dioxide emission*" OR "warming planet" OR "north Atlantic oscillation*" OR "NAO" OR "humidity change*" OR "increased radiative forcing" OR " radiative forcing scenario*" OR "dirty fuel*" OR "atmospheric blocking" OR "fossil fuel combustion*" OR "urban heat island" OR "UHI" OR "environmental stressor" OR "heat wave*" OR "cold wave*" OR "drought*" OR "flood*" OR ((("wildfire*") OR ("bushfire*")) OR ("forest fire*")) OR "heavy precipitation" OR "extreme weather event*" OR "dust storm*" OR "dry period*" OR "extreme meteorological event*" OR "allergen" OR "smog" OR "sand storm*" OR "heavy snow" OR "extreme heat event*" OR "aridity" OR<br>"air pollution*" OR "Ozone" OR "particulate matter" OR "PM" OR "PM2.5" OR "ozone*")	1242935
**#4**	TS=("Europ*" OR" EU" OR "European Union" OR "continental Europe" OR "European region*" OR "European countr*")	1114553
**#5**	TS=("surrounding*" OR "natural space*" OR "natural environment" OR "park*" OR<br>"green*" OR "vegetation" OR" forest*" OR "woodland*" OR "meadow*" OR "grassland" OR "shrubland" OR "crop*" OR "agricultural area*" OR "cultivated land*" OR "land use mix" OR<br>"blue*" OR "wetland*" OR (("water body") OR ("water bodies")) OR "pond*" OR "lake*" OR "beach*" OR "coastal water*" OR "pool" OR "inland-blue space" OR "river*" OR canal* OR "coastal-blue space*" OR "esplanade*" OR<br>(("built environment*") OR ("built?up area*")) OR "grey space*" OR "street*" OR<br>"residential area*" OR ((("housing NEAR/2 area*") OR ("neighbo?rhood*")) OR ("living NEAR/2 area*")) OR "livestock*" OR "waste dump*")	3892675
**#6**	TS=("education*" OR "learning")	2141716
**#7**	TS=("living situation" OR "housing situation" OR "ownership right*" OR "ownership status" OR "house" OR "household*" OR "living NEAR/2 space" OR<br>(("air condition*") OR ("air cooling")) OR "heating" OR "ventilation")	919920
**#8**	TS=("exercise*" OR "sport*" OR "playing" OR" hobb*" OR "physical* activ*" OR "leisure" OR "leisure time activity" OR "recreation*")	981893
**#9**	TS=("employ*" OR "unemploy*" OR "under?employment" OR "work contract*" OR "retire*" OR "profession*" OR "labo?r*" OR "occupation*" OR "work alienation" OR "outdoor worker*" OR "commuter*" OR "office worker*")	2607757
**#10**	TS=("cycling" OR "running" OR ((("active transport*") OR ("public transport")) OR ("passive transport")) OR "travel*" OR "active movement*" OR "walking" OR "travel NEAR/2 time" OR "choice of transportation" OR (("independent mobility") OR ("independent travel")))	739492
**#11**	TS=("goods" OR "subsistence*" OR "consumption*")	907331
**#12**	TS=("income" OR "salary NEAR/2 level" OR "financial*" OR "economic strains" OR "livelihood*")	663733
**#13**	TS=("civil status" OR "marital status" OR "societ*" OR "communit*" OR "support*" OR "social*" OR "famil*" OR "lifestyle" OR "isolation" OR "sense of loneliness" OR ((("parental anxiety*") OR ("parental stress")) OR ("parental depression*")))	8645089
**#14**	TS=((("genetic*") OR ("inherited*")) OR "motivation*" OR "illness*" OR "awareness" OR "allerg*" OR "breastfeed*" OR "formula feeding" OR "belief*" OR "age" OR (("sex") OR ("gender")) OR (("prematurity") OR ("birth weight")) OR "environmental value*" OR "environmental concern*" OR "environmental sensitivity" OR "connectedness with nature")	6425027
**#15**	TS=("socio-economic status" OR "SES")	41318
**#16**	#15 OR #14 OR #13 OR #12 OR #11 OR #10 OR #9 OR #8 OR #7 OR #6 OR #5	20286192
**#17**	#2 AND #3 AND #4	2134
**#18**	#16 AND #17	1758
**#19**	#18 Timespan: 2011-01-01 to 2023-01-31	1297
**#20**	#19 not #1 Timespan: 2011-01-01 to 2023-01-31	1078
**#21**	#19 not #1 and English (Languages) Timespan: 2011-01-01 to 2023-01-31	1041
**#22**	#21 not ALL=(("systematic review") or ("review"))	871
**#23**	#22 not ALL=(("RCT") or ("random?ed control trail")) Timespan: 2011-01-01 to 2023-01-31	870
	Scopus	
**#4**	TITLE-ABS-KEY (rat OR rats OR animal OR animals OR mice OR "in vivo" OR mouse OR rabbit OR rabbits OR murine OR pig OR pigs OR dog OR dogs OR bovine OR fish OR vertebrate OR vertebrates OR cat OR cats OR rodent OR rodents OR mammal OR mammals OR chicken OR chickens OR monkey OR monkeys OR sheep OR canine OR canines OR porcine OR cattle OR bird OR birds OR hamster OR hamsters OR primate OR primates OR cow OR cows OR chick OR horse OR horses OR avian OR avians OR calf OR swine OR swines OR xenopus OR turkeys OR bear OR bears OR frog OR frogs OR zebrafish OR goat OR goats OR equine OR calves OR poultry OR macaque OR macaques OR mole OR moles OR ovine OR lamb OR lambs OR fishes OR diptera OR amphibian OR amphibians OR snake OR snakes OR ruminant OR ruminants OR hen OR hens OR piglet OR piglets OR feline OR felines OR simian OR simians OR laevis OR trout OR trouts OR teleost OR teleosts OR salmon OR salmons OR seal OR seals OR bull OR bulls OR ewe OR ewes OR hedgehog OR hedgehogs OR macaca OR macacas OR proteus OR pigeon OR pigeons OR bat OR bats OR duck OR ducks OR chimpanzee OR chimpanzees OR baboon OR baboons OR deer OR rana OR ranas OR carp OR carps OR heifer OR swallow OR swallows OR lizard OR lizards OR canis OR sow OR sows OR cynomolgus OR quail OR quails OR reptile OR reptiles OR turtle OR turtles OR buffalo OR gerbil OR gerbils OR boar OR boars OR squirrel OR squirrels OR oncorhynchus OR mus OR toad OR toads OR fowl OR fowls OR rerio OR danio OR ara OR aras OR musculus OR tadpole OR tadpoles OR mulatta OR salmo OR ram OR eagle OR eagles OR ferret OR ferrets OR goldfish OR catfish OR whale OR whales OR fox OR foxes OR ape OR apes OR elephant OR elephants OR bos OR marmoset OR marmosets OR cod OR cods OR shark OR sharks OR wolf OR eel OR eels OR auratus OR rattus OR zebra OR zebras OR tilapia OR tilapias OR gilt OR camel OR camels OR squid OR gallus OR marsupial OR marsupials OR vole OR voles OR fascicularis OR ovis OR salmonid OR salmonids OR tiger OR tigers OR dolphin OR dolphins OR robin OR robins OR carpio OR opossum OR opossums OR cyprinus OR salamander OR salamanders OR felis OR mink OR minks OR swan OR swans OR norvegicus OR bufo OR torpedo OR bass OR lamprey OR lampreys OR sus OR python OR pythons OR tetrapod OR tetrapods OR shrew OR shrews OR lion OR lions OR hog OR hogs OR songbird OR songbirds OR oreochromis OR starling OR starlings OR caprine OR carassius OR owl OR owls OR newt OR newts OR papio OR scrofa OR hare OR hares OR gorilla OR gorillas OR flounder OR flounders OR goose OR herring OR herrings OR therian OR buffaloes OR canary OR sparrow OR sparrows OR microtus OR octopus OR troglodytes OR tuna OR amphibia OR chinchilla OR chinchillas OR ide OR oryzias OR cervus OR kangaroo OR kangaroos OR armadillo OR armadillos OR callithrix OR "pan troglodytes" OR saimiri OR cichlid OR cichlids OR donkey OR donkeys OR bream OR char OR chars OR finch OR raccoon OR raccoons OR bothrops OR anguilla OR perch OR cricetus OR seabird OR seabirds OR buck OR bucks OR naja OR coturnix OR salmonids OR geese OR minnow OR minnows OR raptor OR raptors OR merione OR meriones OR rodentia OR elaphus OR amniote OR amniotes OR elasmobranch OR emu OR emus OR peromyscus OR hominid OR hominids OR bubalus OR crotalus OR gull OR gulls OR anas OR anura OR lemur OR lemurs OR crow OR crows OR camelus OR gibbon OR gibbons OR waterfowl OR parrot OR parrots OR eels OR cob OR stickleback OR sticklebacks OR columba OR mesocricetus OR ambystoma OR raven OR ravens OR gadus OR penguin OR penguins OR orangutan OR orangutans OR sturgeon OR sturgeons OR cuniculus OR aves OR virginianus OR cephalopod OR cephalopods OR cebus OR sparus OR tortoise OR tortoises OR guttata OR morhua OR unguiculatus OR dogfish OR vulpes OR mallard OR mallards OR apodemus OR alligator OR alligators OR oryctolagus OR llama OR llamas OR reindeer OR Mustela OR duckling OR ducklings OR wolves OR sander OR amazona OR zebu OR badger OR badgers OR dove OR doves OR ictalurus OR capra OR capras OR	32

	equus OR camelid OR camelids OR poecilia OR mule OR mules OR perciformes OR salvelinus OR labrax OR cyprinidae OR ariidae OR crocodile OR crocodiles OR fundulus OR dicentrarchus OR clarias OR cercopithecus OR chiroptera OR alpaca OR alpacas OR pike ORpikes OR paralichthys OR puma OR pumas OR didelphis OR pisces OR Macropus OR triturus OR bison OR bisons OR epinephelus OR gasterosteus OR panthera OR acipenser OR mackerel OR mackerels OR tamarin OR tamarins OR ostrich OR anolis OR vervet OR vervets OR wallaby OR glareolus OR beaver OR beavers OR dromedary OR catus OR killifish OR pimephales OR promelas OR aotus OR phoca OR panda OR pandas OR porpoise OR porpoises OR myotis OR yak OR yaks OR agkistrodon OR vipera OR otter OR otters OR turbot OR turbots OR squamate OR carnivora OR mullet OR mulletsOR hawk OR hawks OR taeniopygia OR seahorse OR seahorses OR "poecilia reticulata" OR falcon OR falcons OR prosimian OR prosimians OR parus OR perca OR fingerling OR fingerlings OR antelope OR antelopes OR tupaia OR passeriformes OR sepia OR saguinus OR coyote OR coyotes OR pongo OR meleagris OR reptilia OR lepus OR psittacine OR hagfish OR warbler OR warblers OR "russell s viper" OR "russell s vipers" OR smolt OR smolts OR budgerigar OR sardine OR sardines OR cavia OR cavias OR hyla OR pleurodeles OR siluriformes OR "great tit" OR "great tits" OR guppy OR bonobo OR bonobos OR rutilus OR trichosurus OR muridae OR phodopus OR channa OR squalus OR lynx OR sturnus OR petromyzon OR vitulina OR monodelphis OR cuttlefish OR adder OR adders OR lepomis OR canaria OR gambusia ORguppies OR xiphophorus OR flatfish OR koala OR koalas OR labeo OR stingray OR stingrays OR chelonia OR lampetra OR spermophilus OR crocodilian OR "passer domesticus" OR sciurus OR artiodactyla OR ranidae OR corvus OR necturus OR platypus OR canaries OR bovid OR lagopus OR trimeresurus OR gariepinus OR marten OR martens OR drosophilidae OR mugil OR sunfish OR porcellus OR cypriniformes OR alouatta OR scophthalmus OR anser OR electrophorus OR putorius OR iguana OR iguanas OR lama OR lamas OR takifugu OR circus OR eptesicus OR flycatcher OR galago OR galagos OR Trachemys OR lungfish OR characiformes OR shorebird OR shorebirds OR giraffe OR giraffes OR micropterus OR scyliorhinus OR cichlidae OR loligo OR porcupine OR porcupines OR chub OR chubs OR solea OR pleuronectes OR hylidae OR viperidae OR echis OR sorex OR anchovy OR lagomorph OR ostriches OR vulture OR vultures OR whitefish OR araneus OR jird OR jirds OR tern OR esox OR drake OR drakes OR elapidae OR gallopavo OR chordata OR myodes OR caretta OR serinus OR grouse OR misgurnus OR meles OR blackbird OR blackbirds OR coregonus OR bobwhite OR bobwhites OR heteropneustes OR mammoth OR mammoths OR turdus OR rhinella OR ateles OR characidae OR clupea OR bungarus OR brill OR "Struthio camelus" OR sloth OR sloths OR pteropus OR sculpin Ranthropoids OR pollock)	
**#7**	TITLE-ABS-KEY (pollocks OR morone OR "pan paniscus" OR litoria OR chipmunk OR chipmunks OR balaenoptera OR marmota OR melopsittacus OR hyrax OR lemming OR lemmings OR halibut OR hylobates OR lates OR caiman OR caimans OR sigmodon OR stenella OR barbel OR barbels OR sterna OR parakeet OR parakeets OR phocoena OR leptodactylus OR canidae OR buteo OR harengus OR gopher OR gophers OR marmot OR marmots OR gosling OR goslings OR platichthys OR gar OR gars OR sebastes OR marsupialia OR notophthalmus OR gazelle OR gazelles OR insectivora OR paridae OR felidae OR russula OR galliformes OR bombina OR colobus OR echidna OR echidnas OR seabass OR syncerus OR plaice OR "blue tit" OR "blue tits" OR pagrus OR catfishes OR cetacea OR barbus OR cygnus OR ficedula OR chamois OR colubridae OR perches OR coelacanth OR fitch OR urodela OR cynops OR martes OR halichoerus OR aix OR salmonidae OR leuciscus OR magpie OR magpies OR silurus OR whiting OR whitings OR Anseriformes OR colinus OR rhea OR chlorocebus OR octodon OR acinonyx OR mouflon OR mouflons OR ibex OR tetraodon OR bufonidaeOR equidae OR jackal OR cephalopoda OR dendroaspis OR glama OR muskrat OR muskrats OR sable OR sables OR wildebeest OR streptopelia OR albifrons OR vespertilionidae OR woodpecker OR woodpeckers OR muntjac OR muntjacs OR archosaur OR branta OR cricetulus OR megalobrama OR poeciliidae OR desmodus OR snakehead OR snakeheads OR tench OR teal OR teals OR bandicoot OR bandicoots OR apteronotus OR phyllostomidae OR crocidura OR buzzard OR buzzards OR larimichthys OR cercocebus OR pipistrellus OR erithacus OR impala OR impalas OR rousettus OR haddock OR haddocks OR tinca OR ratite OR calidris OR cynoglossus OR hypophthalmichthys OR bullock OR bullocks OR dromedaries OR alectoris OR filly OR salamandra OR cingulata OR bitis OR grus OR ammodytes OR macaw OR macaws OR hypoleuca OR sapajus OR cyprinodontiformes OR hippopotamus OR pelophylax OR capybara OR capybaras OR weasel OR weasels OR cairina OR cynomys OR lutra OR cockatoo OR cockatoos OR lachesis OR lagomorpha OR rupicapra OR daboia OR "orang utan" OR "orang utans" OR platyrrhini OR charadriiformes OR micrurus OR psittaciformes OR spalax OR loris OR mustelidae OR sylvilagus OR vitticeps OR cockatiel OR mustelus OR cottus OR erythrocebus OR dipodomys OR platessa OR callicebus OR loricariidae OR catostomus OR cuneata OR cyanistes OR cyprinodon OR sigmodontinae OR elasmobranchii OR Trichechus OR sauropsid OR xenarthra OR dormouse OR perissodactyla OR nautilus OR cirrhinus OR gulo OR gulos OR tragelaphus OR merula OR numida OR sciaenidae OR cerastes OR sciuridae OR gibbosus OR octopuses OR eland OR elands OR phyllomedusa OR pogona OR walrus OR agamidae OR leptodactylidae OR ridibundus OR leontopithecus OR anteater ORanteaters OR pelodiscus OR cebidae OR columbianus OR "pelteobagrus fulvidraco" OR hominoidea OR mandrillus OR "zonotrichia leucophrys" OR agama OR gobiocypris OR "bearded dragon" OR "bearded dragons" OR sarotherodon OR talpa OR discoglossus OR hagfishes OR sphenodon OR gudgeon OR amphiuma OR aythya OR tenrec OR tenrec OR hominidae OR risoria OR salamandridae OR camelidae OR columbiformes OR latimeria OR plover OR plovers OR afrotheria OR "falco sparverius" OR polecat OR polecats OR crotalinae OR salvadora OR tarsier OR lucioperca OR anchovies OR lungfishes OR terrapin OR "dromaius novaehollandiae" OR lateolabrax OR eigenmannia OR pelamis OR theropithecus OR murinae OR gander OR gymnotus OR pseudacris OR gymnophiona OR gymnotiformes OR laticauda OR falconiformes OR dugong OR dugongs OR pintail OR pintails OR rook OR rooks OR lasiurus OR catshark OR catsharks OR micropogonias OR "redjunglefowl" OR paddlefish OR eutheria OR ophiophagus OR hollandicus OR nymphicus OR pimelodidae OR aepyceros OR cobitidae OR strigiformes OR cobitis OR dormice OR alytes OR calloselasma OR guanaco OR guanacos OR phasianidae OR "round goby" OR trichogaster OR catarrhini OR eelpout OR eelpouts OR galaxias OR gaur OR pungitius OR suslik OR susliks OR flatfishes OR percidae OR caprinae OR todarodes OR osmerus OR ameiurus OR anthropoidea OR "castor canadensis" OR pouting OR poutings OR tetraodontiformes OR arvicolinae OR siamang OR siamangs OR "castor fiber" OR nomascus OR "red knot" OR "red knots" OR syngnathidae OR iguanidae OR eretmochelys OR ursidae OR callimico OR columbidae OR microhylidae OR anaxyrus OR menidia OR pipistrelle OR greylag OR pipidae OR scandentia OR bowfin OR bowfins ORdendrobatidae OR zenaida OR bushbaby OR harrier OR harriers OR macropodidae OR pygerythrus OR clupeidae OR odorrana OR corvidae OR jerboa OR jerboas OR canutus OR hylobatidae OR clupeiformes OR "great cormorant" OR "great cormorants" OR scorpaeniformes OR chondrostean OR garfish OR proboscidea OR psetta OR diapsid OR serotinus OR tetrao OR walruses OR carcharhiniformes OR leucoraja OR pumpkinseed OR dosidicus OR acipenseriformes OR daubentonii OR emberizidae OR gadiformes OR hyraxes OR stizostedion OR wolverine OR wolverines OR lissotriton OR acanthurus OR centrarchidae OR gloydius OR laurasiatheria OR limosa OR psittacula OR leporidae OR proteidae OR zander OR zanders OR arapaima OR bagridae OR cyprinodontidae OR mithun OR pandion OR jackdaw OR jackdaws OR procyonidae OR carus ORjaculus OR salmoniformes OR "common sole" OR "common soles" OR protobothrops OR calamita OR brachyteles OR trionyx OR turdidae OR boidae OR luscinia OR pugnax OR euarchontoglires OR saithe OR saithes OR symphalangus OR aardvark OR aardvarks OR oystercatcher OR oystercatchers OR arius OR corydoras OR poacher OR poachers OR aurochs OR cebuella OR crecca OR lemuridae OR sirenia OR lemmus OR perdix OR glires OR lepidosaur OR muskox OR deinagkistrodon OR Pholidota OR holocephali OR cercopithecinae OR clariidae OR agapornis OR doryteuthis OR tyrannidae OR dicroglossidae OR godwitOR godwits OR monedula OR pongidae OR atheriniformes OR colobinae OR lophocebus OR atelidae OR cottidae OR leucopsis OR acanthuridae OR didelphimorphia OR elver OR elvers OR lapponica OR dermoptera OR "european hake" OR "european hakes" OR gerbillinae OR banteng OR hartebeest OR hartebeests OR hogget OR haematopus OR "anguis fragilis" OR "grey heron" OR "grey herons" OR "blue whiting" OR "blue whitings" OR furnariidae OR macrovipera OR esocidae OR lapwing OR lapwings OR mylopharyngodon OR wallabia OR beloniformes OR potoroo OR potoroos OR "athene noctua" OR pleuronectidae OR bushbabies OR muscicapidae OR alligatoridae OR fuligula OR "bush baby" OR guineafowl OR spoonbill OR spoonbills OR viverridae OR catostomidae OR zebrafishes OR ibexes OR vendace OR estrildidae OR monotremata ORsepiella OR ambystomatidae OR shelduck OR shelducks OR treeshrew OR treeshrews OR hoplobatrachus OR pochard OR hoolock OR hoolocks OR lynxes OR antilope OR antilopes OR blackbuck OR blackbucks)	4
**#9**	TITLE-ABS-KEY (cricetinae OR paramisgurnus OR skylark OR skylarks OR soleidae OR allobates OR "northern wheatear" OR "northern wheatears" OR pitheciidae OR takin OR theria OR vanellus OR galaxiidae OR lorisidae OR ostralegus OR palaeognathae OR "stone loach"	1822

	OR alauda OR callitrichinae OR caniformia OR duttaphrynus OR ictaluridae OR osteoglossiformes OR poultries OR curema OR "ruddy turnstone" OR "ruddy turnstones" OR sheatfish OR sunfishes OR centropomidae OR hemachatus OR platalea OR thamnophilidae OR "song thrush" OR Ratherinopsidae OR siluridae OR tadorna OR chroicocephalus OR ermine OR ermines OR gavialis OR ruffe OR tupaiidae OR diprotodontia OR hyaenidae OR antilopinae OR crocodylidae OR herpestidae OR hippopotamidae OR "northern shoveler" OR "round gobies" OR cheirogaleidae OR indriidae OR fundulidae OR pythonidae OR rhynchocephalian OR anodorhynchus OR "red-backed shrike" OR "red-backed shrikes" OR triakidae OR phalangeridae OR aoudad OR boreoeutheria OR "eurasian jay" OR "eurasian jays" OR feliformia OR haplorhini OR osteoglossidae OR paenungulata OR struthioniformes OR ferina OR sanderling OR sanderlings OR spheniscidae OR cuttlefishes OR cygnet OR dasycneme OR gadwall OR gadwalls OR "pelobates fuscus" OR wryneck OR wrynecks OR afrosoricida OR culaea OR "dover sole" OR "dover soles" OR paralichthyidae OR passeridae OR osteolaemus OR "song thrushes" OR bluethroat OR bluethroats OR hydrophiidae OR megrim OR mephitidae OR strepsirhini OR tomistoma OR epidalea OR osmeriformes OR "bush babies" OR tarsiiform OR atelinae OR bufotes OR "eurasian coot" OR "eurasian coots" OR galagidae OR geopelia OR philomachus OR tubulidentata OR bombinatoridae OR pelobatidae OR tachysurus OR ailuridae OR woodlark OR woodlarks OR alcelaphinae OR redshank OR redshanks OR salientia OR "sand smelt" OR "sand smelts" OR woodmice OR woodmouse OR dasyproctidae OR "eurasian wigeon" OR "eurasian wigeons" OR garganey OR garganeys OR "lemon sole" OR "lemon soles" OR "common dab" OR "common dabs" OR graylag OR graylags OR leucorodia OR osphronemidae OR bewickii OR "common moorhen" OR "common moorhens" OR decapodiformes OR gobbler OR gobblers OR odontophoridae OR paddlefishes OR salmonine OR esociformes OR "eurasian woodcock" OR "eurasian woodcocks" OR "european smelt" OR "european smelts" OR goldfishesOR tenches OR tyranni OR "common chaffinch" OR "common chaffinchs" OR "common redstart" OR "common redstarts" OR "common roach" OR "common roachs" OR "great knot" OR "great knots" OR potoroidae OR alytidae OR coregonine OR dipteral OR leveret OR "poeciliopsis gracilis" OR amphiumidae OR batrachoidiformes OR "bighead goby" OR heteropneustidae OR lullula OR "norway pout" OR "norway pouts" OR sipunculida OR dogfishes OR sebastidae OR Tarsiidae OR alethinophidia OR "common nase" OR "common nases" OR "common sandpiper" OR "common sandpipers" OR "eurasian blackcap" OR "eurasian blackcaps" OR pterocnemia OR syngnathiformes OR "common chaffinches" OR eupleridae OR octopodiformes OR phascolarctidae OR scophthalmidae OR "starry smooth-hound" OR "starry smooth-hounds" OR whitefishes OR cuniculidae OR "european sprat" OR "european sprats" OR "rosy bitterling" OR "rosy bitterlings" OR "common dace" OR "common daces" OR "lesser weever" OR "lesser weevers" OR scaldfish OR "water rail" OR "water rails" OR alouattinae OR centrarchiformes OR "common whitethroat" OR "common whitethroats" OR gavialidae OR "grey gurnard" OR "grey gurnards" OR lateolabracidae OR rheiformes OR "tub gurnard" OR "tub gurnards" OR "common chiffchaff" OR "common chiffchaffs" OR garfishes OR "lesser whitethroat" OR "lesser whitethroats" OR myoxidae OR seabasses OR spariformes OR umbridae OR "yellow boxfish" OR anabantiformes OR aotidae OR "common bleak" OR "common bleaks" OR "common rudd" OR "common rudds" OR "greater pipefish" OR hapale OR nandiniidae OR "stone loaches" OR whinchat OR whinchats OR acanthuriformes OR "brotula barbata" OR "common ling" OR "common lings" OR "common roaches" OR cottonrat OR cottonrats OR douroucoulis OR dromaiidae OR fitches OR fitchew OR galaxiiformes OR laprine OR saimiriinae OR solenette OR tarsii OR "tompot blenny" OR "common dragonet" OR "common dragonets" OR "longspined bullhead" OR "longspined bullheads" OR monotremate OR monotremates OR pempheriformes OR perdicinae OR presbytini OR smegmamorpha OR "bighead gobies" OR "carangaria incertae sedis" OR coiidae OR "fivebeard rockling" OR foulmart OR foumart OR grasskeet OR "greater pipefishes" OR ibices OR millionfish OR muguliformes OR "Norwegian topknot" OR peewit OR "red sea sailfin tang" OR rupicapras OR sheatfishes OR "tompot blennies" OR "twait shad" OR "yellow boxfishes")	
**#10**	#4 OR #7 OR #9	1857
**#12**	TITLE-ABS-KEY ("well?being" OR "psychological restoration" OR "mental restoration" OR "life W/2 quality" OR "life W/2 satisfaction" OR ("mental*" OR "psychological*" OR "psychos*" OR "psychiatric*") OR "sleep*" OR "insomnia" OR "post-traumatic*" OR "PTSD" OR "trauma" OR "physical W/2 stress*" OR "psychological W/2 stress*" OR "mental adj2 stress*" OR "stress disorder*" OR "stress disease*" OR "depressi*" OR "MDD" OR "mood*" OR ("cognitive*" OR "cognition") OR "disorder*" OR "mania" OR "phobia" OR "anxiet*" OR "behavio* W/2 disorder" OR "behavio* W/2 problem*" OR "behavio* W/2 change*" OR "hyperactiv*" OR "hyper sensitive" OR ("grief" OR "sorrow" OR "sad" OR "sadness" OR "fear" OR "anger" OR "frustration" OR "guilt" OR "helpless*" OR "powerless*" OR "exhaustion" OR "lost" OR "lonel*" OR "overwhelm*" OR "panic*") OR "sense of safety" OR "sense of security" OR "happiness" OR "solastalgia" OR ("adjustment problem*" OR "learning problem*"))	8592365
**#13**	TITLE-ABS-KEY ("climate change*" or "global warming" or "climate variability*" or "anthropogenic warming" or "environmental change*" or "climatic change*" or "burning fossil fuel*" or "atmospheric warm*" or "increased humidity" or "climate hazard*" or "climate crisis" or "rising temperature*" or "precipitation change*" or "sea level rise" or "environmental impact*" or "environmental disaster*" or "climate adaptation*" or "albedo change*" or "rising sea levels" or "earth warming" or "temperature change*" or "environmental amenit*" or "environmental dynamic*" or "increased CO2 emission*" or "carbon dioxide emission*" or "methane emission*" or "CH4 emission*" or "hot climate*" or "NO2 emission*" or "nitrogen dioxide emission*" or "warming planet" or "north Atlantic oscillation*" or "NAO" or "humidity change*" or "increased radiative forcing" or "radiative forcing scenario*" or "dirty fuel*" or "atmospheric blocking" or "fossil fuel combustion*" or "urban heat island" or "UHI" or "environmental stressor" or "heat wave*" or "cold wave*" or "drought*" or "flood*" or ("wildfire*" or "bushfire*" or "forest fire*") or "heavy precipitation" or "extreme weather event*" or "dust storm*" or "dry period*" or "extreme meteorological event*" or "allergen" or "smog" or "sand storm*" or "heavy snow" or "extreme heat event*" or "aridity" or "air pollution*" or "Ozone" or "particulate matter" or "PM" or "PM2.5" or "ozone*" or "natural disasters" or "particulate matter")	2441595
**#14**	TITLE-ABS-KEY ("Europ*" or "EU" or "European Union" or "continental Europe" or "European region*" or "European countr*")	2032409
**#15**	TITLE-ABS-KEY ("surrounding*" or "natural space*" or "natural environment" or "park*" or "green*" or "vegetation" or "forest*" or "woodland*" or "meadow*" or "grassland" or "shrubland" or "crop*" or "agricultural area*" or "cultivated land*" or "land use mix" or "blue*" or "wetland*" or ("water body" or "water bodies") or "pond*" or "lake*" or "beach*" or "coastal water*" or "pool" or "inland-blue space" or "river*" or "canal*" or "coastal-blue space*" or "esplanade*" or ("built environment*" or "built?up area*") or "grey space*" or "street*" or "residential area*" or ("housing W/2 area*" or "neighbo?rhood*" or "living W/2 area*") or "livestock*" or "waste dump*")	6821609
**#16**	TITLE-ABS-KEY ("education*" or "learning")	6036162
**#17**	TITLE-ABS-KEY ("living situation" or "housing situation" or "ownership right*" or "ownership status" or "house*" or "household*" or "living W/2 space" or ("air condition*" or "air cooling") or "heating" or "ventilation")	2051821
**#18**	TITLE-ABS-KEY ("exercise*" or "sport*" or "playing" or " hobb*" or "physical* activ*" or "leisure" or "leisure time activity" or "recreation*")	1802076
**#19**	TITLE-ABS-KEY ("employ*" or "unemploy*" or "under?employment" or "work contract*" or "retire*" or "profession*" or "labo?r*" or "occupation*" or "work alienation" or "outdoor worker*" or "commuter*" or "office worker*")	6286249
**#20**	TITLE-ABS-KEY ("Cycling" or "running" or ("active transport*" or "public transport" or "passive transport") or "travel*" or "active movement*" or "walking" or "travel W/2 time" or "choice of transportation" or ("independent mobility" or "independent travel")	1525081
**#21**	TITLE-ABS-KEY ("goods" or "subsistence*" or "consumption*")	1939618
**#22**	TITLE-ABS-KEY ("Income" or "salary W/2 level" or "financial*" or "economic strains" or "unemployment adj2 rate*" or "livelihood*")	1466715
**#23**	TITLE-ABS-KEY ("civil status" or "marital status" or "societ*" or "communit*" or "support*" or "social*" or "famil*" or "lifestyle" or "isolation" or "sense of loneliness" or ("parental anxiety*" or "parental stress" or "parental depression*"))	20189528
**#24**	TITLE-ABS-KEY (genetic* or "inherited*" or motivation* or illness* or awareness or "allerg*" or "breastfeed*" or "formula feeding" or "belief*" or "age" or ("sex" or "gender") or ("prematurity" or "birth weight") or "environmental value*" or "environmental concern*" or "environmental sensitivity" or "connectedness with nature")	14295903
**#25**	TITLE-ABS-KEY ("socio-economic status" or "SES")	76134
**#26**	#15 OR #16 OR #17 OR #18 OR #19 OR #20 OR #21 OR #22 OR #23 OR #24 OR #25	43869468

	(TITLE-ABS-KEY ("surrounding*" or "natural space*" or "natural environment" or "park*" or "green*" or "vegetation" or "forest*" or "woodland*" or "meadow*" or "grassland" or "shrubland" or "crop*" or "agricultural area*" or "cultivated land*" or "land use mix" or "blue*" or "wetland*" or ("water body" or "water bodies") or "pond*" or "lake*" or "beach*" or "coastal water*" or "pool" or "inland-blue space" or "river*" or "canal*" or "coastal-blue space*" or "esplanade*" or ("built environment*" or "built?up area*") or "grey space*" or "street*" or "residential area*" or ("housing W/2 area*" or "neighbo?rhood*" or "living W/2 area*") or "livestock*" or "waste dump*")) OR (TITLE-ABS-KEY ("education*" or "learning")) OR (TITLE-ABS-KEY ("living situation" or "housing situation" or "ownership right*" or "ownership status" or "house*" or "household*" or "living W/2 space" or ("air condition*" or "air cooling") or "heating" or "ventilation")) OR (TITLE-ABS-KEY ("exercise*" or "sport*" or "playing" or " hobb*" or "physical* activ*" or "leisure" or "leisure time activity" or "recreation*")) OR (TITLE-ABS-KEY ("employ*" or "unemploy*" or "under?employment" or "work contract*" or "retire*" or "profession*" or "labo?r*" or "occupation*" or "work alienation" or "outdoor worker*" or "commuter*" or "office worker*")) OR (TITLE-ABS-KEY ("Cycling" or "running" or ("active transport*" or "public transport" or "passive transport") or "travel*" or "active movement*" or "walking" or "travel W/2 time" or "choice of transportation" or ("independent mobility" or "independent travel"))) OR (TITLE-ABS-KEY ("goods" or "subsistence*" or "consumption*")) OR (TITLE-ABS-KEY ("Income" or "salary W/2 level" or "financial*" or "economic strains" or "unemployment adj2 rate*" or "livelihood*")) OR (TITLE-ABS-KEY ("civil status" or "marital status" or "societ*" or "communit*" or "support*" or "social*" or "famil*" or "lifestyle" or "isolation" or "sense of loneliness" or ("parental anxiety*" or "parental stress" or "parental depression*"))) OR (TITLE-ABS-KEY (genetic* or "inherited*" or motivation* or illness* or awareness or "allerg*" or "breastfeed*" or "formula feeding" or "belief*" or "age" or ("sex" or "gender") or ("prematurity" or "birth weight") or "environmental value*" or "environmental concern*" or "environmental sensitivity" or "connectedness with nature")) OR (TITLE-ABS-KEY ("socio-economic status" or "SES"))	
**#27**	#12 AND #13 AND #14<br>(TITLE-ABS-KEY ("well?being" OR "psychological restoration" OR "mental restoration" OR "life W/2 quality" OR "life W/2 satisfaction" OR ("mental*" OR "psychological*" OR "psychos*" OR "psychiatric*") OR "sleep*" OR "insomnia" OR "post-traumatic*" OR "PTSD" OR "trauma" OR "physical W/2 stress*" OR "psychological W/2 stress*" OR "mental adj2 stress*" OR "stress disorder*" OR "stress disease*" OR "depressi*" OR "MDD" OR "mood*" OR ("cognitive*" OR "cognition") OR "disorder*" OR "mania" OR "phobia" OR "anxiet*" OR "behavio* W/2 disorder" OR "behavio* W/2 problem*" OR "behavio* W/2 change*" OR "hyperactiv*" OR "hyper sensitive" OR ("grief" OR "sorrow" OR "sad" OR "sadness" OR "fear" OR "anger" OR "frustration" OR "guilt" OR "helpless*" OR "powerless*" OR "exhaustion" OR "lost" OR "lonel*" OR "overwhelm*" OR "panic*") OR "sense of safety" OR "sense of security" OR "happiness" OR "solastalgia" OR ("adjustment problem*" OR "learning problem*"))) AND (TITLE-ABS-KEY ("climate change*" or "global warming" or "climate variability*" or "anthropogenic warming" or "environmental change*" or "climatic change*" or "burning fossil fuel*" or "atmospheric warm*" or "increased humidity" or "climate hazard*" or "climate crisis" or "rising temperature*" or "precipitation change*" or "sea level rise" or "environmental impact*" or "environmental disaster*" or "climate adaptation*" or "albedo change*" or "rising sea levels" or "earth warming" or "temperature change*" or "environmental amenit*" or "environmental dynamic*" or "increased CO2 emission*" or "carbon dioxide emission*" or "methane emission*" or "CH4 emission*" or "hot climate*" or "NO2 emission*" or "nitrogen dioxide emission*" or "warming planet" or "north Atlantic oscillation*" or "NAO" or "humidity change*" or "increased radiative forcing" or "radiative forcing scenario*" or "dirty fuel*" or "atmospheric blocking" or "fossil fuel combustion*" or "urban heat island" or "UHI" or "environmental stressor" or "heat wave*" or "cold wave*" or "drought*" or "flood*" or ("wildfire*" or "bushfire*" or "forest fire*") or "heavy precipitation" or "extreme weather event*" or "dust storm*" or "dry period*" or "extreme meteorological event*" or "allergen" or "smog" or "sand storm*" or "heavy snow" or "extreme heat event*" or "aridity" or "air pollution*" or "Ozone" or "particulate matter" or "PM" or "PM2.5" or "ozone*" or "natural disasters" or "particulate matter")) AND (TITLE-ABS-KEY ("Europ*" or "EU" or "European Union" or "continental Europe" or "European region*" or "European countr*"))	4569
**#28**	#27 AND #26<br>((TITLE-ABS-KEY ("well?being" OR "psychological restoration" OR "mental restoration" OR "life W/2 quality" OR "life W/2 satisfaction" OR ("mental*" OR "psychological*" OR "psychos*" OR "psychiatric*") OR "sleep*" OR "insomnia" OR "post-traumatic*" OR "PTSD" OR "trauma" OR "physical W/2 stress*" OR "psychological W/2 stress*" OR "mental adj2 stress*" OR "stress disorder*" OR "stress disease*" OR "depressi*" OR "MDD" OR "mood*" OR ("cognitive*" OR "cognition") OR "disorder*" OR "mania" OR "phobia" OR "anxiet*" OR "behavio* W/2 disorder" OR "behavio* W/2 problem*" OR "behavio* W/2 change*" OR "hyperactiv*" OR "hyper sensitive" OR ("grief" OR "sorrow" OR "sad" OR "sadness" OR "fear" OR "anger" OR "frustration" OR "guilt" OR "helpless*" OR "powerless*" OR "exhaustion" OR "lost" OR "lonel*" OR "overwhelm*" OR "panic*") OR "sense of safety" OR "sense of security" OR "happiness" OR "solastalgia" OR ("adjustment problem*" OR "learning problem*"))) AND (TITLE-ABS-KEY ("climate change*" or "global warming" or "climate variability*" or "anthropogenic warming" or "environmental change*" or "climatic change*" or "burning fossil fuel*" or "atmospheric warm*" or "increased humidity" or "climate hazard*" or "climate crisis" or "rising temperature*" or "precipitation change*" or "sea level rise" or "environmental impact*" or "environmental disaster*" or "climate adaptation*" or "albedo change*" or "rising sea levels" or "earth warming" or "temperature change*" or "environmental amenit*" or "environmental dynamic*" or "increased CO2 emission*" or "carbon dioxide emission*" or "methane emission*" or "CH4 emission*" or "hot climate*" or "NO2 emission*" or "nitrogen dioxide emission*" or "warming planet" or "north Atlantic oscillation*" or "NAO" or "humidity change*" or "increased radiative forcing" or "radiative forcing scenario*" or "dirty fuel*" or "atmospheric blocking" or "fossil fuel combustion*" or "urban heat island" or "UHI" or "environmental stressor" or "heat wave*" or "cold wave*" or "drought*" or "flood*" or ("wildfire*" or "bushfire*" or "forest fire*") or "heavy precipitation" or "extreme weather event*" or "dust storm*" or "dry period*" or "extreme meteorological event*" or "allergen" or "smog" or "sand storm*" or "heavy snow" or "extreme heat event*" or "aridity" or "air pollution*" or "Ozone" or "particulate matter" or "PM" or "PM2.5" or "ozone*" or "natural disasters" or "particulate matter")) AND (TITLE-ABS-KEY ("Europ*" or "EU" or "European Union" or "continental Europe" or "European region*" or "European countr*"))) AND ((TITLE-ABS-KEY ("surrounding*" or "natural space*" or "natural environment" or "park*" or "green*" or "vegetation" or "forest*" or "woodland*" or "meadow*" or "grassland" or "shrubland" or "crop*" or "agricultural area*" or "cultivated land*" or "land use mix" or "blue*" or "wetland*" or ("water body" or "water bodies") or "pond*" or "lake*" or "beach*" or "coastal water*" or "pool" or "inland-blue space" or "river*" or "canal*" or "coastal-blue space*" or "esplanade*" or ("built environment*" or "built?up area*") or "grey space*" or "street*" or "residential area*" or ("housing W/2 area*" or "neighbo?rhood*" or "living W/2 area*") or "livestock*" or "waste dump*")) OR (TITLE-ABS-KEY ("education*" or "learning")) OR (TITLE-ABS-KEY ("living situation" or "housing situation" or "ownership right*" or "ownership status" or "house*" or "household*" or "living W/2 space" or ("air condition*" or "air cooling") or "heating" or "ventilation")) OR (TITLE-ABS-KEY ("exercise*" or "sport*" or "playing" or " hobb*" or "physical* activ*" or "leisure" or "leisure time activity" or "recreation*")) OR (TITLE-ABS-KEY ("employ*" or "unemploy*" or "under?employment" or "work contract*" or "retire*" or "profession*" or "labo?r*" or "occupation*" or "work alienation" or "outdoor worker*" or "commuter*" or "office worker*")) OR (TITLE-ABS-KEY ("Cycling" or "running" or ("active transport*" or "public transport" or "passive transport") or "travel*" or "active movement*" or "walking" or "travel W/2 time" or "choice of transportation" or ("independent mobility" or "independent travel"))) OR (TITLE-ABS-KEY ("goods" or "subsistence*" or "consumption*")) OR (TITLE-ABS-KEY ("Income" or "salary W/2 level" or "financial*" or "economic strains" or "unemployment adj2 rate*" or "livelihood*")) OR (TITLE-ABS-KEY ("civil status" or "marital status" or "societ*" or "communit*" or "support*" or "social*" or "famil*" or "lifestyle" or "isolation" or "sense of loneliness" or ("parental anxiety*" or "parental stress" or "parental depression*"))) OR (TITLE-ABS-KEY (genetic* or "inherited*" or motivation* or illness* or awareness or "allerg*" or "breastfeed*" or "formula feeding" or "belief*" or "age" or ("sex" or "gender") or ("prematurity" or "birth weight") or "environmental value*" or "environmental concern*" or "environmental sensitivity" or "connectedness with nature")) OR (TITLE-ABS-KEY ("socio-economic status" or "SES")))	3840
**#29**	#28 AND PUBYEAR > 2010 AND PUBYEAR < 2024<br>((TITLE-ABS-KEY ("well?being" OR "psychological restoration" OR "mental restoration" OR "life W/2 quality" OR "life W/2 satisfaction"	2168

	OR ("mental*" OR "psychological*" OR "psychos*" OR "psychiatric*") OR "sleep*" OR "insomnia" OR "post-traumatic*" OR "PTSD" OR "trauma" OR "physical W/2 stress*" OR "psychological W/2 stress*" OR "mental adj2 stress*" OR "stress disorder*" OR "stress disease*" OR "depressi*" OR "MDD" OR "mood*" OR ("cognitive*" OR "cognition") OR "disorder*" OR "mania" OR "phobia" OR "anxiet*" OR "behavio* W/2 disorder" OR "behavio* W/2 problem*" OR "behavio* W/2 change*" OR "hyperactiv*" OR "hyper sensitive" OR ("grief" OR "sorrow" OR "sad" OR "sadness" OR "fear" OR "anger" OR "frustration" OR "guilt" OR "helpless*" OR "powerless*" OR "exhaustion" OR "lost" OR "lonel*" OR "overwhelm*" OR "panic*") OR "sense of safety" OR "sense of security" OR "happiness" OR "solastalgia" OR ("adjustment problem*" OR "learning problem*"))) AND (TITLE-ABS-KEY ("climate change*" or "global warming" or "climate variability*" or "anthropogenic warming" or "environmental change*" or "climatic change*" or "burning fossil fuel*" or "atmospheric warm*" or "increased humidity" or "climate hazard*" or "climate crisis" or "rising temperature*" or "precipitation change*" or "sea level rise" or "environmental impact*" or "environmental disaster*" or "climate adaptation*" or "albedo change*" or "rising sea levels" or "earth warming" or "temperature change*" or "environmental amenit*" or "environmental dynamic*" or "increased CO2 emission*" or "carbon dioxide emission*" or "methane emission*" or "CH4 emission*" or "hot climate*" or "NO2 emission*" or "nitrogen dioxide emission*" or "warming planet" or "north Atlantic oscillation*" or "NAO" or "humidity change*" or "increased radiative forcing" or "radiative forcing scenario*" or "dirty fuel*" or "atmospheric blocking" or "fossil fuel combustion*" or "urban heat island" or "UHI" or "environmental stressor" or "heat wave*" or "cold wave*" or "drought*" or "flood*" or ("wildfire*" or "bushfire*" or "forest fire*") or "heavy precipitation" or "extreme weather event*" or "dust storm*" or "dry period*" or "extreme meteorological event*" or "allergen" or "smog" or "sand storm*" or "heavy snow" or "extreme heat event*" or "aridity" or "air pollution*" or "Ozone" or "particulate matter" or "PM" or "PM2.5" or "ozone*" or "natural disasters" or "particulate matter")) AND (TITLE-ABS-KEY ("Europ*" or "EU" or "European Union" or "continental Europe" or "European region*" or "European countr*"))) AND ((TITLE-ABS-KEY ("surrounding*" or "natural space*" or "natural environment" or "park*" or "green*" or "vegetation" or "forest*" or "woodland*" or "meadow*" or "grassland" or "shrubland" or "crop*" or "agricultural area*" or "cultivated land*" or "land use mix" or "blue*" or "wetland*" or ("water body" or "water bodies") or "pond*" or "lake*" or "beach*" or "coastal water*" or "pool" or "inland-blue space" or "river*" or "canal*" or "coastal-blue space*" or "esplanade*" or ("built environment*" or "built?up area*") or "grey space*" or "street*" or "residential area*" or ("housing W/2 area*" or "neighbo?rhood*" or "living W/2 area*") or "livestock*" or "waste dump*")) OR (TITLE-ABS-KEY ("education*" or "learning")) OR (TITLE-ABS-KEY ("living situation" or "housing situation" or "ownership right*" or "ownership status" or "house*" or "household*" or "living W/2 space" or ("air condition*" or "air cooling") or "heating" or "ventilation")) OR (TITLE-ABS-KEY ("exercise*" or "sport*" or "playing" or " hobb*" or "physical* activ*" or "leisure" or "leisure time activity" or "recreation*")) OR (TITLE-ABS-KEY ("employ*" or "unemploy*" or "under?employment" or "work contract*" or "retire*" or "profession*" or "labo?r*" or "occupation*" or "work alienation" or "outdoor worker*" or "commuter*" or "office worker*")) OR (TITLE-ABS-KEY ("Cycling" or "running" or ("active transport*" or "public transport" or "passive transport") or "travel*" or "active movement*" or "walking" or "travel W/2 time" or "choice of transportation" or ("independent mobility" or "independent travel"))) OR (TITLE-ABS-KEY ("goods" or "subsistence*" or "consumption*")) OR (TITLE-ABS-KEY ("Income" or "salary W/2 level" or "financial*" or "economic strains" or "unemployment adj2 rate*" or "livelihood*")) OR (TITLE-ABS-KEY ("civil status" or "marital status" or "societ*" or "communit*" or "support*" or "social*" or "famil*" or "lifestyle" or "isolation" or "sense of loneliness" or ("parental anxiety*" or "parental stress" or "parental depression*"))) OR (TITLE-ABS-KEY (genetic* or "inherited*" or motivation* or illness* or awareness or "allerg*" or "breastfeed*" or "formula feeding" or "belief*" or "age" or ("sex" or "gender") or ("prematurity" or "birth weight") or "environmental value*" or "environmental concern*" or "environmental sensitivity" or "connectedness with nature")) OR (TITLE-ABS-KEY ("socio-economic status" or "SES"))) AND PUBYEAR > 2010 AND PUBYEAR < 2024 (2,168)	
**#33**	#29 AND (LIMIT-TO (DOCTYPE , "ar")) AND (LIMIT-TO (LANGUAGE , "English"))<br>(((TITLE-ABS-KEY ("well?being" OR "psychological restoration" OR "mental restoration" OR "life W/2 quality" OR "life W/2 satisfaction" OR ("mental*" OR "psychological*" OR "psychos*" OR "psychiatric*") OR "sleep*" OR "insomnia" OR "post-traumatic*" OR "PTSD" OR "trauma" OR "physical W/2 stress*" OR "psychological W/2 stress*" OR "mental adj2 stress*" OR "stress disorder*" OR "stress disease*" OR "depressi*" OR "MDD" OR "mood*" OR ("cognitive*" OR "cognition") OR "disorder*" OR "mania" OR "phobia" OR "anxiet*" OR "behavio* W/2 disorder" OR "behavio* W/2 problem*" OR "behavio* W/2 change*" OR "hyperactiv*" OR "hyper sensitive" OR ("grief" OR "sorrow" OR "sad" OR "sadness" OR "fear" OR "anger" OR "frustration" OR "guilt" OR "helpless*" OR "powerless*" OR "exhaustion" OR "lost" OR "lonel*" OR "overwhelm*" OR "panic*") OR "sense of safety" OR "sense of security" OR "happiness" OR "solastalgia" OR ("adjustment problem*" OR "learning problem*"))) AND (TITLE-ABS-KEY ("climate change*" or "global warming" or "climate variability*" or "anthropogenic warming" or "environmental change*" or "climatic change*" or "burning fossil fuel*" or "atmospheric warm*" or "increased humidity" or "climate hazard*" or "climate crisis" or "rising temperature*" or "precipitation change*" or "sea level rise" or "environmental impact*" or "environmental disaster*" or "climate adaptation*" or "albedo change*" or "rising sea levels" or "earth warming" or "temperature change*" or "environmental amenit*" or "environmental dynamic*" or "increased CO2 emission*" or "carbon dioxide emission*" or "methane emission*" or "CH4 emission*" or "hot climate*" or "NO2 emission*" or "nitrogen dioxide emission*" or "warming planet" or "north Atlantic oscillation*" or "NAO" or "humidity change*" or "increased radiative forcing" or "radiative forcing scenario*" or "dirty fuel*" or "atmospheric blocking" or "fossil fuel combustion*" or "urban heat island" or "UHI" or "environmental stressor" or "heat wave*" or "cold wave*" or "drought*" or "flood*" or ("wildfire*" or "bushfire*" or "forest fire*") or "heavy precipitation" or "extreme weather event*" or "dust storm*" or "dry period*" or "extreme meteorological event*" or "allergen" or "smog" or "sand storm*" or "heavy snow" or "extreme heat event*" or "aridity" or "air pollution*" or "Ozone" or "particulate matter" or "PM" or "PM2.5" or "ozone*" or "natural disasters" or "particulate matter")) AND (TITLE-ABS-KEY ("Europ*" or "EU" or "European Union" or "continental Europe" or "European region*" or "European countr*"))) AND ((TITLE-ABS-KEY ("surrounding*" or "natural space*" or "natural environment" or "park*" or "green*" or "vegetation" or "forest*" or "woodland*" or "meadow*" or "grassland" or "shrubland" or "crop*" or "agricultural area*" or "cultivated land*" or "land use mix" or "blue*" or "wetland*" or ("water body" or "water bodies") or "pond*" or "lake*" or "beach*" or "coastal water*" or "pool" or "inland-blue space" or "river*" or "canal*" or "coastal-blue space*" or "esplanade*" or ("built environment*" or "built?up area*") or "grey space*" or "street*" or "residential area*" or ("housing W/2 area*" or "neighbo?rhood*" or "living W/2 area*") or "livestock*" or "waste dump*")) OR (TITLE-ABS-KEY ("education*" or "learning")) OR (TITLE-ABS-KEY ("living situation" or "housing situation" or "ownership right*" or "ownership status" or "house*" or "household*" or "living W/2 space" or ("air condition*" or "air cooling") or "heating" or "ventilation")) OR (TITLE-ABS-KEY ("exercise*" or "sport*" or "playing" or " hobb*" or "physical* activ*" or "leisure" or "leisure time activity" or "recreation*")) OR (TITLE-ABS-KEY ("employ*" or "unemploy*" or "under?employment" or "work contract*" or "retire*" or "profession*" or "labo?r*" or "occupation*" or "work alienation" or "outdoor worker*" or "commuter*" or "office worker*")) OR (TITLE-ABS-KEY ("Cycling" or "running" or ("active transport*" or "public transport" or "passive transport") or "travel*" or "active movement*" or "walking" or "travel W/2 time" or "choice of transportation" or ("independent mobility" or "independent travel"))) OR (TITLE-ABS-KEY ("goods" or "subsistence*" or "consumption*")) OR (TITLE-ABS-KEY ("Income" or "salary W/2 level" or "financial*" or "economic strains" or "unemployment adj2 rate*" or "livelihood*")) OR (TITLE-ABS-KEY ("civil status" or "marital status" or "societ*" or "communit*" or "support*" or "social*" or "famil*" or "lifestyle" or "isolation" or "sense of loneliness" or ("parental anxiety*" or "parental stress" or "parental depression*"))) OR (TITLE-ABS-KEY (genetic* or "inherited*" or motivation* or illness* or awareness or "allerg*" or "breastfeed*" or "formula feeding" or "belief*" or "age" or ("sex" or "gender") or ("prematurity" or "birth weight") or "environmental value*" or "environmental concern*" or "environmental sensitivity" or "connectedness with nature")) OR (TITLE-ABS-KEY ("socio-economic status" or "SES"))) AND PUBYEAR > 2010 AND PUBYEAR < 2024) AND ((TITLE-ABS-KEY ("surrounding*" or "natural space*" or "natural environment" or "park*" or "green*" or "vegetation" or "forest*" or "woodland*" or "meadow*" or "grassland" or "shrubland" or "crop*" or "agricultural area*" or "cultivated land*" or "land use mix" or "blue*" or "wetland*" or ("water body" or "water bodies") or "pond*" or "lake*" or "beach*" or "coastal water*" or "pool" or "inland-blue space" or "river*" or "canal*" or "coastal-blue space*" or "esplanade*" or ("built environment*" or "built?up area*") or "grey space*" or "street*" or "residential area*" or ("housing W/2 area*" or "neighbo?rhood*" or "living W/2 area*") or "livestock*" or	1413

	"waste dump*")) OR (TITLE-ABS-KEY ("education*" or "learning")) OR (TITLE-ABS-KEY ("living situation" or "housing situation" or "ownership right*" or "ownership status" or "house*" or "household*" or "living W/2 space" or ("air condition*" or "air cooling") or "heating" or "ventilation")) OR (TITLE-ABS-KEY ("exercise*" or "sport*" or "playing" or " hobb*" or "physical* activ*" or "leisure" or "leisure time activity" or "recreation*")) OR (TITLE-ABS-KEY ("employ*" or "unemploy*" or "under?employment" or "work contract*" or "retire*" or "profession*" or "labo?r*" or "occupation*" or "work alienation" or "outdoor worker*" or "commuter*" or "office worker*")) OR (TITLE-ABS-KEY ("Cycling" or "running" or ("active transport*" or "public transport" or "passive transport") or "travel*" or "active movement*" or "walking" or "travel W/2 time" or "choice of transportation" or ("independent mobility" or "independent travel"))) OR (TITLE-ABS-KEY ("goods" or "subsistence*" or "consumption*")) OR (TITLE-ABS-KEY ("Income" or "salary W/2 level" or "financial*" or "economic strains" or "unemployment adj2 rate*" or "livelihood*")) OR (TITLE-ABS-KEY ("civil status" or "marital status" or "societ*" or "communit*" or "support*" or "social*" or "famil*" or "lifestyle" or "isolation" or "sense of loneliness" or ("parental anxiety*" or "parental stress" or "parental depression*"))) OR (TITLE-ABS-KEY (genetic* or "inherited*" or motivation* or illness* or awareness or "allerg*" or "breastfeed*" or "formula feeding" or "belief*" or "age" or ("sex" or "gender") or ("prematurity" or "birth weight") or "environmental value*" or "environmental concern*" or "environmental sensitivity" or "connectedness with nature")) OR (TITLE-ABS-KEY ("socio-economic status" or "SES"))) AND PUBYEAR > 2010 AND PUBYEAR < 2024 AND (LIMIT-TO (DOCTYPE , "ar")) AND (LIMIT-TO (LANGUAGE , "English"))	
**#34**	TITLE-ABS-KEY (("systematic review" or "review"))	6806237
**#35**	TITLE-ABS-KEY (("RCT" or "randomi?ed control trail"))	52376
**#36**	#33 AND NOT #34<br>((((TITLE-ABS-KEY ("well?being" OR "psychological restoration" OR "mental restoration" OR "life W/2 quality" OR "life W/2 satisfaction" OR ("mental*" OR "psychological*" OR "psychos*" OR "psychiatric*") OR "sleep*" OR "insomnia" OR "post-traumatic*" OR "PTSD" OR "trauma" OR "physical W/2 stress*" OR "psychological W/2 stress*" OR "mental adj2 stress*" OR "stress disorder*" OR "stress disease*" OR "depressi*" OR "MDD" OR "mood*" OR ("cognitive*" OR "cognition") OR "disorder*" OR "mania" OR "phobia" OR "anxiet*" OR "behavio* W/2 disorder" OR "behavio* W/2 problem*" OR "behavio* W/2 change*" OR "hyperactiv*" OR "hyper sensitive" OR ("grief" OR "sorrow" OR "sad" OR "sadness" OR "fear" OR "anger" OR "frustration" OR "guilt" OR "helpless*" OR "powerless*" OR "exhaustion" OR "lost" OR "lonel*" OR "overwhelm*" OR "panic*") OR "sense of safety" OR "sense of security" OR "happiness" OR "solastalgia" OR ("adjustment problem*" OR "learning problem*"))) AND (TITLE-ABS-KEY ("climate change*" or "global warming" or "climate variability*" or "anthropogenic warming" or "environmental change*" or "climatic change*" or "burning fossil fuel*" or "atmospheric warm*" or "increased humidity" or "climate hazard*" or "climate crisis" or "rising temperature*" or "precipitation change*" or "sea level rise" or "environmental impact*" or "environmental disaster*" or "climate adaptation*" or "albedo change*" or "rising sea levels" or "earth warming" or "temperature change*" or "environmental amenit*" or "environmental dynamic*" or "increased CO2 emission*" or "carbon dioxide emission*" or "methane emission*" or "CH4 emission*" or "hot climate*" or "NO2 emission*" or "nitrogen dioxide emission*" or "warming planet" or "north Atlantic oscillation*" or "NAO" or "humidity change*" or "increased radiative forcing" or "radiative forcing scenario*" or "dirty fuel*" or "atmospheric blocking" or "fossil fuel combustion*" or "urban heat island" or "UHI" or "environmental stressor" or "heat wave*" or "cold wave*" or "drought*" or "flood*" or ("wildfire*" or "bushfire*" or "forest fire*") or "heavy precipitation" or "extreme weather event*" or "dust storm*" or "dry period*" or "extreme meteorological event*" or "allergen" or "smog" or "sand storm*" or "heavy snow" or "extreme heat event*" or "aridity" or "air pollution*" or "Ozone" or "particulate matter" or "PM" or "PM2.5" or "ozone*" or "natural disasters" or "particulate matter")) AND (TITLE-ABS-KEY ("Europ*" or "EU" or "European Union" or "continental Europe" or "European region*" or "European countr*"))) AND ((TITLE-ABS-KEY ("surrounding*" or "natural space*" or "natural environment" or "park*" or "green*" or "vegetation" or "forest*" or "woodland*" or "meadow*" or "grassland" or "shrubland" or "crop*" or "agricultural area*" or "cultivated land*" or "land use mix" or "blue*" or "wetland*" or ("water body" or "water bodies") or "pond*" or "lake*" or "beach*" or "coastal water*" or "pool" or "inland-blue space" or "river*" or "canal*" or "coastal-blue space*" or "esplanade*" or ("built environment*" or "built?up area*") or "grey space*" or "street*" or "residential area*" or ("housing W/2 area*" or "neighbo?rhood*" or "living W/2 area*") or "livestock*" or "waste dump*")) OR (TITLE-ABS-KEY ("education*" or "learning")) OR (TITLE-ABS-KEY ("living situation" or "housing situation" or "ownership right*" or "ownership status" or "house*" or "household*" or "living W/2 space" or ("air condition*" or "air cooling") or "heating" or "ventilation")) OR (TITLE-ABS-KEY ("exercise*" or "sport*" or "playing" or " hobb*" or "physical* activ*" or "leisure" or "leisure time activity" or "recreation*")) OR (TITLE-ABS-KEY ("employ*" or "unemploy*" or "under?employment" or "work contract*" or "retire*" or "profession*" or "labo?r*" or "occupation*" or "work alienation" or "outdoor worker*" or "commuter*" or "office worker*")) OR (TITLE-ABS-KEY ("Cycling" or "running" or ("active transport*" or "public transport" or "passive transport") or "travel*" or "active movement*" or "walking" or "travel W/2 time" or "choice of transportation" or ("independent mobility" or "independent travel"))) OR (TITLE-ABS-KEY ("goods" or "subsistence*" or "consumption*")) OR (TITLE-ABS-KEY ("Income" or "salary W/2 level" or "financial*" or "economic strains" or "unemployment adj2 rate*" or "livelihood*")) OR (TITLE-ABS-KEY ("civil status" or "marital status" or "societ*" or "communit*" or "support*" or "social*" or "famil*" or "lifestyle" or "isolation" or "sense of loneliness" or ("parental anxiety*" or "parental stress" or "parental depression*"))) OR (TITLE-ABS-KEY (genetic* or "inherited*" or motivation* or illness* or awareness or "allerg*" or "breastfeed*" or "formula feeding" or "belief*" or "age" or ("sex" or "gender") or ("prematurity" or "birth weight") or "environmental value*" or "environmental concern*" or "environmental sensitivity" or "connectedness with nature")) OR (TITLE-ABS-KEY ("socio-economic status" or "SES"))) AND PUBYEAR > 2010 AND PUBYEAR < 2024) AND ((TITLE-ABS-KEY ("surrounding*" or "natural space*" or "natural environment" or "park*" or "green*" or "vegetation" or "forest*" or "woodland*" or "meadow*" or "grassland" or "shrubland" or "crop*" or "agricultural area*" or "cultivated land*" or "land use mix" or "blue*" or "wetland*" or ("water body" or "water bodies") or "pond*" or "lake*" or "beach*" or "coastal water*" or "pool" or "inland-blue space" or "river*" or "canal*" or "coastal-blue space*" or "esplanade*" or ("built environment*" or "built?up area*") or "grey space*" or "street*" or "residential area*" or ("housing W/2 area*" or "neighbo?rhood*" or "living W/2 area*") or "livestock*" or "waste dump*")) OR (TITLE-ABS-KEY ("education*" or "learning")) OR (TITLE-ABS-KEY ("living situation" or "housing situation" or "ownership right*" or "ownership status" or "house*" or "household*" or "living W/2 space" or ("air condition*" or "air cooling") or "heating" or "ventilation")) OR (TITLE-ABS-KEY ("exercise*" or "sport*" or "playing" or " hobb*" or "physical* activ*" or "leisure" or "leisure time activity" or "recreation*")) OR (TITLE-ABS-KEY ("employ*" or "unemploy*" or "under?employment" or "work contract*" or "retire*" or "profession*" or "labo?r*" or "occupation*" or "work alienation" or "outdoor worker*" or "commuter*" or "office worker*")) OR (TITLE-ABS-KEY ("Cycling" or "running" or ("active transport*" or "public transport" or "passive transport") or "travel*" or "active movement*" or "walking" or "travel W/2 time" or "choice of transportation" or ("independent mobility" or "independent travel"))) OR (TITLE-ABS-KEY ("goods" or "subsistence*" or "consumption*")) OR (TITLE-ABS-KEY ("Income" or "salary W/2 level" or "financial*" or "economic strains" or "unemployment adj2 rate*" or "livelihood*")) OR (TITLE-ABS-KEY ("civil status" or "marital status" or "societ*" or "communit*" or "support*" or "social*" or "famil*" or "lifestyle" or "isolation" or "sense of loneliness" or ("parental anxiety*" or "parental stress" or "parental depression*"))) OR (TITLE-ABS-KEY (genetic* or "inherited*" or motivation* or illness* or awareness or "allerg*" or "breastfeed*" or "formula feeding" or "belief*" or "age" or ("sex" or "gender") or ("prematurity" or "birth weight") or "environmental value*" or "environmental concern*" or "environmental sensitivity" or "connectedness with nature")) OR (TITLE-ABS-KEY ("socio-economic status" or "SES"))) AND PUBYEAR > 2010 AND PUBYEAR < 2024) AND NOT (TITLE-ABS-KEY (("systematic review" or "review"))) AND (LIMIT-TO (DOCTYPE , "ar")) AND (LIMIT-TO (LANGUAGE , "English"))	1319
**#37**	#36 AND NOT #35<br>(((((TITLE-ABS-KEY ("well?being" OR "psychological restoration" OR "mental restoration" OR "life W/2 quality" OR "life W/2 satisfaction" OR ("mental*" OR "psychological*" OR "psychos*" OR "psychiatric*") OR "sleep*" OR "insomnia" OR "post-traumatic*" OR "PTSD" OR "trauma" OR "physical W/2 stress*" OR "psychological W/2 stress*" OR "mental adj2 stress*" OR "stress disorder*" OR "stress disease*" OR "depressi*" OR "MDD" OR "mood*" OR ("cognitive*" OR "cognition") OR "disorder*" OR "mania" OR "phobia" OR	1317

	"anxiet*" OR "behavio* W/2 disorder" OR "behavio* W/2 problem*" OR "behavio* W/2 change*" OR "hyperactiv*" OR "hyper sensitive" OR ("grief" OR "sorrow" OR "sad" OR "sadness" OR "fear" OR "anger" OR "frustration" OR "guilt" OR "helpless*" OR "powerless*" OR "exhaustion" OR "lost" OR "lonel*" OR "overwhelm*" OR "panic*") OR "sense of safety" OR "sense of security" OR "happiness" OR "solastalgia" OR ("adjustment problem*" OR "learning problem*"))) AND (TITLE-ABS-KEY ("climate change*" or "global warming" or "climate variability*" or "anthropogenic warming" or "environmental change*" or "climatic change*" or "burning fossil fuel*" or "atmospheric warm*" or "increased humidity" or "climate hazard*" or "climate crisis" or "rising temperature*" or "precipitation change*" or "sea level rise" or "environmental impact*" or "environmental disaster*" or "climate adaptation*" or "albedo change*" or "rising sea levels" or "earth warming" or "temperature change*" or "environmental amenit*" or "environmental dynamic*" or "increased CO2 emission*" or "carbon dioxide emission*" or "methane emission*" or "CH4 emission*" or "hot climate*" or "NO2 emission*" or "nitrogen dioxide emission*" or "warming planet" or "north Atlantic oscillation*" or "NAO" or "humidity change*" or "increased radiative forcing" or "radiative forcing scenario*" or "dirty fuel*" or "atmospheric blocking" or "fossil fuel combustion*" or "urban heat island" or "UHI" or "environmental stressor" or "heat wave*" or "cold wave*" or "drought*" or "flood*" or ("wildfire*" or "bushfire*" or "forest fire*") or "heavy precipitation" or "extreme weather event*" or "dust storm*" or "dry period*" or "extreme meteorological event*" or "allergen" or "smog" or "sand storm*" or "heavy snow" or "extreme heat event*" or "aridity" or "air pollution*" or "Ozone" or "particulate matter" or "PM" or "PM2.5" or "ozone*" or "natural disasters" or "particulate matter")) AND (TITLE-ABS-KEY ("Europ*" or "EU" or "European Union" or "continental Europe" or "European region*" or "European countr*"))) AND ((TITLE-ABS-KEY ("surrounding*" or "natural space*" or "natural environment" or "park*" or "green*" or "vegetation" or "forest*" or "woodland*" or "meadow*" or "grassland" or "shrubland" or "crop*" or "agricultural area*" or "cultivated land*" or "land use mix" or "blue*" or "wetland*" or ("water body" or "water bodies") or "pond*" or "lake*" or "beach*" or "coastal water*" or "pool" or "inland-blue space" or "river*" or "canal*" or "coastal-blue space*" or "esplanade*" or ("built environment*" or "built?up area*") or "grey space*" or "street*" or "residential area*" or ("housing W/2 area*" or "neighbo?rhood*" or "living W/2 area*") or "livestock*" or "waste dump*")) OR (TITLE-ABS-KEY ("education*" or "learning")) OR (TITLE-ABS-KEY ("living situation" or "housing situation" or "ownership right*" or "ownership status" or "house*" or "household*" or "living W/2 space" or ("air condition*" or "air cooling") or "heating" or "ventilation")) OR (TITLE-ABS-KEY ("exercise*" or "sport*" or "playing" or " hobb*" or "physical* activ*" or "leisure" or "leisure time activity" or "recreation*")) OR (TITLE-ABS-KEY ("employ*" or "unemploy*" or "under?employment" or "work contract*" or "retire*" or "profession*" or "labo?r*" or "occupation*" or "work alienation" or "outdoor worker*" or "commuter*" or "office worker*")) OR (TITLE-ABS-KEY ("Cycling" or "running" or ("active transport*" or "public transport" or "passive transport") or "travel*" or "active movement*" or "walking" or "travel W/2 time" or "choice of transportation" or ("independent mobility" or "independent travel"))) OR (TITLE-ABS-KEY ("goods" or "subsistence*" or "consumption*")) OR (TITLE-ABS-KEY ("Income" or "salary W/2 level" or "financial*" or "economic strains" or "unemployment adj2 rate*" or "livelihood*")) OR (TITLE-ABS-KEY ("civil status" or "marital status" or "societ*" or "communit*" or "support*" or "social*" or "famil*" or "lifestyle" or "isolation" or "sense of loneliness" or ("parental anxiety*" or "parental stress" or "parental depression*"))) OR (TITLE-ABS-KEY (genetic* or "inherited*" or motivation* or illness* or awareness or "allerg*" or "breastfeed*" or "formula feeding" or "belief*" or "age" or ("sex" or "gender") or ("prematurity" or "birth weight") or "environmental value*" or "environmental concern*" or "environmental sensitivity" or "connectedness with nature")) OR (TITLE-ABS-KEY ("socio-economic status" or "SES"))) AND PUBYEAR > 2010 AND PUBYEAR < 2024) AND ((TITLE-ABS-KEY ("surrounding*" or "natural space*" or "natural environment" or "park*" or "green*" or "vegetation" or "forest*" or "woodland*" or "meadow*" or "grassland" or "shrubland" or "crop*" or "agricultural area*" or "cultivated land*" or "land use mix" or "blue*" or "wetland*" or ("water body" or "water bodies") or "pond*" or "lake*" or "beach*" or "coastal water*" or "pool" or "inland-blue space" or "river*" or "canal*" or "coastal-blue space*" or "esplanade*" or ("built environment*" or "built?up area*") or "grey space*" or "street*" or "residential area*" or ("housing W/2 area*" or "neighbo?rhood*" or "living W/2 area*") or "livestock*" or "waste dump*")) OR (TITLE-ABS-KEY ("education*" or "learning")) OR (TITLE-ABS-KEY ("living situation" or "housing situation" or "ownership right*" or "ownership status" or "house*" or "household*" or "living W/2 space" or ("air condition*" or "air cooling") or "heating" or "ventilation")) OR (TITLE-ABS-KEY ("exercise*" or "sport*" or "playing" or " hobb*" or "physical* activ*" or "leisure" or "leisure time activity" or "recreation*")) OR (TITLE-ABS-KEY ("employ*" or "unemploy*" or "under?employment" or "work contract*" or "retire*" or "profession*" or "labo?r*" or "occupation*" or "work alienation" or "outdoor worker*" or "commuter*" or "office worker*")) OR (TITLE-ABS-KEY ("Cycling" or "running" or ("active transport*" or "public transport" or "passive transport") or "travel*" or "active movement*" or "walking" or "travel W/2 time" or "choice of transportation" or ("independent mobility" or "independent travel"))) OR (TITLE-ABS-KEY ("goods" or "subsistence*" or "consumption*")) OR (TITLE-ABS-KEY ("Income" or "salary W/2 level" or "financial*" or "economic strains" or "unemployment adj2 rate*" or "livelihood*")) OR (TITLE-ABS-KEY ("civil status" or "marital status" or "societ*" or "communit*" or "support*" or "social*" or "famil*" or "lifestyle" or "isolation" or "sense of loneliness" or ("parental anxiety*" or "parental stress" or "parental depression*"))) OR (TITLE-ABS-KEY (genetic* or "inherited*" or motivation* or illness* or awareness or "allerg*" or "breastfeed*" or "formula feeding" or "belief*" or "age" or ("sex" or "gender") or ("prematurity" or "birth weight") or "environmental value*" or "environmental concern*" or "environmental sensitivity" or "connectedness with nature")) OR (TITLE-ABS-KEY ("socio-economic status" or "SES"))) AND PUBYEAR > 2010 AND PUBYEAR < 2024) AND NOT (TITLE-ABS-KEY (("systematic review" or "review")))) AND NOT (TITLE-ABS-KEY (("RCT" or "randomi?ed control trail"))) AND (LIMIT-TO (DOCTYPE , "ar")) AND (LIMIT-TO (LANGUAGE , "English"))	

S3. Risk of bias assessment scales
In the risk of bias assessment, the Newcastle – Ottawa – Scale was used for case control studies, cohort studies and the adapted version for cross-sectional studies. For qualitative studies the CASP Scale was used. Case-crossover studies were treated as cross-sectional studies.

NEWCASTLE - OTTAWA QUALITY ASSESSMENT SCALE(Wells et al., 2021)
CASE CONTROL STUDIES

Selection (Maximum 4 stars)

1) Is the case definition adequate?
   a) yes, with independent validation ✵
   b) yes, e.g. record linkage or based on self-reports
   c) no description

2) Representativeness of the cases
   a) consecutive or obviously representative series of cases ✵
   b) potential for selection biases or not stated

3) Selection of Controls
   a) community controls ✵
   b) hospital controls
   c) no description

4) Definition of Controls
   a) no history of disease (endpoint) ✵
   b) no description of source

Comparability (Maximum 2 stars)

1) Comparability of cases and controls on the basis of the design or analysis
   a) study controls for _______________ (Select the most important factor.) ✵
   b) study controls for any additional factor ✵ (This criteria could be modified to indicate specific control for a second important factor.)

Exposure (Maximum 3 stars)

1) Ascertainment of exposure
   a) secure record (eg surgical records) ✵
   b) structured interview where blind to case/control status ✵
   c) interview not blinded to case/control status
   d) written self-report or medical record only
   e) no description

2) Same method of ascertainment for cases and controls
   a) yes ✵
   b) no

3) Non-Response rate
   a) same rate for both groups ✵
   b) non respondents described
   c) rate different and no designation

NEWCASTLE - OTTAWA QUALITY ASSESSMENT SCALE
COHORT STUDIES

Selection (Maximum 4 stars)

1) Representativeness of the exposed cohort
   a) truly representative of the average _______________ (describe) in the community ❋
   b) somewhat representative of the average ______________ in the community ❋
   c) selected group of users eg nurses, volunteers
   d) no description of the derivation of the cohort

2) Selection of the non-exposed cohort
   a) drawn from the same community as the exposed cohort ❋
   b) drawn from a different source
   c) no description of the derivation of the non-exposed cohort

3) Ascertainment of exposure
   a) secure record (eg surgical records) □
   b) structured interview ❋
   c) written self-report
   d) no description

4) Demonstration that outcome of interest was not present at start of study
   a) yes ❋
   b) no

Comparability (Maximum 2 stars)

1) Comparability of cohorts on the basis of the design or analysis
   a) study controls for _____________ (select the most important factor) ❋
   b) study controls for any additional factor ❋ (This criteria could be modified to indicate specific control for a second important factor.)

Outcome (Maximum 3 stars)

1) Assessment of outcome
   a) independent blind assessment ❋
   b) record linkage ❋
   c) self-report
   d) no description

2) Was follow-up long enough for outcomes to occur
   a) yes (select an adequate follow up period for outcome of interest) ❋
   b) no

3) Adequacy of follow up of cohorts
   a) complete follow up - all subjects accounted for ❋
   b) subjects lost to follow up unlikely to introduce bias - small number lost - > ____ % (select an adequate %) follow up, or description provided of those lost ❋
   c) follow up rate < ____% (select an adequate %) and no description of those lost
   d) no statement

Adapted Newcastle – Ottawa Scale for cross-sectional studies adopted from Yoo H, et al. 2023(Yoo et al., 2023)

NEWCASTLE - OTTAWA QUALITY ASSESSMENT SCALE
CROSS-SECTIONAL STUDIES

Selection: (Maximum 5 stars)

1) Representativeness of the sample:
   a) Truly representative of the average in the target population. ✵ (all subjects or random sampling)
   b) Somewhat representative of the average in the target population. ✵ (non-random sampling)
   c) Selected group of users.
   d) No description of the sampling strategy.

2) Sample size:
   a) Justified and satisfactory. ✵
   b) Not justified.

3) Non-respondents:
   a) Comparability between respondents and non-respondents characteristics is established, and the response rate is satisfactory. ✵
   b) The response rate is unsatisfactory, or the comparability between respondents and non-respondents is unsatisfactory.
   c) No description of the response rate or the characteristics of the responders and the non-responders.

4) Ascertainment of the exposure (risk factor):
   a) Validated measurement tool. ✵✵
   b) Non-validated measurement tool, but the tool is available or described. ✵
   c) No description of the measurement tool.

Comparability: (Maximum 2 stars)

1) The subjects in different outcome groups are comparable, based on the study design or analysis. Confounding factors are controlled.
   a) The study controls for the most important factors (age, BMI and obstetric history). ✵
   b) The study control for any additional factor (e.g. race/ethnicity, socioeconomic status, smoking status, drinking, hobbies, history of illness, sexual relationships) ✵

Outcome: (Maximum 3 stars)

1) Assessment of the outcome:
   a) Independent blind assessment. ✵✵
   b) Record linkage. ✵✵
   c) Self report. ✵
   d) No description.

2) Statistical test:
   a) The statistical test used to analyze the data is clearly described and appropriate, and the measurement of the association is presented, including confidence intervals and the probability level (p value). *
   b) The statistical test is not appropriate, not described or incomplete.

CRITICAL APPRAISAL SKILLS PROGRAMME[3]
CASP CHECKLIST FOR QUALITATIVE RESEARCH

Section A: Are the results valid?

1) Was there a clear statement of the aims of the research?
   a) yes ❋
   b) can't tell
   c) no

2) Is a qualitative methodology appropriate?
   a) yes ❋
   b) can't tell
   c) no

3) Was the research design appropriate to address the aims of the research?
   a) yes ❋
   b) can't tell
   c) no

4) Was the recruitment strategy appropriate to the aims of the research?
   a) yes ❋
   b) can't tell
   c) no

5) Was the data collected in a way that addressed the research issue?
   a) yes ❋
   b) can't tell
   c) no

6) Has the relationship between researcher and participants been adequately considered?
   a) yes ❋
   b) can't tell
   c) no

Section B: What are the results?

7) Have ethical issues been taken into consideration?
   a) yes ❋
   b) can't tell
   c) no

8) Was the data analysis sufficiently rigorous?
   a) yes ❋
   b) can't tell
   c) no

9) Is there a clear statement of findings?
   a) yes ❋
   b) can't tell
   c) no

Section C: Will the results help locally?

10) How valuable is the research?

Scores:[1,2,4,5]

Very good studies: 9-10 points } combined to low risk
Good studies: 7-8 points }
Satisfactory studies: 5-6 points moderate risk
Unsatisfactory studies: 0 to 4 points high risk

References

1. Wells G, Shea B, O'Connell D, et al. The Newcastle-Ottawa Scale (NOS) for assessing the quality of nonrandomised studies in meta-analyses. https://www.ohri.ca/programs/clinical_epidemiology/oxford.asp. Accessed March 13, 2024.
2. Yoo H, Kim JY, Lee YM, Kang M-Y. Occupational risk factors associated with lower urinary tract symptoms among female workers: a systematic review. Occup Environ Med. 2023;80(5):288-296. doi:10.1136/oemed-2022-108607.
3. Critical Appraisal Skills Programme. CASP Qualitative Studies Checklist. https://casp-uk.net/casp-tools-checklists/. Accessed March 13, 2024.
4. McWilliams S, Kinsella A, O'Callaghan E. Daily weather variables and affective disorder admissions to psychiatric hospitals. INTERNATIONAL JOURNAL OF BIOMETEOROLOGY. 2014;58(10):2045-2057. doi:10.1007/s00484-014-0805-9.
5. Nayebirad S, Mohamadi A, Yousefi-Koma H, et al. Association of anti-Ro52 autoantibody with interstitial lung disease in autoimmune diseases: a systematic review and meta-analysis. BMJ OPEN RESPIRATORY RESEARCH. 2023;10(1). doi:10.1136/bmjresp-2023-002076.

S4. Keywords and disease categorised according to the used outcome groups

Outcome group	Related Keywords and diseases
**Anxiety/ anxiety related**	Anxiety, anxiety disorder, climate anxiety, eco-anxiety, phobia
**Depression/ depression-related**	Depression, depressive episode, major depressive disorder, seasonal major depressive disorder, depressive mood swing, mood swings, mood change, episodic mood disorder
**Cognition**	Cognition, cognitive function, cognitive dysfunction, cognitive malfunction, cognitive impairment, dementia, cognitive deficit, learning problems
**Sleep**	Sleep, sleep comfort, sleep disorder, sleep disturbance, sleep duration, sleep quality, sleep relaxation, sleep stress, poor sleep, insomnia
**Stress/ stress-related**	Post-traumatic stress disorder, PTSD, post-traumatic stress, stress-related disease, stress disorder, post natural disaster trauma, emotional stress, psychological stress, physical stress
**Schizophrenia**	Schizophrenia, psychosis
**Emotions**	Solastalgia, happiness, grief, sorrow, sad, sadness, exhaustion, lost, lonely, loneliness, overwhelming, overwhelmed, panic, exhaustion, sense of safety, sense of security, fear, anger, frustration, guilt, powerless
**Quality of life**	Well-being, quality of life, emotional well-being, physical well-being, psychological well-being, mental disorder, mental health, mental illness, mental issue, mental problem. Mental strains, mental stress, mental weakness, mental wellness, psychological disorder, psychological illbeing, psychological illness, psychological issue, psychological issue, psychological problem, psychological stress, psychological wellbeing, psychological wellness, psychosocial disability, psychosocial disorder, psychosocial health psychosocial wellbeing, life satisfaction, psychological restoration, mental restoration, psychiatric disorder, psychiatric illness, psychiatric issue, psychiatric problem, heat stress
**Behaviour/ behaviour-related**	Behaviour disorder, behaviour problems, conduct disorder, hyperactivity, hypersensitive, attachment disorder, aggressive behaviour, adjustment problem

S5. Distribution and frequency of environmental and socio-individual determinants by outcome group for the hazards climate change, importance of the environment, flood, wildfire and meteorological variables and temperature extremes

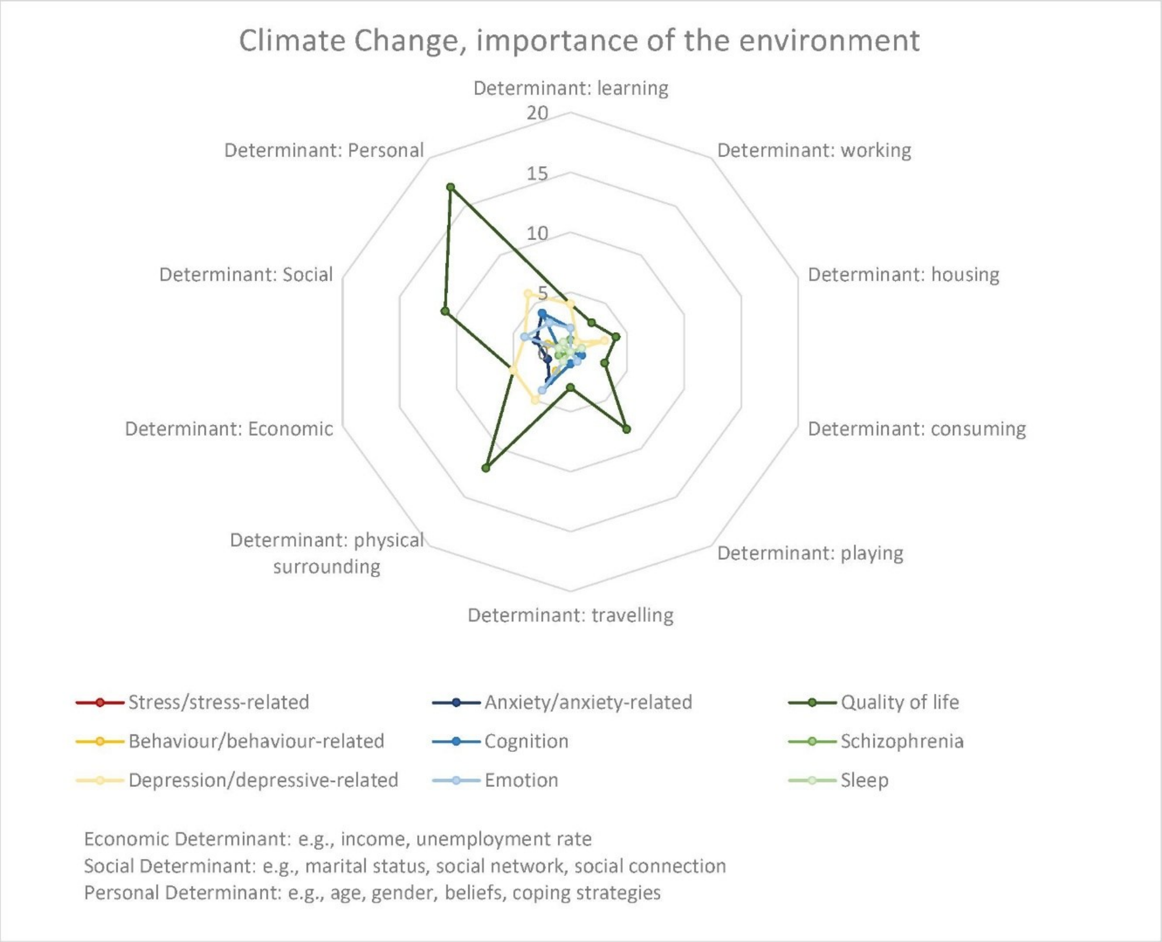

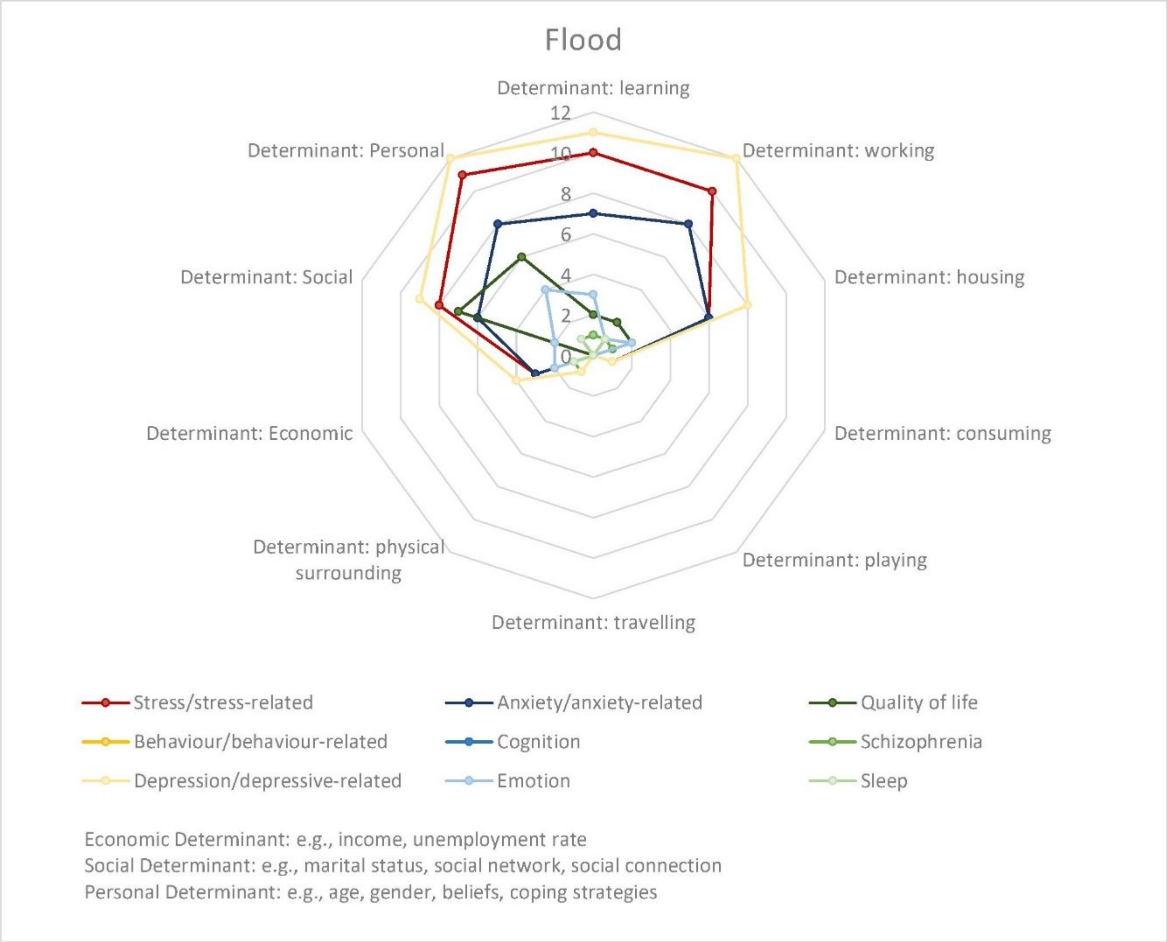

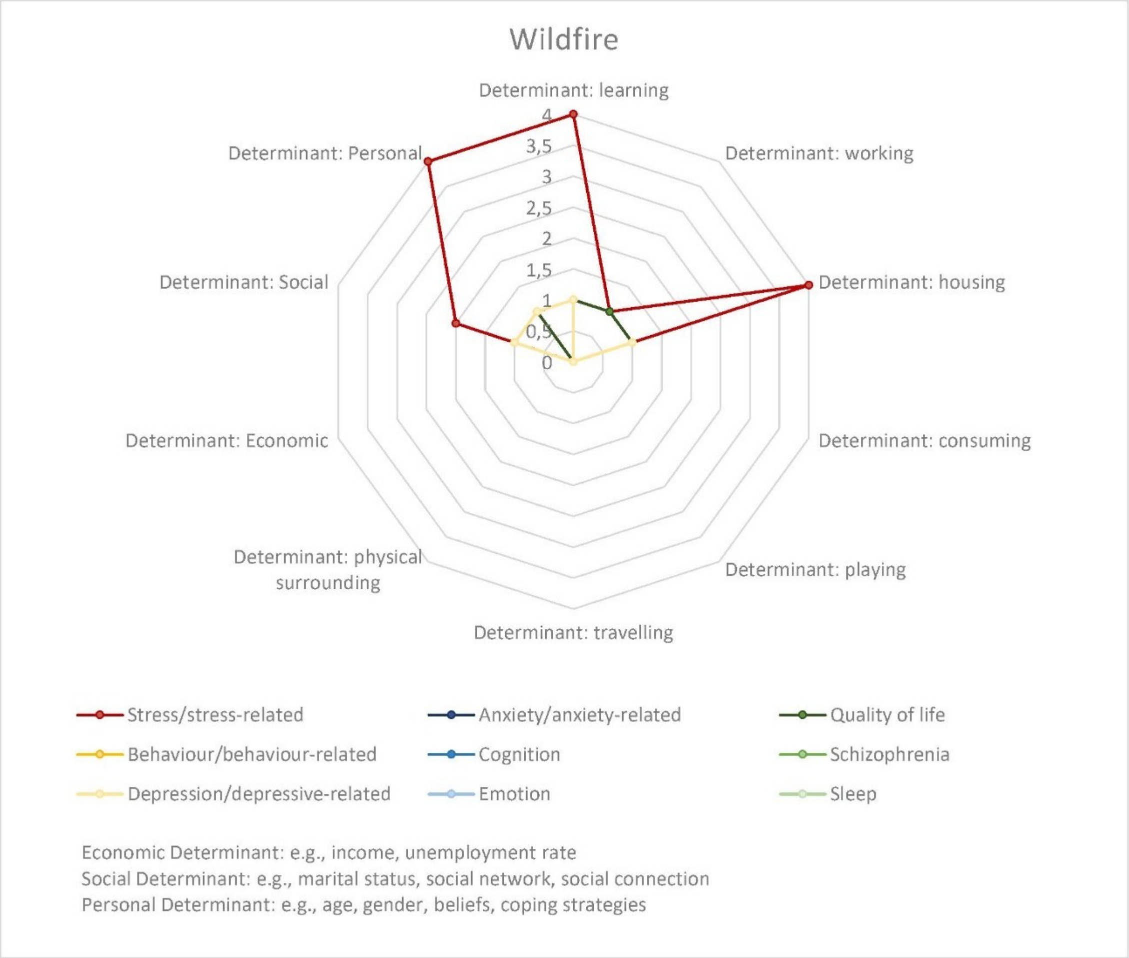

Wildfire
Determinant: learning
Determinant: working
Determinant: housing
Determinant: consuming
Determinant: playing
Determinant: travelling
Determinant: physical surrounding
Determinant: Economic
Determinant: Social
Determinant: Personal
0
0,5
1
1,5
2
2,5
3
3,5
4
Stress/stress-related
Anxiety/anxiety-related
Quality of life
Behaviour/behaviour-related
Cognition
Schizophrenia
Depression/depressive-related
Emotion
Sleep
Economic Determinant: e.g., income, unemployment rate
Social Determinant: e.g., marital status, social network, social connection
Personal Determinant: e.g., age, gender, beliefs, coping strategies

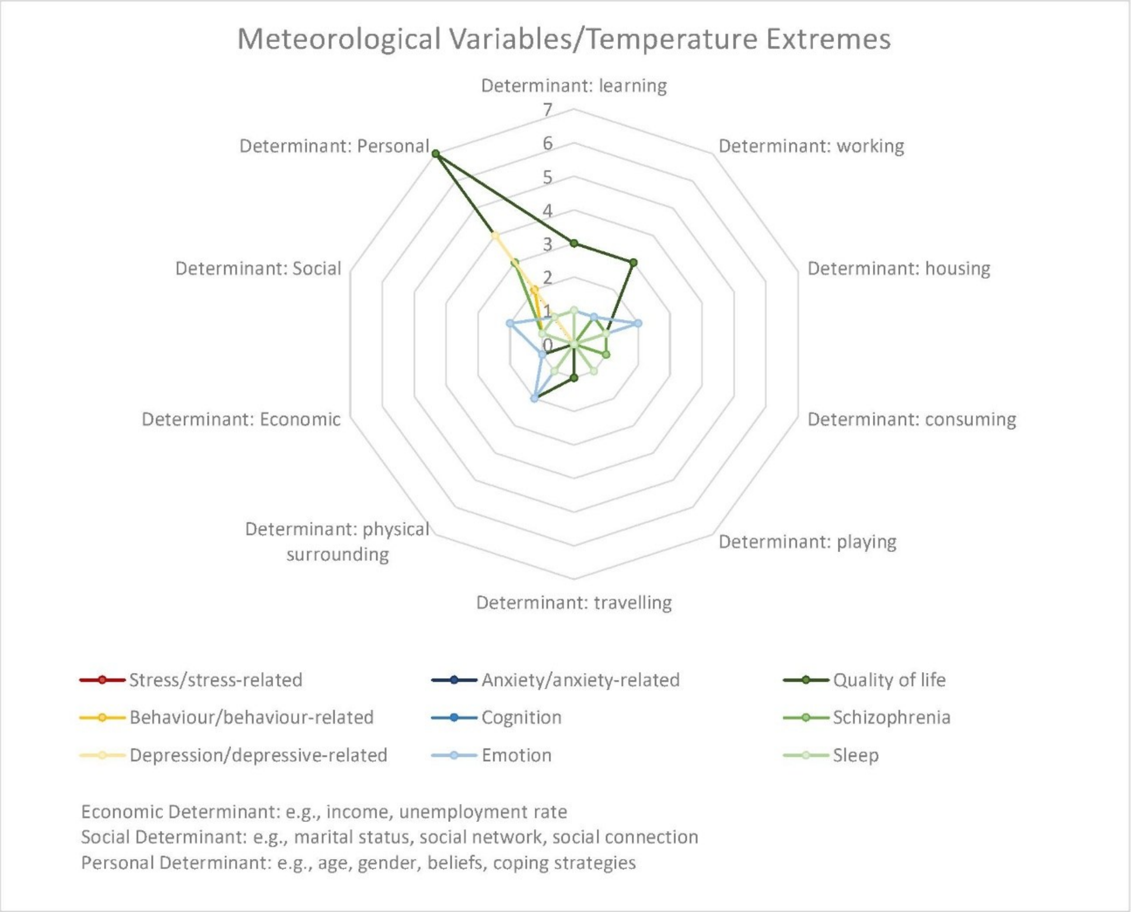

Meteorological Variables/Temperature Extremes
Determinant: learning
Determinant: working
Determinant: housing
Determinant: consuming
Determinant: playing
Determinant: travelling
Determinant: physical surrounding
Determinant: Economic
Determinant: Social
Determinant: Personal
0
1
2
3
4
5
6
7
Stress/stress-related
Anxiety/anxiety-related
Quality of life
Behaviour/behaviour-related
Cognition
Schizophrenia
Depression/depressive-related
Emotion
Sleep
Economic Determinant: e.g., income, unemployment rate
Social Determinant: e.g., marital status, social network, social connection
Personal Determinant: e.g., age, gender, beliefs, coping strategies

S6. Basic characteristics of the studies selected
(ROB: Risk of bias assessment)

Reference	Country	Study design	Target population	Sample size	Statistical Method	Hazard groups	Environmental determinants	Socio-individual determinants	Outcome Group	ROB
Abed et al. 2022	United Kingdom	Cohort/Longitudinal Study	Children: >16 years<br>Adults: all<br>Elderly: all	60,146	Regression Modelling: various regression models others: Pearson's correlation, Intraclass correlation coefficients (ICCs)	Air pollution	Education/learning<br>Employment/working<br>Recreation/playing	Economic<br>Social<br>Personal	Quality of life/well-being	low
Alemany et al. 2018	Spain	Cohort/Longitudinal Study	Children/adolescents: 7 - 11 years	1,667	Regression Modelling: Exponentiated regression coefficients	Air pollution	Education/learning	Personal	Cognition; Behaviour	medium
Andersen et al. 2022	Sweden, Denmark, France, the Netherlands, Germany, Austria	Cohort/Longitudinal Study	General population	271,720	Cox proportional hazards models; Kaplan-Meier curves; multiple linear regression models	Air pollution	Employment/working	Social<br>Personal	Depression<br>Anxiety<br>Cognition	low
Apergis 2018	Austria, Belgium, Bulgaria, Croatia, Denmark, Finland, France, Germany, Greece, Ireland, Italy, Luxembourg, Netherlands, Norway, Poland, Portugal, Romania, Slovakia, Slovenia, Spain, Sweden, Switzerland, United Kingdom	longitudinal study	general population	NA	Others: second-generation panel unit root, cross-sectional dependence (CD) statistic	Air pollution	Travelling/Mode of transportation<br>Consumption	Economic<br>Personal	Quality of life/well-being	low
Arbuthnott et al. 2020	United Kingdom	Longitudinal Study	general population	> million Greater London, >400,000 deaths in West Midlands and Greater Manchester	Regression Modelling: ecological time series regression	Temperature Extremes (heat wave/cold spell)	NA	Personal	Quality of life/well-being	low
Bakic and Ajdukovic 2019	Croatia	Longitudinal Study	Adults: 25 - 65 years	447	Regression Modelling: Single multivariate regression models; Others: latent difference score (LDS) models	Floods	Education/learning<br>Employment/working	Social<br>Personal	Depression;<br>Stress;<br>Quality of life/well-being	medium
Bakic et al. 2021	Croatia	Cross-sectional Study	Adults: 25 - 65 years	447	Others: structural equation modelling	Floods	Education/learning<br>Employment/working	Social<br>Personal	Stress;<br>Depression;<br>Quality of life/well-being	medium

Basagana et al. 2016	Spain	Cohort/Longitudinal Study	Children/adolescents: 7 - 10 years	2618	Others: linear mixed-effects models	Air pollution	Education/learning	Social<br>Personal	Cognition	medium
Benmarhnia et al. 2015	France	Cohort/Longitudinal Study	general population	3235	Regression Modelling: meta regression analysis; Others: random effect meta-analyses	meteorological variables	NA	Personal	Quality of life/well-being	low
Bettini et al 2021.	Italy	Qualitative Study	Sardinian mayors and local administrators	58	NA	Climate Change, predictions, emotions	Employment/working	NA	Emotion	low
Binter et al. 2022	United Kingdom, France, Spain, Greece	Cohort/Longitudinal Study	Children/adolescents: Birth till 4/5 years	5403	Regression Modelling: linear regressions	Climate Change and Environment;<br>Air pollution	Physical surrounding	Personal	Cognition	medium
Blennow et al. 2016	Sweden, Germany	Cross-sectional study	outdoor workers: forest owner<br>Adults	766	Regression Modelling: multinomial logistic regression	Climate Change, predictions, emotions	Education/learning	Personal	Emotions	medium
Bloemsma et al. 2022	the Netherlands	Cohort/Longitudinal Study	Children/adolescents: 11 - 18 years<br>Adults: 19 - 20 years	3059	Regression Modelling: three regression models	Climate Change and Environemt;<br>Air pollution	Physical surrounding	Personal	Quality of life/well-being	medium
de Boer et al. 2015	the Netherlands	cross-sectional study	Adults: >25 years<br>Elderly: <75 years	1682	Regression Modelling: regression method, multinomial logistic regression	Floods	Education/learning	Personal	Emotions	medium
Brereton et al. 2011	Ireland	Cross-sectional Study	20 – 65 years	3 focus groups 7-10 personen; postalsurvey of 300 individiduals; 812 Interviews in 2007;<br>656 Interviews in 2001	Regression Modelling	Climate Change and Environment	Education/learning<br>Employment/working	Economic<br>Social<br>Personal	Quality of life	medium
Brons et al. 2022	the Netherlands	Cross-sectional study	Children/adolescents: 12 - 16 years	6422	Regression Modelling: stratified adjusted regressions	Climate Change and Environment;<br>Air pollution	Physical surrounding<br>Education/learning	Social<br>Personal	Quality of life/well-being; Emotions; Behaviour	low

Bundo et al. 2021	Switzerland	Case-Cross over Study	general population	88996	Regression Modelling: conditional quasi-Poisson regression<br>Others: aggregated time-stratified case-crossover analysis	meteorological variables; Temperature Extremes (heat wave/cold spell)	NA	Personal	Stress; Depression; Anxiety; Schizophrenia; Cognition; Behaviour	low
Bunyan, Collins, Duffy 2016	United Kingdom	Cross-sectional Study	Children/adolescents: >16 years<br>Adults: all<br>Elderly: all	1005	Regression Modelling: multinomial regression<br>Others: logistic models	Climate Change, perceptions, emotions; Flood	Education/learning<br>Housing/Living	Economic<br>Social<br>Personal	Emotions	medium
Cerletti et al. 2021	Switzerland	Cross-sectional Study	Adults: >43 years<br>Elderly: <87 years	1980	Regression Modelling: multiple quantile regression models; multinomial (polytomous) logistic regression models	Climate Change and Environment	Physical surrounding<br>Education/learning<br>Employment/working<br>Housing/Living<br>Recreation/playing<br>Consumption	Social<br>Personal	Quality of life/well-being	low
Clark et al. 2012	United Kingdom	Cross-sectional Study	Children/adolescents: 9 -10 years	719	Regression Modelling: Multilevel linear and logistic regression models<br>Others: multilevel modelling	Air pollution	NA	Personal	Cognition; Quality of life/well-being	low
Cruz et al. 2022	United Kingdom	Cross-sectional Study	Children/adolescents: >18 years<br>Adults: all<br>Elderly: all	550918	Regression Modelling: Bayesian spatial regression model with random effects	Climate Change and Environment, Air pollution, Flood	Physical surrounding<br>Education/learning<br>Employment/working<br>Housing/Living	Economic<br>Personal	Schizophrenia; Depression	low
Cuesta et al. 2022	Sweden, Italy, Spain, Poland, France	cross-sectional study	Adults: >20 years<br>Elderly: <69 years	1014	Others: Pearson's chi-square; Wilcoxon rank sum test and Kruskal-Wallis (Dunn's test)	Wildfire; Temperature Extreme (heat wave/cold spell)	Education/learning<br>Employment/working<br>Housing/Living	Personal	Quality of life/well-being	medium
Cullen et al. 2018	United Kingdom	Cross-sectional Study<br>Cohort/longitudinal study	Adults: >40 years<br>Elderly: <69 years	86759	Regression Modelling: regression models, linear regression; logistic regression	Air pollution	Housing/Living<br>Recreation/playing	Personal	Cognition	Cross - low<br>Cohort - medium
Dadvand et al. 2015	Spain	Cohort/longitudinal study	Children/adolescents: 7 - 10 years	2593	Others: linear mixed effects models	Climate Change and Environment, Air pollution	Physical surrounding<br>Education/learning<br>Travelling/Mode of Transport<br>Consumption	Personal	Cognition	medium
Dadvand et al. 2018	Spain	Cohort/longitudinal study	Children/adolescents: 7 - 10 years	253	Regression Modelling: regression analysis<br>Others: statistical parametric maps (SPM8), linear mixed effects models	Climate Change and Environment	Physical surrounding	Personal	Cognition	medium

Davila et al. 2012	Spain	Cross-sectional Study	people with chronic diseases: adults with nasal polyposis	611	Others: frequencies and quantitative variables, centralization and dispersion measures, chi-squared test or a Fisher's exact test, Student's t-test or a Kruskall-Wallis, ANOVA test	Climate Change, perceptions, emotions	Consumption	Personal	Quality of life/well-being	low
Di Giorgi, Michielin and Michielin 2020	Italy	Qualitative study	others: asylum seeker	100	Regression Modelling: multiple regression analyses<br>Others: Student's t Index. effect size of the difference between the means was calculated using Cohen's d	Climate Change, perceptions, emotions	Education/learning<br>Housing/Living	Economic<br>Social<br>Personal	Anxiety;<br>Quality of life/well-being;<br>Emotions	medium
Dzhambov 2018	Bulgaria	cross-sectional, Cohort/Longitudinal Study	Children/adolescents: >= 18 years<br>Adults: <35 years	109	Others: cross-sectional single mediation models, cross- lagged panel mediation models	Climate Change and Environment, Air pollution	Physical surrounding<br>Recreation/playing	Social<br>Personal	Quality of life/well-being	Cross - medium<br>Cohort - high
Falcon et al. 2021	Spain	Cross-sectional Study	Adults: >45 years<br>Elderly: <74 years	212	Others: general linear models (GLM)	Air pollution	Physical surrounding<br>Education/learning	Personal	Cognition	low
Flachs et al. 2013	Denmark	Cohort/Longitudinal study	general population		Trend Analysis: multi state Markov model	Air pollution	NA	Economic<br>Personal	Quality of life/well-being	low
Fleming et al. 2015	United Kingdom, Sweden, Switzerland, the Netherlands, Denmark	Cohort/Longitudinal study	Children/adolescents: 1 - 17 years	282	Regression Modelling: logistic regression, general linear regression, Backwards stepwise regression<br>Others: multivariate general linear model	Climate Change and Environment	NA	Social<br>Personal	Quality of life/well-being	medium
Fleury-Bahi et al. 2023	Spain, the Netherlands, Portugal, Germany, France, Hungary	cross-sectional study	Children/adolescents: >17 years<br>Adults: all<br>Elderly: <73 years	1343	Regression Modelling: nested multilevel regression models	Climate Change and Environment	Physical surrounding	Personal	Quality of life/well-being	medium
Fonesca et al. 2020	Portugal	Case control Study	Children/adolescents: 8 - 17 years	483	Others: structural equation model, Multi-Sample Analysis, Latent Mean Comparison	Wildfire	Education/learning<br>Housing/Living	Personal	Stress	medium
Forns et al. 2016	Spain	Cross-sectional Study	Children/adolescents: 7 - 10 years	2897	Others: single-exposure models, multi-exposure models, Negative binomial mixed-effects models	Air pollution	Physical surrounding<br>Education/learning<br>Employment/working	Social<br>Personal	Behaviour	low
Forns et al. 2018	Denmark, the Netherlands, Germany, France, Italy, Spain, Sweden	Cohort/longitudinal study	Children/adolescents: 3 - 10 years	20127	Regression Modelling: logistic regression models	Air pollution	Physical surrounding<br>Housing/Living	Social<br>Personal	Behaviour	medium

Gao et al. 2022	United Kingdom	Cohort/longitudinal study	Adults: >37 yearsElderly: <73 years	363886	Regression Modelling: mixed- effect linear regression models<br>Others: MR analyses	Air pollution	Education/learning<br>Recreation/playing<br>Consumption	Personal	Sleep	medium
Gao et al. 2023	United Kingdom	Cohort/longitudinal study	Adults: >37<br>Elderly: <73 years	398241	Regression Modelling: logistic and Cox regression models	Air pollution	Education/learning<br>Housing/Living<br>Recreation/playing<br>Consumption	Personal	Depression	low
Gawrych and Holka-Pokorska 2022	Poland	Qualitative<br>Cross-sectional study	Adults: 37 - 41 years	72	descriptive analysis	Climate Change, perceptions, emotions	Employment/working<br>Housing/Living	Social<br>Personal	Emotions	low
Generaal et al. 2019	the Netherlands	Cross-sectional Study	general population	32487	Regression Modelling: Multilevel regression analyses	Climate Change and Environment, Air pollution	Physical surrounding<br>Education/learning<br>Housing/Living	Economic<br>Social<br>Personal	Depression	low
Generaal et al. 2018	the Netherlands	Cross-sectional Study	Adults: 18 - 65 years	2980	Regression Modelling: Multilevel logistic and linear regression analyses	Climate Change and Environment, Air pollution	Physical surrounding<br>Housing/Living	Economic<br>Social<br>Personal	Anxiety;<br>Depression	low
Gignac et al. 2022	Spain	Cross-sectional Study	Adults: all	288	Regression Modelling: Linear regression	Air pollution	Physical surrounding<br>Education/learning<br>Recreation/playing<br>Consumption	Personal	Cognition	medium
Giovanis and Ozdamar 2018	Austria, Belgium, Denmark, France, Germany, Greece, Italy, the Netherlands, Spain, Sweden, Switzerland	Cohort/Longitudinal Study	Adults: >50 yearsElderly: all	30792	Others: two-stage least squares, three stage least squares, Structural equation modelling	Air pollution	Physical surrounding<br>Education/learning<br>Housing/Living<br>Consumption	Economic<br>Social<br>Personal	Quality of life/well-being	medium
Gong et al. 2014	Sweden	Cohort/Longitudinal Study	Children/adolescents: 10,3 years +- 1,5 years	3426	Others: Generalized estimating equations (GEE) with exchangeable correlation structure	Air pollution	NA	Social<br>Personal	Behaviour	medium
Graham et al. 2019	United Kingdom	Cross-sectional Study	general population	7525	Regression Modelling: logistic regression	Floods	Education/learning<br>Employment/working<br>Housing/Living<br>Consumption	Economic<br>Personal	Stress;<br>Depression;<br>Anxiety	medium
Green et al. 2018	United Kingdom	Cross-sectional Study	general population		Regression Modelling: logistic regression model, linear regression modelOthers: Pearson Correlation, multi-scale analysis	Climate Change and Environment, Air pollution	Physical surrounding<br>Consumption	Social<br>Personal	Quality of life/well-being	medium
Grimm et al. 2012	Germany, United Kingdom, Spain, Poland, Czech Republic, Sweden	cross-sectional study	Adults: all	102	Others: regression, correlation, mancova, discriminant analysis and canonical correlation analysis	Floods	Education/learning	Personal	Stress	medium

Guxens et al. 2016	Sweden, the Netherlands, Italy, Spain	Cross-sectional Study	Children/adolescents: 3-10 years	8079	Regression Modelling: logistic regression models, negative binomial regression models	Air pollution	Physical surrounding	Personal	Behaviour	low
Guzman et al. 2022	Europe	Cohort/Longitudinal Study	general population	All European population	Others: non-linear exposure-response functions.	Air pollution	NA	Personal	Cognition	medium
Hart et al. 2018	Belgium, the Netherlands, United Kingdom, France, Hungary	Cross-sectional Study	general population	5205	Regression Modelling: multivariable multinomial logistic regression analyses, linear regression analyses	Climate Change and Environment	Physical surrounding<br>Employment/working<br>Housing/Living<br>Recreation/playing	Social<br>Personal	Quality of life/well-being	low
Heeren et al. 2022	France, Belgium, Switzerland	Cross-sectional Study	general population	2080	Others: t-tests, $\chi 2$ tests	Climate Change, perceptions, emotions	Education/learning	Personal	Anxiety	medium
Helbich et al. 2020	the Netherlands	Cross-sectional Study	Children/adolescents: >= 18 years<br>Adults: all	10482	Others: Chi2 tests, Kruskal-Wallis tests, Non-parametric Spearman correlation coefficients, generalized (geo)additive models (GAM)	Climate Change and Environment, Air pollution	Physical surrounding<br>Education/learning	Economic<br>Social<br>Personal	Depression	low
Hieronimi et al. 2023	Germany	Qualitative Study	Children/adolescents: all	9		Floods	Housing/Living: relocation	Social	Quality of life/well-being	medium
Hiscock et al. 2017	Finland	Cross-sectional Study	Adults: all<br>Elderly: all	782	Others: multivariable models	Climate Change and Environment, Air pollution	Physical surrounding<br>Housing/Living<br>Travelling/Mode of Transport<br>Recreation/playing	Social<br>Personal	Quality of life/well-being	medium
Huebner 2022	United Kingdom	Cross-sectional Study	Adults: 25 - 44 years	955 (UK), 950 (US)	Regression Modelling: ordinary least square regression, Poisson regression<br>Others: t-test	Climate Change, perceptions, emotions	Employment/working<br>Housing/Living	Social<br>Personal	Emotions	low
Hüls et al. 2018	Germany	Cohort/Longitudinal Study	Adults: >55 years<br>Elderly : <80 years	520	Regression Modelling: multiple linear regression analysis<br>Others: quasi-Bayesian Monte Carlo method	Air pollution	Housing/Living<br>Recreation/playing<br>Consumption	Personal	Cognition	medium
Janson et al. 2020	Norway, Sweden, Denmark, Estonia	Cross-sectional Study	others: born between 1945 and 1973	13578	Regression Modelling: Multilevel logistic regression<br>Others: $\chi 2$ test, t-test	Air pollution	Education/learning<br>Housing/Living<br>Recreation/playing<br>Consumption	Social<br>Personal	Sleep	medium
Jermacane et al. 2018	United Kingdom	Cohort/Longitudinal Study	Adults: all<br>others: people in municipalities affected by flooding	988	Regression Modelling: multivariable logistic regression model and conditional regression model	Floods	Education/learning<br>Working/Employment<br>Housing/living	Economic<br>Social<br>Personal	Anxiety;<br>Stress;<br>Depression	medium

Jorcano et al. 2019	the Netherlands, Germany, Poland, France, Italy, Spain	Cohort/Longitudinal Study	Children/adolescents: all	13182	Regression Modelling: Generalized additive models, logistic regression	Air pollution	Education/learning Consumption	Social Personal	Anxiety; Depression; Behaviour	medium
Karuga et al. 2022	Poland	Cross-sectional Study	general population	1240	Regression Modelling: Binary logistic regression Others: Mann–Whitney U test, Fisher's test, chi-square test	Climate Change, perceptions, emotions	Education/learning Employment/working Housing/Living	Economic Personal	Quality of life/well-being	medium
Klompmaker et al. 2019	the Netherlands	Cross-sectional Study	Adults: all Elderly: all	354827	Regression Modelling: logistic regression models	Air pollution	Physical surrounding Education/learning Recreation/playing Consumption	Economic Social Personal	Quality of life/well-being	low
Kriit et al. 2021	Sweden	Cohort/Longitudinal Study	Elderly: > 60 years people with chronic diseases: dementia	2650000	Others: Concentration response function (CRF), QALY measure, Total cost (TC) of dementia estimates	Air pollution	NA	Personal	Cognition	high
Kuhlicke et al. 2020	Germany	Cross-sectional Study	others: affected people	Sample 1: 1380 Sample 2: 1652	Regression Modelling: multiple linear regression analysis, logistic regression analysis Others: correlational analysis (Spearman correlations)	Floods	Housing/Living	Personal	Quality of life/well-being	medium
Lamond et al. 2015	United Kingdom	Cross-sectional Study	general population		Regression Modelling: binary logistic regression	Floods	Employment/working	Economic Personal	Anxiety; Stress; Depression; Sleep	medium
Lanki et al. 2017	Finland	Cross-sectional Study	Adults: 30-60 years	36	Others: mixed-models, t-test	Air pollution	Physical surrounding	Personal	Quality of life/well-being	medium
Latham et al. 2021	United Kingdom	Cohort/Longitudinal Study	Children/adolescents: age 10	2232	Regression Modelling: binary logistic Others: Huber-White variance estimator	Air pollution	Physical surrounding Education/learning Consumption	Social Personal	Depression	low
Lawrance et al. 2022	United Kingdom	Cross-sectional Study	Children/adolescents: all Adults: <24 years	530	Others: Wilcoxon's signed-rank test, Mann-Whitney U-tests or Kruskal-Wallis tests, Student's t tests, ANOVAs	Climate Change, perceptions, emotions	Recreation/playing	Personal	Quality of life/well-being	medium
Lemonsu et al. 2020	France	Cross-sectional Study	general population	185	Regression Modelling: linear regressions Others: principal component analyses (PCA)	meteorological variables; Air pollution	Physical surrounding	NA	Quality of life/well-being	high
Lima and Morais 2015	Portugal	Qualitative Study	general population	429	Others: basic descriptive statistics, Chi-square tests, t test	Climate Change, perceptions, emotions	Education/learning Employment/working	Economic Social	Quality of life/well-being; Emotions	medium
Lorenzoni et al. 2020	Germany, Austria	Cross-sectional study	general population	8	NA	Floods	NA	Social	Quality of life/well-being	high

Ma et al. 2023	United Kingdom	Cohort/Longitudinal Study	Elderly: 64,1 +2,8 years	164447	Regression Modelling: Cox proportional hazards regression, cubic spline analysis	Air pollution	Recreation/playing Consumption Education/learning	Personal	Cognition	low
Maitre et al. 2021	France, Greece, Lithuania, Norway, Spain, United Kingdom	Cohort/Longitudinal Study	Children/adolescents: all	1287	Regression Modelling: LASSO penalized negative binomial regression	Climate Change and Environment; Air pollution; meteorological variables	Physical surrounding Housing	Social	Emotions; Behaviour; Sleep	medium
Maran and Begotti 2021	Italy	Cross-sectional Study	Children/adolescents: >= 18 years Adults: <= 28 years	312	Others: Correlations	Climate Change, perceptions, emotions	NA	Social Personal	Anxiety	medium
McWilliams et al. 2013	Ireland	Cohort/Longitudinal Study	Adults: all	48347	Regression Modelling: ARIMA and timeseries regression analysis Others: Kruskal-Wallis Test	meteorological variables	Employment/working Consumption	Social personal	Schizophrenia	low
McWilliams et al. 2014	Ireland	Cohort/Longitudinal Study	others: hospitalizations for mania or depression	34465	Regression Modelling: Kruskal-Wallis, multiple linear time-series regression	meteorological variables	NA	Personal	Depression	low
Messeri et al. 2019	Italy	Cross-sectional Study	workers at agricultural (vinyard, citrus farm) and construction sides	104	ANOVA	Temperature extremes (heat wave/cold spell)	Education/learning	Economic Personal	Quality of life	medium
Midouhas et al. 2019	United Kingdom	Cohort/Longitudinal Study	Children/adolescents: 9 months - 3 years	11625	Regression Modelling: 2-level linear regression models	Air pollution	Physical surrounding Education/learning Housing/Living	Economic Personal	Behaviour; Emotions	low
Mikutta et al. 2022	Switzerland	Cohort/Longitudinal Study	general population	182	Others: distributed lag model, unconstrained distributed lag linear model generalized linear models (GLM) with conditional quasi-Poisson regression, Student's t-tests	meteorological variables	NA	Personal	Depression	low
Mort et al. 2018	United Kingdom	Qualitative Study	Children/adolescents: 6 - 15 years	41	NA	Floods	Housing/Living	Social	Quality of life/well-being	low
Mortamais et al. 2021	France	Cohort/Longitudinal Study	Elderly: all	7066	Others: Multilevel spatial random-effects Cox proportional hazards models	Air pollution	Education/learning	Personal	Cognition	low
Mortamais et al. 2019	Spain	Cohort/Longitudinal Study	Children/adolescents: 8 - 11 years	186	Regression Modelling: separate linear regression models, negative binomial regression (for behavioural)	Air pollution	Education/learning	Personal	Cognition; Behaviour	low

Mortamais et al. 2017	Spain	Cohort/Longitudinal Study	Children/adolescents: 8 - 12 years	242	Regression Modelling: negative binomial mixed regression models	Air pollution	NA	Personal	Behaviour	low
Motreff et al. 2013	France	Cross-sectional study	general population	20982	Others: generalized additive model (GAM), Poisson distribution	Floods	NA	Personal	Quality of life/well-being	medium
Mukadam et al. 2022	United Kingdom	Cohort/Longitudinal Study	Adults: 55 - 64 years<br>Elderly: 65 - 69 years	294162	Regression Modelling: Cox regression	Air pollution	Education/learning<br>Recreation/playing	Social<br>Personal	Cognition	medium
Mulchandani et al. 2020	United Kingdom	Cohort/longitudinal study	general population	569	Regression Modelling: Crude logistic regression models, Multivariable logistic regression models, Conditional logistic regression<br>Others: Wald test	Floods	Education/learning<br>Employment/working<br>Housing/Living	Social<br>Personal	Stress;<br>Anxiety;<br>Depression	medium
Munzo-Cano et al. 2018	Spain	Cohort/Longitudinal Study	Adults: all<br>people with chronic diseases: allergic rhinitis	670	Regression Modelling: multiple lineal regression model, logistic regression<br>Others: Fisher's exact test, Student's t test, ANOVA test, multivariate analysis, Tuckey test	Climate Change and Environment	NA	Personal	Anxiety;<br>Sleep;<br>Quality of life/well-being;<br>Depression	medium
Munro et al. 2017	United Kingdom	Cross-sectional Study	Children/adolescents: >= 18 years<br>Adults: all<br>Elderly: all	622	Regression Modelling: ordinal logistic regression analyses	Floods	Education/learning<br>Employment/working<br>Housing/Living	Social<br>Personal	Depression;<br>Anxiety;<br>Stress	medium
Mutz et al. 2021	United Kingdom	Cross-sectional Study, Longitudinal Study	Adults: >37 yearsElderly: <73 years	307378	Regression Modelling: Logistic regression analyses, Ordinal logistic regression analyses	Air pollution	Physical surrounding<br>Education/learning<br>Recreation/playing	Economic<br>Social<br>Personal	Quality of life/well-being	Cross - low<br>Cohort - medium
Newbury et al. 2022	United Kingdom	Cohort/Longitudinal Study	Children/adolescents: all	2232	Regression Modelling: ordinal logistic regression	Air pollution; Climate Change, perceptions, emotions	Physical surrounding	Economic<br>Social<br>personal	Depression;<br>Schizophrenia	low
Nußbaum et al. 2020	Germany	Cohort/Longitudinal Study	Elderly: 55-85 years	4,814/<br>MRI analysis: 615	Regression Modelling: Multiple linear regression models	Air pollution	Education/learning<br>Recreation/playing	Economic<br>Personal	Coginition	medium
O'Neill et al. 2016	Ireland	Cross-sectional Study	General population in flood risk area	305	Regression Modelling	Floods	Education/learning<br>Employment/working<br>Housing/living	Economic<br>Social<br>Personal	Emotions	medium
Orru et al. 2012	Lithuania	Cohort/Longitudinal Study	Adults: all<br>Elderly: all	555 733 Vilnius and 355 586 Kaunas	NA	Air pollution	NA	Personal	Quality of life/well-being	medium

Orru et al. 2022	Finland, Norway, Denmark, Sweden	Cohort/Longitudinal Study	Adults: >30 years	1883902	Others: concentration-response functions (CRF)	Air pollution	Physical surrounding Housing/Living	Personal	Quality of life/well-being	medium
Orru et al. 2022	Finland, Estonia	Cross-sectional Study	Adults: > 25 yearsElderly: < 74 years	1112 Finland, 918 Estonia	Regression Modelling: logistic regression analysis	Air pollution	Education/learning Employment/working Travelling/Mode of Transport	Economic Social Personal	Emotions	medium
Orru et al. 2016	Estonia	Cross-sectional Study	general population	3770	Regression Modelling: regression analysis	Air pollution	Employment/working	Economic Personal	Quality of life/well-being	medium
Oudin et al. 2018	Sweden	Case-Cross over Study	general population	NA	Regression Modelling: Poisson regression model Others: multi-pollutant models	Air pollution	NA	NA	Quality of life/well-being	medium
Oudin et al. 2016	Sweden	Cohort/Longitudinal Study	Children/adolescents: <18 years	552221	Regression Modelling: Cox regression	Air pollution	NA	Economic Personal	Schizophrenia Anxiety Depression	medium
Oudin et al. 2017	Sweden	Cohort/Longitudinal Study	Children/adolescents: <18 years	745171	Regression Modelling: logistic regression	Air pollution	NA	Personal	Quality of life/well-being	medium
Oudin et al. 2017	Sweden	Cohort/Longitudinal Study	Elderly: 60 - 85 years	1469	Others: Generalized Estimating Equations	Air pollution	Education/learning Employment/working Recreation/playing Consumption	Social Personal	Cognition	medium
Oudin et al. 2019	Sweden	Cohort/Longitudinal Study	Children/adolescents: 1 - 17 years	48571	Regression Modelling: logistic regression	Air pollution	NA	Personal	Cognition; Behaviour	medium
Page et al. 2012	United Kingdom	Cohort/Longitudinal Study	general population	22562	Regression Modelling: Poisson regression models	meteorological variables	Housing/Living	Personal	Schizophrenia; Cognition	medium
Papadatou et al. 2012	Greece	Cross-sectional Study	Children/adolescents: 12 - 17 years	1468	Regression Modelling: Poisson regression model Others: Poisson multilevel models; multiple imputation technique	Wildfire	Education/learning Housing/Living	Social Personal	Stress; Depression	medium
Peisker 2023	Bulgaria, Croatia, Czech Republic, Estonia, Latvia, Lithuania, Poland, Romania, Slovakia, Slovenia, Denmark, Finland, Ireland, Sweden, United Kingdom, Greece, Italy, Portugal, Spain, Austria, Belgium, France, Germany, Luxembourg, the Netherlands	Cross-sectional Study	Children/adolescents: >15 yearsAdults: all Elderly: <99 years	455931	Others: Bayesian model averaging (BMA)	meteorological variables	Physical surrounding Education/learning Employment/working Housing/Living	Economic Social Personal	Emotions	medium
Pelgrim et al. 2021	Belgium	Cross-sectional Study	Children/adolescents: >15 years Adults: all Elderly: all	1325	Regression Modelling: multi-exposure regression models	Air pollution	Physical surrounding Education/learning Recreation/playing	Economic Social Personal	Depression; Anxiety, Sleep	low

Pereira et al. 2021	Portugal	Cross-sectional Study	Children/adolescents: 8 - 17 years	486	Others: correlation analysis	Wildfire	Education/learning Housing/Living	Personal	Stress	medium
Petrowski et al. 2019	Germany	Cross-sectional Study	Children/adolescents: >=18 years Adults: all Elderly: 64-91 years	746	Regression Modelling: Stepwise multiple linear regressions	Air pollution	Education/learning Employment/working Housing/Living	Economic Social Personal	Stress	medium
Petrowski et al. 2021	Germany	Cross-sectional Study	Children/adolescents: >=18 years Adults: all Elderly: 64-92 years	3020	Regression Modelling: Multivariate linear regressionsOthers: mean values, standard deviations, and frequencies	Air pollution	NA	Economic Personal	Quality of life/well-being	medium
Pirkle et al. 2022	United Kingdom	Qualitative Study	others: Members of the House of Common and the House of Lords	41	speech analysis	Climate Change, perceptions, emotions	NA	NA	Quality of life/well-being	medium
Pogacar et al. 2018	Slovenia	Cross-sectional Study	Working people	400	NA	Temperature extremes (heat wave/cold spell)	NA	Personal	Quality of life	medium
Pogacar et al. 2019	Slovenia, Greece	Cross-sectional Study	Adults: >20 years Elderly: all outdoor workers: workers in agriculture, tourism, construction	286 (216 from Slovenia and 70 from Greece)	Others: Pearson Chi-square tests, Independent Samples t-tests	Temperature Extremes (heat wave/cold spell)	Education/learning Employment/working	Personal	Quality of life/well-being	medium
Rahman et al. 2022	Sweden	Cohort/Longitudinal Study	Children/adolescents: >15 years Adults: all	4673764	Others: chi-squared tests, Cox's proportional hazard models	Climate Change, perceptions, emotions	Education/learning Housing/Living	Economic Social Personal	Stress	low
Rakotozandry et al. 2019	France	Cohort/Longitudinal Study	Adults: all (mean (SD) age 41.7 (13))	47	Others: autoregressive correlation matrix	Climate Change and Environment		Personal	Quality of life/well-being	medium
Rathmann et al. 2020	Germany	Cross-sectional Study	others: students	21	Others: non-parametric Kruskal-Wallis tests, pairwise Wilcoxon tests	Climate Change and Environment; meteorological variables	Physical surrounding Travelling/Mode of Transport	Personal	Quality of life/well-being	high
Razieh et al. 2019	United Kingdom	Cohort/Longitudinal Study	Children/adolescents: >18 yearsAdults: all Elderly: <75 years	1760	Others: generalized estimating equation model with an exchangeable correlation structure, post hoc generalized estimating equation models	Air pollution	Physical surrounding Recreation/playing Consumption	Personal	Depression; Anxiety	medium
Reuben et al. 2021	United Kingdom	Cohort/Longitudinal Study	Children/adolescents: 5 - 18 years	2039	Regression Modelling: ordinary least squares multiple linear regression	Air pollution	Physical surrounding	Social Personal	Stress, Behaviour, Anxiety, Depression	medium

Rittner et al. 2020	Sweden	Cohort/Longitudinal Study	Children/adolescents: >5 years<br>Adults: all<br>Elderly: all	1247993	Others: dispersion modelling (Aermod-based implementation), C–R relationships	Air pollution	NA	Personal	Quality of life/well-being	medium
Ritz et al. 2018	Denmark	Case-Control Study	Children/adolescents: all	83526	Regression Modelling: conditional logistic regression<br>Others: conditional logistic models	Air pollution	Housing/Living	Personal	Cognition	low
Rivas et al. 2019	Spain	Cohort/Longitudinal Study	Children/adolescents: 7 - 10 years	2221	Others: linear mixed effects models, distributed lag models (DLM)	Air pollution	Housing/Living	Social<br>Personal	Cognition	medium
Roberts et al. 2019	United Kingdom	Cohort/Longitudinal Study	Children/adolescents: 5 - 18 years	284	Regression Modelling: Linear regression, Binary logistic regression<br>Others: cross-sectional analysis	Air pollution	NA	Social<br>Personal	Depression; Behaviour; Anxiety	low
Robin C et al. 2020	United Kingdom	Cross-sectional Study	Adults: all<br>Elderly: all	957	Regression Modelling: Linear regression with robust SEs	Floods	NA	Social<br>Personal	Quality of life/well-being	medium
Rocha K. et al. 2012	Spain	Cross-sectional Study	Children/adolescents:> 16<br>Adults: all	23760	Regression Modelling: multivariate logistic regression models	Climate Change, perceptions, emotions	NA	Economic<br>Personal	Anxiety; Depression	low
Ronaldson et al. 2022	United Kingdom	Cross-sectional Study	Adults: 40 - 64 years<br>Elderly: 65 - 69 years	364144	Regression Modelling: ordinal regression model, logistic regression models	Air pollution	Physical surrounding<br>Recreation/playing<br>Consumption	Economic<br>Personal	Depression; Anxiety	low
Russ et al. 2021	United Kingdom	Cohort/Longitudinal Study	Children/adolescents:> 11<br>Adults: all<br>Elderly: < 79 years	572	Regression Modelling: linear regression models<br>Others: latent growth models	Air pollution	Consumption	Personal	Cognition	medium
Sauliene et al. 2016	Lithuania	Cohort/Longitudinal Study	Adults: all<br>Elderly: < 77 years	665	Others: Box–Whisker method, Chi- square criterion, t test	Climate Change and Environment	Recreation/playing	Personal	Quality of life/well-being	medium
Scibor and Malinowska-Cieslik 2020	Poland	Cohort/Longitudinal Study	Adults: 20 - 64 years<br>Elderly: 65 - 80 years	300	Regression Modelling: linear regression models	Air pollution	Education/learning<br>Employment/working<br>Consumption<br>Housing/Living	Personal	quality of life	medium
Shiue 2015	Europe	Cross-sectional Study	general population	79270	Regression Modelling: logistic regression modelling	Air pollution	Education/learning<br>Housing/Living	Economic<br>Social<br>Personal	Quality of life/well-being	medium
Silverforsen et al. 2021	Sweden	Cross-sectional Study	Children/adolescents: >16 years<br>Adults: all<br>Elderly: <75 years	25848	Regression Modelling: Multiple logistic regression<br>Others: $\chi 2$ test and unpaired t-test	Air pollution	Education/learning<br>Recreation/playing<br>Consumption	Personal	Sleep	medium
Sommar et al. 2014	Sweden	Cross-sectional Study	Children/adolescents: >16 years<br>Adults: all<br>Elderly: <76 years	941	Others: Kruskal-Wallis rank sum test, Wilcoxon signed-rank test, $\chi 2$ tests, ANOVA, Spearman's correlation coefficient	Air pollution	NA	Personal	Quality of life/well-being	medium

Spence et al. 2012	United Kingdom	Cross-sectional Study	Children/adolescents: >= 15 years<br>Adults: all<br>Elderly: all	1822	Regression Modelling: linear regression models	Climate Change, perceptions, emotions	Employment/working	Social personal	Quality of life/well-being	medium
Sunyer at al. 2015	Spain	Cohort/Longitudinal Study	Children/adolescents: 7 - 10 years	2715	Regression Modelling: Linear mixed effects modelling	Air pollution	NA	Personal	Cognition	medium
Tempest et al. 2017	United Kingdom	Cross-sectional Study	Children/adolescents: >18 years<br>Adults: all<br>Elderly: all	2126	Regression Modelling: crude logistic regression modelling	Floods	Education/learning<br>Employment/working<br>Housing/Living	Social<br>Personal	Depression;<br>Anxiety;<br>Stress	medium
Terpstra 2011	the Netherlands	Cross-sectional Study	general population	Study 1: 472;<br>Study 2: 1289	Others: structural equation modelling (SEM)	Floods	NA	Personal	Emotions	medium
Tharrey et al. 2020	France	Cohort/Longitudinal Study	Adults: all	132	Others: t-test for continuous variables, McNemar test for categorical variables, linear mixed-effect models, logistic mixed-effect models	Climate Change and Environment	Physical surrounding<br>Education/learning<br>Travelling/Mode of Transport<br>Recreation/playing<br>Consumption	Economic<br>Social<br>Personal	Quality of life/well-being	medium
Theleritis et al. 2020	Greece	Cross-sectional Study	outdoor workers: figherfighter	102	Regression Modelling: Multiple logistic regression analysisOthers: Student's t-tests, receiver operating characteristic (ROC) curves, area under the curve (AUC)	Wildfire	Education/learning<br>Employment/working<br>Housing/Living	Social<br>Personal	Stress	medium
Thieken et al. 2016	Germany	Cross-sectional Study	general population	1652	NA	Floods	Housing/Living	NA	Quality of life/well-being	medium
Thomas et al. 2022	France	Qualitative Study	Children/adolescents: 7 - 18 years	35	NA	Climate Change, perceptions, emotions	Education/learning	Personal	Emotions	medium
Tonne et al. 2014	United Kingdom	Cohort/Longitudinal Study	general population	2867	Regression Modelling: linear regression models	Air pollution	Education/learning<br>Employment/working<br>Recreation/playing<br>Consumption	Social<br>Personal	Cognition	medium
Triebner et al. 2022	Norway, Sweden	Cross-sectional Study	Adults: all	1069	Regression Modelling: logistic regression models, negative binomial regression models	Climate Change and Environment	Physical surrounding<br>Recreation/playing	Personal	Quality of life/well-being	medium
Triguero-Mas et al. 2017	Spain	Case-Cross over Study	Adults: all	26	Regression Modelling: multilevel mixed-effects linear regression models	Climate Change and Environment	Physical surrounding<br>Education/learning<br>Recreation/playing	Social<br>Personal	Stress;<br>Anxiety;<br>Emotions;<br>Cognition	medium
van den Berg et al. 2015	the Netherlands	Cross-sectional Study	Adults: all	3817	Regression Modelling: Linear regression<br>Others: Pearson’s correlation, t-test	Air pollution;<br>Climate Change and Environment	Physical surrounding	Social<br>Personal	Emotions	medium

Verheyen et al. 2021	Belgium	Cohort/Longitudinal Study	Adults: allothers: pregnant women	149	Regression Modelling: linear regression models Others: Spearman ran correlations	Air pollution	Physical surrounding Consumption	Personal	Stress	medium
Waite et al. 2017	United Kingdom	Cross-sectional Study	general population	2126	Regression Modelling: logistic regression	Floods	Education/learning Employment/working Housing/Living	Social Personal	Depression; Stress; Anxiety	medium
Walker-Springett et al. 2017	United Kingdom	Cohort/Longitudinal Study	Adults: all	qualitative: 60, survey: 1000	Regression Modelling: stepwise multiple regression analysis	Floods	Housing/Living	Social Personal	Quality of life/well-being	medium
Ward Thompson et al. 2014	United Kingdom	Longitudinal Study, Cross-section Study	Elderly: all	96	Regression Modelling: hierarchical blocked linear regressions Others: Differences & degree of change over time; maximum likelihood method (t-test)	Climate Change and Environment	Physical surrounding Housing/Living Recreation/playing	Personal	Quality of life/well-being	Cross – medium Cohort - medium
Weierstall-Pu st et al. 2022	Germany	Cross-sectional Study	Adults: all Elderly: all	3094	Regression Modelling: linear regression analyses (backwards exclusion) Others: U-test, Friedman test statistics, Wilcoxon tests, Cook's distances, variance inflation factors	Climate Change, perceptions, emotions	NA	Personal	Stress	medium
Weinreich et al. 2015	Germany	Cohort/Longitudinal Study	Adults: 50 - 64 years Elderly: 65 - 80 years	1773	Regression Modelling: logistic and linear regression models Others: spearman correlation	Air pollution; meteorological variables	Education/learning Recreation/playing	Personal	Sleep	low
Wind et al. 2011	United Kingdom	Cross-sectional Study	Adults: all Elderly: all	231	Regression Modelling: regression and path analysis	Floods	Education/learning Employment/working	Social Personal	Anxiety; Stress; Depression	medium
Wind et al. 2021	United Kingdom	Cross-sectional Study	Adults: all Elderly: all	231	Others: Multilevel structural equation modelling	Floods	Education/learning Employment/working Housing/Living	Social Personal	Depression	medium
Zare Sakhvidi et al. 2022	France	Cross-sectional Study	Adults: all Elderly: 65 - 69 years	123754	Regression Modelling: regression incidence rate ratio, regression with restricted cubic spline functions	Air pollution	Education/learning Recreation/playing Housing/Living	Economic Personal	Depression	low
Zaremba et al. 2022	Poland	Qualitative Study	Adults: all Elderly: <82 years	40	NA	Climate Change, perceptions, emotions	Education/learning Housing/Living	Social Personal	Emotions	low
Zock et al. 2018	the Netherlands	Cross-sectional Study	general population	4450	Regression Modelling: multilevel mixed effects logistic regression, Others: non-parametric Spearman's correlation coefficients	Climate Change and Environment; Air pollution	Physical surrounding	Economic Social Personal	Anxiety; Depression	low

References

Abed Al Ahad, M., Demsar, U., Sullivan, F. & Kulu, H. (2022). Air pollution and individuals' mental well-being in the adult population in United Kingdom: A spatial-temporal longitudinal study and the moderating effect of ethnicity. *PLOS ONE*, *17*(3), e0264394. https://doi.org/10.1371/journal.pone.0264394

Alemany, S., Vilor-Tejedor, N., García-Esteban, R., Bustamante, M., Dadvand, Payam, Esnaola, M [Mikel], Mortamais, M [Marion], Forns, J [Joan], van Drooge, B., Álvarez-Pedrerol, M., Grimalt, J., Rivas, I [Ioar], Querol, X [Xavier], Pujol, J [Jesus] & Sunyer, J [Jordi] (2018). Traffic-Related Air Pollution, APOEepsilon4 Status, and Neurodevelopmental Outcomes among School Children Enrolled in the BREATHE Project (Catalonia, Spain). *ENVIRONMENTAL HEALTH PERSPECTIVES*, *126*(8), 87001. https://doi.org/10.1289/EHP2246

Andersen, Z. J., Zhang, J., Jørgensen, J. T., Samoli, E., Liu, S., Chen, J [Jie], Strak, M [Maciej], Wolf, K., Weinmayr, G., Rodopolou, S., Remfry, E., Hoogh, K. de [Kees], Bellander, T., Brandt, J., Concin, H., Zitt, E., Fecht, D., Forastiere, F., Gulliver, J., . . . Lim, Y.-H. (2022). Long-term exposure to air pollution and mortality from dementia, psychiatric disorders, and suicide in a large pooled European cohort: ELAPSE study. *ENVIRONMENT INTERNATIONAL*, *170*, 107581. https://doi.org/10.1016/j.envint.2022.107581

Apergis, N. (2018). The Impact of Greenhouse Gas Emissions on Personal Well-Being: Evidence from a Panel of 58 Countries and Aggregate and Regional Country Samples. *JOURNAL OF HAPPINESS STUDIES*, *19*(1), 69–80. https://doi.org/10.1007/s10902-016-9809-y

Arbuthnott, K., Hajat, S., Heaviside, C. & Vardoulakis, S. (2020). Years of life lost and mortality due to heat and cold in the three largest English cities. *ENVIRONMENT INTERNATIONAL*, *144*, 105966. https://doi.org/10.1016/j.envint.2020.105966

Bakic, H. & Ajdukovic, D. (2019). Stability and change post-disaster: dynamic relations between individual, interpersonal and community resources and psychosocial functioning. *European Journal of Psychotraumatology*, *10*(1). https://doi.org/10.1080/20008198.2019.1614821

Bakic, H. & Ajdukovic, D. (2021). Resilience after natural disasters: the process of harnessing resources in communities differentially exposed to a flood. *European Journal of Psychotraumatology*, *12*(1). https://doi.org/10.1080/20008198.2021.1891733

Basagaña, X [X.], Esnaola, M [M.], Rivas, I [I.], Amato, F., Alvarez-Pedrerol, M [M.], Forns, J [J.], Lopez-Vicente, M., Pujol, J [J.], Nieuwenhuijsen, M [M.], Querol, X [X.] & Sunyer, J [J.] (2016). Neurodevelopmental deceleration by urban fine particles from different emission sources: A longitudinal observational study. *ENVIRONMENTAL HEALTH PERSPECTIVES*, *124*(10), 1630–1636. https://doi.org/10.1289/EHP209

Benmarhnia, T [Tarik], Grenier, P., Brand, Allan, Fournier, M., Deguen, S. & Smargiassi, A. (2015). Quantifying Vulnerability to Extreme Heat in Time Series Analyses: A Novel Approach Applied to Neighborhood Social Disparities under Climate Change. *International journal of environmental research and public health*, *12*(9), 11869–11879. https://doi.org/10.3390/ijerph120911869

Bettini, G., Beuret, N. & Turhan, E. (2021). On the Frontlines of Fear. Vorab-Onlinepublikation. https://doi.org/10.14288/acme.v20i3.1838 (322-340 Pages / ACME: An International Journal for Critical Geographies, Vol. 20 No. 3 (2021): Vol 20, No 3: Themed Section: "Moral Economies" & Various Articles).

Binter, A.-C., Bernard, J., Mon-Williams, M., Andiarena, A., González-Safont, L., Vafeiadi, M., Lepeule, J., Soler-Blasco, R., Alonso, L., Kampouri, M., Mceachan, R., Santa-Marina, L., Wright, J., Chatzi, L., Sunyer, J [Jordi], Philippat, C., Nieuwenhuijsen, M., Vrijheid, M. & Guxens, M [Mònica] (2022). Urban environment and cognitive and motor function in children from four European birth cohorts. *ENVIRONMENT INTERNATIONAL*, *158*, 106933. https://doi.org/10.1016/j.envint.2021.106933

Blennow, K., Persson, J., Persson, E. & Hanewinkel, M. (2016). Forest Owners' Response to Climate Change: University Education Trumps Value Profile. *PLOS ONE*, *11*(5), e0155137. https://doi.org/10.1371/journal.pone.0155137

Bloemsma, L [L.], Wijga, A [A.], Klompmaker, J [J.], Hoek, G [G.], Janssen, N [N.], Lebret, E [E.], Brunekreef, B [B.] & Gehring, U [U.] (2022). Green space, air pollution, traffic noise and mental wellbeing throughout adolescence: Findings from the PIAMA study. *ENVIRONMENT INTERNATIONAL*, *163*. https://doi.org/10.1016/j.envint.2022.107197

Boer, J. de, Botzen, W. J. W. & Terpstra, T. (2015). More Than Fear Induction: Toward an Understanding of People's Motivation to Be Well-Prepared for Emergencies in Flood-Prone Areas. *RISK ANALYSIS*, *35*(3), 518–535. https://doi.org/10.1111/risa.12289

Brereton, F., Bullock, C., Clinch, J. P. & Scott, M. (2011). Rural change and individual well-being. *European Urban and Regional Studies*, *18*(2), 203–227. https://doi.org/10.1177/0969776411399346

Brons, M., Bolt, G., Helbich, M., Visser, K. & Stevens, G. (2022). Independent associations between residential neighbourhood and school characteristics and adolescent mental health in the Netherlands. *HEALTH & PLACE*, *74*, 102765. https://doi.org/10.1016/j.healthplace.2022.102765

Bundo, M., Schrijver, E. de, Federspiel, A., Toreti, A., Xoplaki, E., Luterbacher, J., Franco, O., Müller, T. & Vicedo-Cabrera, A. M. (2021). Ambient temperature and mental health hospitalizations in Bern, Switzerland: A 45-year time-series study. *PLOS ONE*, *16*(10), e0258302. https://doi.org/10.1371/journal.pone.0258302

Bunyan, S., Collins, A. & Duffy, D. (2016). Concern and Helplessness: Citizens' Assessments of Individual and Collective Action on the Provision of Environmental Public Goods in a Coastal City at Risk of Inundation. *ENVIRONMENTAL MANAGEMENT*, *58*(3), 431–445. https://doi.org/10.1007/s00267-016-0730-2

Cerletti, P., Eze, I., Keidel, D., Schaffner, E., Stolz, D., Gasche-Soccal, P. M., Rothe, T., Imboden, M. & Probst-

Hensch, N. (2021). Perceived built environment, health-related quality of life and health care utilization. *PLOS ONE*, *16*(5), e0251251. https://doi.org/10.1371/journal.pone.0251251

Clark, C., Crombie, R., Head, J., van Kamp, I., van Kempen, E. & Stansfeld, S. (2012). Does traffic-related air pollution explain associations of aircraft and road traffic noise exposure on children's health and cognition? A secondary analysis of the United Kingdom sample from the RANCH project. *American journal of epidemiology*, *176*(4), 327–337. https://doi.org/10.1093/aje/kws012

Cruz, J., Li, G., Aragon, M. J., Coventry, P., Jacobs, R., Prady, S. & White, P. (2022). Association of environmental and socioeconomic indicators with serious mental illness diagnoses identified from general practitioner practice data in England: A spatial Bayesian modelling study. *PLOS MEDICINE*, *19*(6), e1004043. https://doi.org/10.1371/journal.pmed.1004043

Cuesta, A., Alvear, D., Carnevale, A. & Amon, F. (2022). Gender and Public Perception of Disasters: A Multiple Hazards Exploratory Study of EU Citizens. *SAFETY*, *8*(3). https://doi.org/10.3390/safety8030059

Cullen, B., Newby, D., Lee, D., Lyall, D., Nevado-Holgado, A., Evans, J., Pell, J., Lovestone, S. & Cavanagh, J. (2018). Cross-sectional and longitudinal analyses of outdoor air pollution exposure and cognitive function in UK Biobank. *SCIENTIFIC REPORTS*, *8*(1), 12089. https://doi.org/10.1038/s41598-018-30568-6

Dadvand, P., Nieuwenhuijsen, M., Esnaola, M [Mikel], Forns, J [Joan], Basagaña, X [Xavier], Alvarez-Pedrerol, M [Mar], Rivas, I [Ioar], López-Vicente, M [Monica], Castro Pascual, M. de, Su, J., Jerrett, M., Querol, X [Xavier] & Sunyer, J [Jordi] (2015). Green spaces and cognitive development in primary schoolchildren. *PROCEEDINGS OF THE NATIONAL ACADEMY OF SCIENCES OF THE UNITED STATES OF AMERICA*, *112*(26), 7937–7942. https://doi.org/10.1073/pnas.1503402112

Dadvand, P., Pujol, J [Jesus], Macià, D., Martínez-Vilavella, G., Blanco-Hinojo, L., Mortamais, M [Marion], Alvarez-Pedrerol, M [Mar], Fenoll, R., Esnaola, M [Mikel], Dalmau-Bueno, A., López-Vicente, M [Monica], Basagaña, X [Xavier], Jerrett, M., Nieuwenhuijsen, M. J. & Sunyer, J [Jordi] (2018). The Association between Lifelong Greenspace Exposure and 3-Dimensional Brain Magnetic Resonance Imaging in Barcelona Schoolchildren. *ENVIRONMENTAL HEALTH PERSPECTIVES*, *126*(2), 27012. https://doi.org/10.1289/EHP1876

Dávila, I., Rondón, C., Navarro, A., Antón, E., Colás, C., Dordal, M. T., Ibáñez, M. D., Fernández-Parra, B., Lluch-Bernal, M., Matheu, V., Montoro, J., Sánchez, M. C. & Valero, A [Antonio] (2012). Aeroallergen sensitization influences quality of life and comorbidities in patients with nasal polyposis. *American journal of rhinology & allergy*, *26*(5), e126-31. https://doi.org/10.2500/ajra.2012.26.3792

Di Giorgi, E., Michielin, P. & Michielin, D. (2020). Perception of climate change, loss of social capital and mental health in two groups of migrants from African countries. *Annali dell'Istituto superiore di sanita*, *56*(2), 150–156. https://doi.org/10.4415/ANN_20_02_04

Dzhambov, A. (2018). Residential green and blue space associated with better mental health: a pilot follow-up study in university students. *Arhiv za higijenu rada i toksikologiju*, *69*(4), 340–349. https://doi.org/10.2478/aiht-2018-69-3166

Falcón, C., Gascon, M., Molinuevo, J. L., Operto, G., Cirach, M [M.], Gotsens, X., Fauria, K., Arenaza-Urquijo, E., Pujol, J [J.], Sunyer, J [J.], Nieuwenhuijsen, M [M.], Gispert, J. & Crous-Bou, M. (2021). Brain correlates of urban environmental exposures in cognitively unimpaired individuals at increased risk for Alzheimer's disease: A study on Barcelona's population. *Alzheimer's and Dementia: Diagnosis, Assessment and Disease Monitoring*, *13*(1), e12205. https://doi.org/10.1002/dad2.12205

Flachs, E. M., Sørensen, J., Bønløkke, J. & Brønnum-Hansen, H. (2013). Population dynamics and air pollution: the impact of demographics on health impact assessment of air pollution. *Journal of environmental and public health*, *2013*, 760259. https://doi.org/10.1155/2013/760259

Fleming, L., Murray, C., Bansal, A., Hashimoto, S., Bisgaard, H., Bush, A., Frey, U., Hedlin, G., Singer, F., van Aalderen, W., Vissing, N., Zolkipli, Z., Selby, A., Folwer, S., Shaw, D., Chung, K. F., Sousa, A., Wagers, S., Corfield, J., . . . Roberts, G. (2015). The burden of severe asthma in childhood and adolescence: results from the paediatric U-BIOPRED cohorts. *EUROPEAN RESPIRATORY JOURNAL*, *46*(5), 1322–1333. https://doi.org/10.1183/13993003.00780-2015

Fleury-Bahi, G., Galharret, J.-M., Lemée, C., Wittenberg, I., Olivos, P., Loureiro, A., Jeuken, Y., Laïlle, P. & Navarro, O. (2023). Nature and well-being in seven European cities: The moderating effect of connectedness to nature. *APPLIED PSYCHOLOGY-HEALTH AND WELL BEING*, *15*(2), 479–498. https://doi.org/10.1111/aphw.12390

Fonseca, A., Vagos, P., Moreira, H., Pereira, J., Canavarro, M. C. & Rijo, D. (2020). Psychometric Properties of the Portuguese Version of the Child Post-traumatic Cognitions Inventory in a Sample of Children and Adolescents Following a Wildfire Disaster. *Child psychiatry and human development*, *51*(6), 876–887. https://doi.org/10.1007/s10578-020-00965-y

Forns, J [Joan], Dadvand, P., Foraster, M., Alvarez-Pedrerol, M [Mar], Rivas, I [Ioar], López-Vicente, M [Monica], Suades-Gonzalez, E., Garcia-Esteban, R., Esnaola, M [Mikel], Cirach, M [Marta], Grellier, J., Basagaña, X [Xavier], Querol, X [Xavier], Guxens, M [Mònica], Nieuwenhuijsen, M. & Sunyer, J [Jordi] (2016). Traffic-Related Air Pollution, Noise at School, and Behavioral Problems in Barcelona Schoolchildren: A Cross-Sectional Study. *ENVIRONMENTAL HEALTH PERSPECTIVES*, *124*(4), 529–535. https://doi.org/10.1289/ehp.1409449

Forns, J [Joan], Sunyer, J [Jordi], Garcia-Esteban, R., Porta, D., Ghassabian, A., Giorgis-Allemand, Lise, Gong, T., Gehring, U [Ulrike], Sørensen, M., Standl, M., Sugiri, D [Dorothee], Almqvist, C., Andiarena, A., Badaloní, C., Beelen, R., Berdel, D., Cesaroni, G., Charles, M.-A., . . . Guxens, M [Mònica] (2018). Air

Pollution Exposure During Pregnancy and Symptoms of Attention Deficit and Hyperactivity Disorder in Children in Europe. *EPIDEMIOLOGY*, *29*(5), 618–626. https://doi.org/10.1097/EDE.0000000000000874

Gao, X., Huang, N., Guo, X. & Huang, T. (2022). Role of sleep quality in the acceleration of biological aging and its potential for preventive interaction on air pollution insults: Findings from the UK Biobank cohort. *Aging cell*, *21*(5), e13610. https://doi.org/10.1111/acel.13610

Gao, X., Jiang, M., Huang, N., Guo, X. & Huang, T. (2023). Long-Term Air Pollution, Genetic Susceptibility, and the Risk of Depression and Anxiety: A Prospective Study in the UK Biobank Cohort. *ENVIRONMENTAL HEALTH PERSPECTIVES*, *131*(1), 17002. https://doi.org/10.1289/EHP10391

Gawrych, M. & Holka-Pokorska, J. (2022). Mental health issues related to climate change in Poland - Polish psychologists' and psychotherapists' perspective. *Archives of Psychiatry and Psychotherapy*, *24*(2), 47–53. https://doi.org/10.12740/APP/142826

Generaal, E., Hoogendijk, E., Stam, M., Henke, C., Rutters, F., Oosterman, M., Huisman, M., Kramer, S., Elders, P., Timmermans, E., Lakerveld, J., Koomen, E., Have, M. ten, Graaf, R. de, Snijder, M., Stronks, K., Willemsen, G., Boomsma, D., Smit, J. & Penninx, B. (2019). Neighbourhood characteristics and prevalence and severity of depression: pooled analysis of eight Dutch cohort studies. *The British journal of psychiatry : the journal of mental science*, *215*(2), 468–475. https://doi.org/10.1192/bjp.2019.100

Generaal, E., Timmermans, E., Dekkers, J., Smit, J. & Penninx, B. (2018). Not urbanization level but socioeconomic, physical and social neighbourhood characteristics are associated with presence and severity of depressive and anxiety disorders. *Psychological medicine*, *49*(1), 149–161. https://doi.org/10.1017/S0033291718000612

Gignac, F., Righi, V., Toran, R., Paz Errandonea, L., Ortiz, R., Mijling, B., Naranjo, A., Nieuwenhuijsen, M., Creus, J. & Basagaña, X [Xavier] (2022). Short-term NO2 exposure and cognitive and mental health: A panel study based on a citizen science project in Barcelona, Spain. *ENVIRONMENT INTERNATIONAL*, *164*, 107284. https://doi.org/10.1016/j.envint.2022.107284

Giovanis, E. & Ozdamar, O. (2018). Health status, mental health and air quality: evidence from pensioners in Europe. *ENVIRONMENTAL SCIENCE AND POLLUTION RESEARCH*, *25*(14), 14206–14225. https://doi.org/10.1007/s11356-018-1534-0

Gong, T., Almqvist, C., Bölte, S., Lichtenstein, P., Anckarsäter, H., Lind, T., Lundholm, C. & Pershagen, G. (2014). Exposure to air pollution from traffic and neurodevelopmental disorders in Swedish twins. *Twin research and human genetics : the official journal of the International Society for Twin Studies*, *17*(6), 553–562. https://doi.org/10.1017/thg.2014.58

Graham, H., White, P., Cotton, J. & McManus, S. (2019). Flood- and Weather-Damaged Homes and Mental Health: An Analysis Using England's Mental Health Survey. *International journal of environmental research and public health*, *16*(18). https://doi.org/10.3390/ijerph16183256

Green, M. A., Daras, K., Davies, A., Barr, B. & Singleton, A. (2018). Developing an openly accessible multi-dimensional small area index of 'Access to Healthy Assets and Hazards' for Great Britain, 2016. *HEALTH & PLACE*, *54*, 11–19. https://doi.org/10.1016/j.healthplace.2018.08.019

Grimm, A., Hulse, L., Preiss, M. & Schmidt, S. (2012). Post- and peritraumatic stress in disaster survivors: an explorative study about the influence of individual and event characteristics across different types of disasters. *European Journal of Psychotraumatology*, *3*. https://doi.org/10.3402/ejpt.v3i0.7382

Guxens, M [Mònica], Ghassabian, A., Gong, T., Garcia-Esteban, R., Porta, D., Giorgis-Allemand, Lise, Almqvist, C., Aranbarri, A., Beelen, R., Badaloni, C., Cesaroni, G., Nazelle, A. de, Estarlich, M., Forastiere, F., Forns, J [Joan], Gehring, U [Ulrike], Ibarluzea, J., Jaddoe, V., . . . Sunyer, J [Jordi] (2016). Air Pollution Exposure during Pregnancy and Childhood Autistic Traits in Four European Population-Based Cohort Studies: The ESCAPE Project. *ENVIRONMENTAL HEALTH PERSPECTIVES*, *124*(1), 133–140. https://doi.org/10.1289/ehp.1408483

Guzmán, P., Tarín-Carrasco, P., Morales-Suárez-Varela, M. & Jiménez-Guerrero, P. (2022). Effects of air pollution on dementia over Europe for present and future climate change scenarios. *ENVIRONMENTAL RESEARCH*, *204*. https://doi.org/10.1016/j.envres.2021.112012

Hart, E. A. C., Lakerveld, J., McKee, M., Oppert, J.-M., Rutter, H., Charreire, H., Veenhoven, R., Bárdos, H., Compernolle, S., Bourdeaudhuij, I. de, Brug, J. & Mackenbach, J. D. (2018). Contextual correlates of happiness in European adults. *PLOS ONE*, *13*(1), e0190387. https://doi.org/10.1371/journal.pone.0190387

Heeren, A., Mouguiama-Daouda, C. & Contreras, A. (2022). On climate anxiety and the threat it may pose to daily life functioning and adaptation: a study among European and African French-speaking participants. *CLIMATIC CHANGE*, *173*(1). https://doi.org/10.1007/s10584-022-03402-2

Helbich, M., Browning, M. & Huss, A. (2020). Outdoor light at night, air pollution and depressive symptoms: A cross-sectional study in the Netherlands. *SCIENCE OF THE TOTAL ENVIRONMENT*, *744*, 140914. https://doi.org/10.1016/j.scitotenv.2020.140914

Hieronimi, A., Elbel, J., Schneider, M., Wermuth, I., Schulte-Körne, G., Nowak, D. & Bose-O'Reilly, S. (2023). A Qualitative Study to Explain the Factors Influencing Mental Health after a Flooding. *International journal of environmental research and public health*, *20*(1). https://doi.org/10.3390/ijerph20010134

Hiscock, R., Asikainen, A., Tuomisto, J., Jantunen, M., Pärjälä, E. & Sabel, C. (2017). City scale climate change policies: Do they matter for wellbeing? *Preventive Medicine Reports*, *6*, 265–270. https://doi.org/10.1016/j.pmedr.2017.03.019

Huebner, G. (2022). The role of parenthood in worry about overheating in homes in the UK and the US and implications for energy use: An online survey study. *PLOS ONE*, *17*(12), e0277286. https://doi.org/10.1371/journal.pone.0277286

Hüls, A., Vierkötter, A., Sugiri, D [Dorothea], Abramson, M. J., Ranft, U., Krämer, U. & Schikowski, T. (2018). The role of air pollution and lung function in cognitive impairment. *EUROPEAN RESPIRATORY JOURNAL*, *51*(2). https://doi.org/10.1183/13993003.01963-2017

Janson, E., Johannessen, A., Holm, M., Franklin, K., Holst, G. J., Gislason, T., Jögi, R., Lindberg, E., Svartengren, M. & Janson, C [Christer] (2020). Insomnia associated with traffic noise and proximity to traffic-a cross-sectional study of the Respiratory Health in Northern Europe III population. *JOURNAL OF CLINICAL SLEEP MEDICINE*, *16*(4), 545–552. https://doi.org/10.5664/jcsm.8274

Jermacane, D., Waite, T. D [Thomas David], Beck, C., Bone, A [Angie], Amlôt, R., Reacher, M., Kovats, S., Armstrong, B [Ben], Leonardi, G [Giovanni], James Rubin, G. & Oliver, I [Isabel] (2018). The English National Cohort Study of Flooding and Health: the change in the prevalence of psychological morbidity at year two. *BMC PUBLIC HEALTH*, *18*(1), 330. https://doi.org/10.1186/s12889-018-5236-9

Jorcano, A., Lubczynska, M., Pierotti, L., Altug, H., Ballester, F., Cesaroni, G., El Marroun, H., Fernández-Somoano, A., Freire, C., Hanke, W., Hoek, G [Gerard], Ibarluzea, J., Iñiguez, C., Jansen, P., Lepeule, J., Markevych, I., Polanska, K., Porta, D., Schikowski, T., . . . Guxens, M [Mònica] (2019). Prenatal and postnatal exposure to air pollution and emotional and aggressive symptoms in children from 8 European birth cohorts. *ENVIRONMENT INTERNATIONAL*, *131*, 104927. https://doi.org/10.1016/j.envint.2019.104927

Karuga, F. F., Szmyd, B., Petroniec, K., Walter, Aleksandra Pawelczyk, Agnieszka, Sochal, M., Bialasiewicz, P., Strzelecki, D., Respondek-Liberska, M., Tadros-Zins, M. & Gabryelska, A. (2022). The Causes and Role of Antinatalism in Poland in the Context of Climate Change, Obstetric Care, and Mental Health. *International journal of environmental research and public health*, *19*(20). https://doi.org/10.3390/ijerph192013575

Klompmaker, J [Jochem], Hoek, G [Gerard], Bloemsma, L [Lizan], Wijga, A [Alet], van den Brink, C., Brunekreef, B [Bert], Lebret, E [Erik], Gehring, U [Ulrike] & Janssen, N [Nicole] (2019). Associations of combined exposures to surrounding green, air pollution and traffic noise on mental health. *ENVIRONMENT INTERNATIONAL*, *129*, 525–537. https://doi.org/10.1016/j.envint.2019.05.040

Kriit, H. K., Forsberg, B [Bertil], Aström, D. O. & Oudin, A. (2021). Annual dementia incidence and monetary burden attributable to fine particulate matter (PM2.5) exposure in Sweden. *ENVIRONMENTAL HEALTH*, *20*(1), 65. https://doi.org/10.1186/s12940-021-00750-x

Kuhlicke, C., Masson, T., Kienzler, S., Sieg, T., Thieken, A. & Kreibich, H. (2020). Multiple Flood Experiences and Social Resilience: Findings from Three Surveys on Households and Companies Exposed to the 2013 Flood in Germany. *WEATHER CLIMATE AND SOCIETY*, *12*(1), 63–88. https://doi.org/10.1175/WCAS-D-18-0069.1

Lamond, J. E., Joseph, R. & Proverbs, D. (2015). An exploration of factors affecting the long term psychological impact and deterioration of mental health in flooded households. *ENVIRONMENTAL RESEARCH*, *140*, 325–334. https://doi.org/10.1016/j.envres.2015.04.008

Lanki, T [Timo], Siponen, T., Ojala, A., Korpela, K., Pennanen, A., Tiittanen, P [Pekka], Tsunetsugu, Y., Kagawa, T. & Tyrväinen, L. (2017). Acute effects of visits to urban green environments on cardiovascular physiology in women: A field experiment. *ENVIRONMENTAL RESEARCH*, *159*, 176–185. https://doi.org/10.1016/j.envres.2017.07.039

Latham, R., Kieling, C., Arseneault, L [Louise], Botter-Maio Rocha, T., Beddows, A., Beevers, S., Danese, A., Oliveira, K. de, Kohrt, B., Moffitt, T [Terrie], Mondelli, V., Newbury, J [Joanne], Reuben, A. & Fisher, H [Helen] (2021). Childhood exposure to ambient air pollution and predicting individual risk of depression onset in UK adolescents. *Journal of psychiatric research*, *138*, 60–67. https://doi.org/10.1016/j.jpsychires.2021.03.042

Lawrance, E., Jennings, N., Kioupi, V., Thompson, R., Diffey, J. & Vercammen, A. (2022). Psychological responses, mental health, and sense of agency for the dual challenges of climate change and the COVID-19 pandemic in young people in the UK: an online survey study. *LANCET PLANETARY HEALTH*, *6*(9), e726-e738. https://doi.org/10.1016/S2542-5196(22)00172-3

Lemonsu, A., Amossé, A., Chouillou, D., Gaudio, N., Haouès-Jouve, S., Hidalgo, J., Le Bras, J., Legain, D., Marchandise, S. & Tudoux, B. (2020). Comparison of microclimate measurements and perceptions as part of a global evaluation of environmental quality at neighbourhood scale. *INTERNATIONAL JOURNAL OF BIOMETEOROLOGY*, *64*(2), 265–276. https://doi.org/10.1007/s00484-019-01686-1

Lima, M. & Morais, R. (2015). Lay perceptions of health and environmental inequalities and their associations to mental health. *CADERNOS DE SAUDE PUBLICA*, *31*(11), 2342–2352. https://doi.org/10.1590/0102-311X00105714

Lorenzoni, N., Stühlinger, V., Stummer, H. & Raich, M. (2020). Long-Term Impact of Disasters on the Public Health System: A Multi-Case Analysis. *International journal of environmental research and public health*, *17*(17). https://doi.org/10.3390/ijerph17176251

Ma, H., Li, X., Zhou, T., Wang, M., Heianza, Y. & Qi, L. (2023). Long-term exposure to low-level air pollution, genetic susceptibility and risk of dementia. *INTERNATIONAL JOURNAL OF EPIDEMIOLOGY*, *52*(3), 738–748. https://doi.org/10.1093/ije/dyac146

Maitre, L., Julvez, J., López-Vicente, M [Monica], Warembourg, C., Tamayo-Uria, I., Philippat, C., Gützkow, K., Guxens, M [Monica], Andrusaityte, S., Basagaña, X [Xavier], Casas, M., Castro, M. de, Chatzi, L., Evandt, J., Gonzalez, J., Grazuleviciene, R., Smastuen Haug, L., Heude, B., Hernandez-Ferrer, C., . . . Vrijheid, M. (2021). Early-life environmental exposure determinants of child behavior in Europe: A longitudinal, population-based study. *ENVIRONMENT INTERNATIONAL*, *153*, 106523. https://doi.org/10.1016/j.envint.2021.106523

Maran, D. A. & Begotti, T. (2021). Media Exposure to Climate Change, Anxiety, and Efficacy Beliefs in a Sample of

Italian University Students. *International journal of environmental research and public health*, *18*(17). https://doi.org/10.3390/ijerph18179358
McWilliams, S., Kinsella, A. & O'Callaghan, E. (2013). The effects of daily weather variables on psychosis admissions to psychiatric hospitals. *INTERNATIONAL JOURNAL OF BIOMETEOROLOGY*, *57*(4), 497–508. https://doi.org/10.1007/s00484-012-0575-1
McWilliams, S., Kinsella, A. & O'Callaghan, E. (2014). Daily weather variables and affective disorder admissions to psychiatric hospitals. *INTERNATIONAL JOURNAL OF BIOMETEOROLOGY*, *58*(10), 2045–2057. https://doi.org/10.1007/s00484-014-0805-9
Messeri, A., Morabito, M., Bonafede, M., Bugani, M., Levi, M., Baldasseroni, A., Binazzi, A., Gozzini, B., Orlandini, S., Nybo, L. & Marinaccio, A. (2019). Heat Stress Perception among Native and Migrant Workers in Italian Industries-Case Studies from the Construction and Agricultural Sectors. *International journal of environmental research and public health*, *16*(7). https://doi.org/10.3390/ijerph16071090
Midouhas, E., Kokosi, T. & Flouri, E. (2019). The quality of air outside and inside the home: associations with emotional and behavioural problem scores in early childhood. *BMC PUBLIC HEALTH*, *19*(1), 406. https://doi.org/10.1186/s12889-019-6733-1
Mikutta, C., Pervilhac, C., Znoj, H., Federspiel, A. & Müller, T. (2022). The Impact of Foehn Wind on Mental Distress among Patients in a Swiss Psychiatric Hospital. *International journal of environmental research and public health*, *19*(17). https://doi.org/10.3390/ijerph191710831
Mort, M., Walker, M., Williams, A. L. & Bingley, A. (2018). Displacement: Critical insights from flood-affected children. *HEALTH & PLACE*, *52*, 148–154. https://doi.org/10.1016/j.healthplace.2018.05.006
Mortamais, M [M.], Gutierrez, L., Hoogh, K. de [K.], Chen, J [J.], Vienneau, D [D.], Carrière, I., Letellier, N., Helmer, C., Gabelle, A., Mura, T., Sunyer, J [J.], Benmarhnia, T [T.], Jacquemin, B [B.] & Berr, C [C.] (2021). Long-term exposure to ambient air pollution and risk of dementia: Results of the prospective Three-City Study. *ENVIRONMENT INTERNATIONAL*, *148*. https://doi.org/10.1016/j.envint.2020.106376
Mortamais, M [M.], Pujol, J [Jesus], Martínez-Vilavella, G., Fenoll, R., Reynes, C., Sabatier, R., Rivas, I [Ioar], Forns, J [Joan], Vilor-Tejedor, N., Alemany, S., Cirach, M [Marta], Alvarez-Pedrerol, M [Mar], Nieuwenhuijsen, M. & Sunyer, J [Jordi] (2019). Effects of prenatal exposure to particulate matter air pollution on corpus callosum and behavioral problems in children. *ENVIRONMENTAL RESEARCH*, *178*, 108734. https://doi.org/10.1016/j.envres.2019.108734
Mortamais, M [M.], Pujol, J [Jesus], van Drooge, B. L., Macià, D., Martínez-Vilavella, G., Reynes, C., Sabatier, R., Rivas, I [Ioar], Grimalt, J., Forns, J [Joan], Alvarez-Pedrerol, M [Mar], Querol, X [Xavier] & Sunyer, J [Jordi] (2017). Effect of exposure to polycyclic aromatic hydrocarbons on basal ganglia and attention-deficit hyperactivity disorder symptoms in primary school children. *ENVIRONMENT INTERNATIONAL*, *105*, 12–19. https://doi.org/10.1016/j.envint.2017.04.011
Motreff, Y., Pirard, P., Goria, S., Labrador, B., Gourier-Fréry, C., Nicolau, J., Le Tertre, A. & Chan-Chee, C. (2013). Increase in psychotropic drug deliveries after the Xynthia storm, France, 2010. *Prehospital and disaster medicine*, *28*(5), 428–433. https://doi.org/10.1017/S1049023X13008662
Mukadam, N., Marston, L., Lewis, G. & Livingston, G. (2022). Risk factors, ethnicity and dementia: A UK Biobank prospective cohort study of White, South Asian and Black participants. *PLOS ONE*, *17*(10). https://doi.org/10.1371/journal.pone.0275309
Mulchandani, R., Armstrong, B [Ben], Beck, C., Waite, T. D [Thomas David], Amlot, R [Richard], Kovats, S., Leonardi, G [Giovanni], Rubin, G. J. & Oliver, I [Isabel] (2020). The English National Cohort Study of Flooding & Health: psychological morbidity at three years of follow up. *BMC PUBLIC HEALTH*, *20*(1), 321. https://doi.org/10.1186/s12889-020-8424-3
Muñoz-Cano, R., Ribó, P., Araujo, G., Giralt, E., Sanchez-Lopez, J. & Valero, A [A.] (2018). Severity of allergic rhinitis impacts sleep and anxiety: Results from a large Spanish cohort. *CLINICAL AND TRANSLATIONAL ALLERGY*, *8*(1), 23. https://doi.org/10.1186/s13601-018-0212-0
Munro, A., Kovats, R., Rubin, G., Waite, T. D [T. D.], Bone, A [A.], Armstrong, B [B.], Beck, C [C.], Amlot, R [R.], Leonardi, G [G.] & Oliver, I [I.] (2017). Effect of evacuation and displacement on the association between flooding and mental health outcomes: a cross-sectional analysis of UK survey data. *LANCET PLANETARY HEALTH*, *1*(4), e134-e141. https://doi.org/10.1016/S2542-5196(17)30047-5
Mutz, J., Roscoe, C. J. & Lewis, C. M. (2021). Exploring health in the UK Biobank: associations with sociodemographic characteristics, psychosocial factors, lifestyle and environmental exposures. *BMC medicine*, *19*(1), 240. https://doi.org/10.1186/s12916-021-02097-z
Newbury, J [J.], Arseneault, L [L.], Caspi, A., Moffitt, T [T.], Odgers, C [C.], Belsky, D., Sugden, K., Williams, B., Ambler, A [A.], Matthews, T. & Fisher, H [H.] (2022). Association between genetic and socioenvironmental risk for schizophrenia during upbringing in a UK longitudinal cohort. *Psychological medicine*, *52*(8), 1527–1537. https://doi.org/10.1017/S0033291720003347
Nußbaum, R., Lucht, S., Jockwitz, C., Moebus, S., Engel, M., Jöckel, K.-H., Caspers, S. & Hoffmann, B. (2020). Associations of Air Pollution and Noise with Local Brain Structure in a Cohort of Older Adults. *ENVIRONMENTAL HEALTH PERSPECTIVES*, *128*(6), 67012. https://doi.org/10.1289/EHP5859.
O'Neill, E., Brereton, F., Shahumyan, H. & Clinch, J. P. (2016). The Impact of Perceived Flood Exposure on Flood-Risk Perception: The Role of Distance. *RISK ANALYSIS*, *36*(11), 2158–2186. https://doi.org/10.1111/risa.12597
Orru, H [Hans], Laukaitiene, A. & Zurlyté, I. (2012). Particulate air pollution and its impact on health in Vilnius and Kaunas. *MEDICINA-LITHUANIA*, *48*(9), 472–477. https://doi.org/10.3390/medicina48090070

Orru, H [Hans], Olstrup, H., Kukkonen, J., López-Aparicio, S., Segersson, D., Geels, C., Tamm, T., Riikonen, K., Maragkidou, A., Sigsgaard, T., Brandt, J., Grythe, H. & Forsberg, B [Bertil] (2022). Health impacts of PM2.5 originating from residential wood combustion in four nordic cities. *BMC PUBLIC HEALTH*, *22*(1), 1286. https://doi.org/10.1186/s12889-022-13622-x

Orru, K [K.], Tiittanen, P [P.], Ung-Lanki, S., Orru, H [H.] & Lanki, T [T.] (2022). Perception of Risks from Wood Combustion and Traffic Induced Air Pollution: Evidence from Northern Europe. *SUSTAINABILITY*, *14*(15). https://doi.org/10.3390/su14159660

Orru, K [Kati], Orru, H [Hans], Maasikmets, M., Hendrikson, R. & Ainsaar, M. (2016). Well-being and environmental quality: Does pollution affect life satisfaction? *QUALITY OF LIFE RESEARCH*, *25*(3), 699–705. https://doi.org/10.1007/s11136-015-1104-6

Oudin, A., Aström, D. O., Asplund, P., Steingrimsson, S., Szabo, Z. & Carlsen, H. K. (2018). The association between daily concentrations of air pollution and visits to a psychiatric emergency unit: a case-crossover study. *ENVIRONMENTAL HEALTH*, *17*(1), 4. https://doi.org/10.1186/s12940-017-0348-8

Oudin, A., Brabäck, L., Aström, D. O., Strömgren, M. & Forsberg, B [Bertil] (2016). Association between neighbourhood air pollution concentrations and dispensed medication for psychiatric disorders in a large longitudinal cohort of Swedish children and adolescents. *BMJ OPEN*, *6*(6), e010004. https://doi.org/10.1136/bmjopen-2015-010004

Oudin, A., Brabäck, L., Oudin Aström, D. & Forsberg, B [Bertil] (2017). Air Pollution and Dispensed Medications for Asthma, and Possible Effect Modifiers Related to Mental Health and Socio-Economy: A Longitudinal Cohort Study of Swedish Children and Adolescents. *International journal of environmental research and public health*, *14*(11). https://doi.org/10.3390/ijerph14111392

Oudin, A., Forsberg, B [Bertil], Lind, N., Nordin, S., Oudin Aström, D., Sundström, A. & Nordin, M. (2017). Is Long-term Exposure to Air Pollution Associated with Episodic Memory? A Longitudinal Study from Northern Sweden. *SCIENTIFIC REPORTS*, *7*(1), 12789. https://doi.org/10.1038/s41598-017-13048-1

Oudin, A., Frondelius, K., Haglund, N., Källén, K., Forsberg, B [Bertil], Gustafsson, P. & Malmqvist, E. (2019). Prenatal exposure to air pollution as a potential risk factor for autism and ADHD. *ENVIRONMENT INTERNATIONAL*, *133*, 105149. https://doi.org/10.1016/j.envint.2019.105149

Page, L., Hajat, S., Kovats, R. S. & Howard, L. (2012). Temperature-related deaths in people with psychosis, dementia and substance misuse. *The British journal of psychiatry : the journal of mental science*, *200*(6), 485–490. https://doi.org/10.1192/bjp.bp.111.100404

Papadatou, D., Giannopoulou, I., Bitsakou, P., Bellali, T., Talias, M. & Tselepi, K. (2012). Adolescents' reactions after a wildfire disaster in Greece. *Journal of traumatic stress*, *25*(1), 57–63. https://doi.org/10.1002/jts.21656

Peisker, J. (2023). Context matters: The drivers of environmental concern in European regions. *GLOBAL ENVIRONMENTAL CHANGE-HUMAN AND POLICY DIMENSIONS*, *79*. https://doi.org/10.1016/j.gloenvcha.2023.102636

Pelgrims, I., Devleesschauwer, B., Guyot, M., Keune, H., Nawrot, T., Remmen, R., Saenen, N., Trabelsi, S., Thomas, I [Isabelle], Aerts, R. & Clercq, E. de (2021). Association between urban environment and mental health in Brussels, Belgium. *BMC PUBLIC HEALTH*, *21*(1), 635. https://doi.org/10.1186/s12889-021-10557-7

Pereira, J., Vagos, P., Fonseca, A., Moreira, H., Canavarro, M. C. & Rijo, D. (2021). The Children's Revised Impact of Event Scale: Dimensionality and Measurement Invariance in a Sample of Children and Adolescents Exposed to Wildfires. *Journal of traumatic stress*, *34*(1), 35–45. https://doi.org/10.1002/jts.22634

Petrowski, K., Bastianon, C. D., Bührer, S. & Brähler, E. (2019). Air Quality and Chronic Stress: A Representative Study of Air Pollution (PM2.5, PM10) in Germany. *Journal of occupational and environmental medicine*, *61*(2), 144–147. https://doi.org/10.1097/JOM.0000000000001502

Petrowski, K., Bührer, S., Straus, B., Decker, O. & Brähler, E. (2021). Examining air pollution (PM10), mental health and well-being in a representative German sample. *SCIENTIFIC REPORTS*, *11*(1), 18436. https://doi.org/10.1038/s41598-021-93773-w

Pirkle, L., Jennings, N., Vercammen, A. & Lawrance, E. (2022). Current understanding of the impact of climate change on mental health within UK parliament. *FRONTIERS IN PUBLIC HEALTH*, *10*, 913857. https://doi.org/10.3389/fpubh.2022.913857

Pogacar, T., Znidarsic, Z., Kajfez Bogataj, L., Flouris, A., Poulianiti, K. & Crepinsek, Z. (2019). Heat Waves Occurrence and Outdoor Workers' Self-assessment of Heat Stress in Slovenia and Greece. *International journal of environmental research and public health*, *16*(4). https://doi.org/10.3390/ijerph16040597

Pogačar, T., Casanueva, A., Kozjek, K., Ciuha, U., Mekjavić, I. B., Kajfež Bogataj, L. & Črepinšek, Z. (2018). The effect of hot days on occupational heat stress in the manufacturing industry: implications for workers' well-being and productivity. *INTERNATIONAL JOURNAL OF BIOMETEOROLOGY*, *62*(7), 1251–1264. https://doi.org/10.1007/s00484-018-1530-6

Rahman, S., Zammit, S., Dalman, C. & Hollander, A.-C. (2022). Epidemiology of posttraumatic stress disorder: A prospective cohort study based on multiple nationwide Swedish registers of 4.6 million people. *European psychiatry : the journal of the Association of European Psychiatrists*, *65*(1), e60. https://doi.org/10.1192/j.eurpsy.2022.2311

Rakotozandry, T., Cassagne, E., Martin, S., Alauzet, P., Navarro, I., Delcroux, C., Caillaud, D., Besancenot, J.-P., Thibaudon, M. & Charpin, D. (2019). Exposure to Cypress Pollens and Subsequent Symptoms: A Panel Study. *International archives of allergy and immunology*, *180*(2), 135–141. https://doi.org/10.1159/000501223

Rathmann, J., Beck, C [C.], Flutura, S., Seiderer, A., Aslan, I. & André, E. (2020). Towards quantifying forest recreation: Exploring outdoor thermal physiology and human well-being along exemplary pathways in a

central European urban forest (Augsburg, SE-Germany). *URBAN FORESTRY & URBAN GREENING*, *49*. https://doi.org/10.1016/j.ufug.2020.126622

Razieh, C., Khunti, K., Davies, M., Edwardson, C., Henson, J., Darko, N., Comber, A., Jones, A. & Yates, T. (2019). Association of depression and anxiety with clinical, sociodemographic, lifestyle and environmental factors in South Asian and white European individuals at high risk of diabetes. *DIABETIC MEDICINE*, *36*(9), 1158–1167. https://doi.org/10.1111/dme.13986

Reuben, A., Arseneault, L [Louise], Beddows, A., Beevers, S., Moffitt, T [Terrie.], Ambler, A [Antony], Latham, R., Newbury, J [Joanne], Odgers, C [Candice], Schaefer, J. & Fisher, H [Helen] (2021). Association of Air Pollution Exposure in Childhood and Adolescence With Psychopathology at the Transition to Adulthood. *JAMA network open*, *4*(4), e217508. https://doi.org/10.1001/jamanetworkopen.2021.7508

Rittner, R., Flanagan, E., Oudin, A. & Malmqvist, E. (2020). Health Impacts from Ambient Particle Exposure in Southern Sweden. *International journal of environmental research and public health*, *17*(14). https://doi.org/10.3390/ijerph17145064

Ritz, B., Liew, Z., Yan, Q., Cui, a. X., Virk, J., Ketzel, M [M.] & Raaschou-Nielsen, O [O.] (2018). Air pollution and autism in Denmark. *ENVIRONMENTAL EPIDEMIOLOGY*, *2*(4), e028. https://doi.org/10.1097/EE9.0000000000000028

Rivas, I [Ioar], Basagaña, X [Xavier], Cirach, M [Marta], López-Vicente, M [Monica], Suades-González, E., Garcia-Esteban, R., Alvarez-Pedrerol, M [Mar], Dadvand, P. & Sunyer, J [Jordi] (2019). Association between Early Life Exposure to Air Pollution and Working Memory and Attention. *ENVIRONMENTAL HEALTH PERSPECTIVES*, *127*(5), 57002. https://doi.org/10.1289/EHP3169

Roberts, S., Arseneault, L [Louise], Barratt, B., Beevers, S., Danese, A., Odgers, C [Candice], Moffitt, T [Terrie], Reuben, A., Kelly, F. & Fisher, H [Helen] (2019). Exploration of NO2 and PM2.5 air pollution and mental health problems using high-resolution data in London-based children from a UK longitudinal cohort study. *Psychiatry research*, *272*, 8–17. https://doi.org/10.1016/j.psychres.2018.12.050

Robin, C., Beck, C., Armstrong, B [Ben], Waite, T. D [Thomas David], Rubin, G. J. & Oliver, I [Isabel] (2020). Impact of flooding on health-related quality of life in England: results from the National Study of Flooding and Health. *EUROPEAN JOURNAL OF PUBLIC HEALTH*, *30*(5), 942–948. https://doi.org/10.1093/eurpub/ckaa049

Rocha, K., Pérez, K., Rodríguez-Sanz, M., Obiols, J. & Borrell, C. (2012). Perception of environmental problems and common mental disorders (CMD). *Social psychiatry and psychiatric epidemiology*, *47*(10), 1675–1684. https://doi.org/10.1007/s00127-012-0474-0

Ronaldson, A., Arias de la Torre, Jorge, Ashworth, M., Hansell, A., Hotopf, M., Mudway, I., Stewart, R., Dregan, A. & Bakolis, I. (2022). Associations between air pollution and multimorbidity in the UK Biobank: A cross-sectional study. *FRONTIERS IN PUBLIC HEALTH*, *10*, 1035415. https://doi.org/10.3389/fpubh.2022.1035415

Russ, T., Cherrie, M., Dibben, C., Tomlinson, S., Reis, S., Dragosits, U., Vieno, M., Beck, R., Carnell, E., Shortt, N., Muniz-Terrera, G., Redmond, P., Taylor, A., Clemens, T., van Tongeren, M., Agius, R., Starr, J., Deary, I. & Pearce, J. (2021). Life Course Air Pollution Exposure and Cognitive Decline: Modelled Historical Air Pollution Data and the Lothian Birth Cohort 1936. *Journal of Alzheimer's disease : JAD*, *79*(3), 1063–1074. https://doi.org/10.3233/JAD-200910

Sauliene, I., Sukiene, L., Kainov, D. & Greiciuviene, J. (2016). The impact of pollen load on quality of life: a questionnaire-based study in Lithuania. *AEROBIOLOGIA*, *32*(2), 157–170. https://doi.org/10.1007/s10453-015-9387-1

Scibor, M. & Malinowska-Cieslik, M. (2020). The association of exposure to PM10 with the quality of life in adult asthma patients. *INTERNATIONAL JOURNAL OF OCCUPATIONAL MEDICINE AND ENVIRONMENTAL HEALTH*, *33*(3), 311–324. https://doi.org/10.13075/ijomeh.1896.01527

Shiue, I. (2015). Neighborhood epidemiological monitoring and adult mental health: European Quality of Life Survey, 2007-2012. *ENVIRONMENTAL SCIENCE AND POLLUTION RESEARCH*, *22*(8), 6095–6103. https://doi.org/10.1007/s11356-014-3818-3

Silverforsen, D., Theorell-Haglöw, J., Ljunggren, M., Middelveld, R [Roelinde], Wang, J., Franklin, K., Norbäck, D., Lundbäck, B., Forsberg, B [Bertil], Lindberg, E. & Janson, C [Christer] (2021). Snoring and environmental exposure: results from the Swedish GA2LEN study. *BMJ OPEN*, *11*(6), e044911. https://doi.org/10.1136/bmjopen-2020-044911

Sommar, J., Ek A., Middelveld, R [R.], Bjerg, A., Dahlén, S.-E., Janson, C [C.] & Forsberg, B [B.] (2014). Quality of life in relation to the traffic pollution indicators NO2 and NOx: Results from the Swedish GA2LEN survey. *BMJ OPEN RESPIRATORY RESEARCH*, *1*(1), e000039. https://doi.org/10.1136/bmjresp-2014-000039

Spence, A., Poortinga, W. & Pidgeon, N. (2012). The psychological distance of climate change. *RISK ANALYSIS*, *32*(6), 957–972. https://doi.org/10.1111/j.1539-6924.2011.01695.x

Sunyer, J [Jordi], Esnaola, M [Mikel], Alvarez-Pedrerol, M [Mar], Forns, J [Joan], Rivas, I [Ioar], López-Vicente, M [Mònica], Suades-González, E., Foraster, M., Garcia-Esteban, R., Basagaña, X [Xavier], Viana, M., Cirach, M [Marta], Moreno, T., Alastuey, A., Sebastian-Galles, N., Nieuwenhuijsen, M. & Querol, X [Xavier] (2015). Association between traffic-related air pollution in schools and cognitive development in primary school children: a prospective cohort study. *PLOS MEDICINE*, *12*(3), e1001792. https://doi.org/10.1371/journal.pmed.1001792

Tempest, E., Carter, B., Beck, C. & Rubin, G. J. (2017). Secondary stressors are associated with probable psychological morbidity after flooding: a cross-sectional analysis. *EUROPEAN JOURNAL OF PUBLIC HEALTH*, *27*(6), 1042–1047. https://doi.org/10.1093/eurpub/ckx182

Terpstra, T. (2011). Emotions, trust, and perceived risk: affective and cognitive routes to flood preparedness behavior. *RISK ANALYSIS*, *31*(10), 1658–1675. https://doi.org/10.1111/j.1539-6924.2011.01616.x

Tharrey, M., Sachs, A., Perignon, M., Simon, C., Mejean, C., Litt, J. & Darmon, N. (2020). Improving lifestyles sustainability through community gardening: results and lessons learnt from the JArDinS quasi-experimental study. *BMC PUBLIC HEALTH*, *20*(1), 1798. https://doi.org/10.1186/s12889-020-09836-6

Theleritis, C., Psarros, C., Mantonakis, L., Roukas, D., Papaioannou, A., Paparrigopoulos, T. & Bergiannaki, J. D. (2020). Coping and Its Relation to PTSD in Greek Firefighters. *The Journal of nervous and mental disease*, *208*(3), 252–259. https://doi.org/10.1097/NMD.0000000000001103

Thieken, A., Bessel, T., Kienzler, S., Kreibich, H., Müller, M [M.], Pisi, S. & Schröter, K. (2016). The flood of June 2013 in Germany: how much do we know about its impacts? *NATURAL HAZARDS AND EARTH SYSTEM SCIENCES*, *16*(6), 1519–1540. https://doi.org/10.5194/nhess-16-1519-2016

Thomas, I [I.], Martin, A., Wicker, A. & Benoit, L. (2022). Understanding youths' concerns about climate change: a binational qualitative study of ecological burden and resilience. *Child and Adolescent Psychiatry and Mental Health*, *16*(1), 110. https://doi.org/10.1186/s13034-022-00551-1

Tonne, C., Elbaz, A., Beevers, S. & Singh-Manoux, A. (2014). Traffic-related air pollution in relation to cognitive function in older adults. *EPIDEMIOLOGY*, *25*(5), 674–681. https://doi.org/10.1097/EDE.0000000000000144

Triebner, K., Markevych, I., Bertelsen, R., Sved Skottvoll, B., Hustad, S., Forsberg, B [Bertil], Franklin, K., Holm, M., Lindberg, E., Heinrich, J., Gómez Real, F. & Dadvand Payam (2022). Lifelong exposure to residential greenspace and the premenstrual syndrome: A population-based study of Northern European women. *ENVIRONMENT INTERNATIONAL*, *158*, 106975. https://doi.org/10.1016/j.envint.2021.106975

Triguero-Mas, M., Gidlow, C. J., Martinez, D., Bont, J. de, Carrasco-Turigas, G., Martinez-Iniguez, T., Hurst, G., Masterson, D., Donaire-Gonzalez, D., Seto, E., Jones, M. & Nieuwenhuijsen, M. (2017). The effect of randomised exposure to different types of natural outdoor environments compared to exposure to an urban environment on people with indications of psychological distress in Catalonia. *PLOS ONE*, *12*(3), e0172200. https://doi.org/10.1371/journal.pone.0172200

van den Berg, F., Verhagen, C. & Uitenbroek, D. (2015). The relation between self-reported worry and annoyance from air and road traffic. *International journal of environmental research and public health*, *12*(3), 2486–2500. https://doi.org/10.3390/ijerph120302486

Verheyen, V. J., Remy, S., Lambrechts, N., Govarts, E., Colles, A., Poelmans, L., Verachtert, E., Lefebvre, W., Monsieurs, P., Vanpoucke, C., Nielsen, F., van den Eeden, L., Jacquemyn, Y. & Schoeters, G. (2021). Residential exposure to air pollution and access to neighborhood greenspace in relation to hair cortisol concentrations during the second and third trimester of pregnancy. *ENVIRONMENTAL HEALTH*, *20*(1), 11. https://doi.org/10.1186/s12940-021-00697-z

Waite, T. D [Thomas David], Chaintarli, K., Beck, C. R., Bone, A [Angie], Amlot, R [Richard], Kovats, S., Reacher, M., Armstrong, B [Ben], Leonardi, G [Giovanni], Rubin, G. J. & Oliver, I [Isabel] (2017). The English national cohort study of flooding and health: cross-sectional analysis of mental health outcomes at year one. *BMC PUBLIC HEALTH*, *17*(1), 129. https://doi.org/10.1186/s12889-016-4000-2

Walker-Springett, K., Butler, C. & Adger, W. N. (2017). Wellbeing in the aftermath of floods. *HEALTH & PLACE*, *43*, 66–74. https://doi.org/10.1016/j.healthplace.2016.11.005

Ward Thompson, C., Curl, A., Aspinall, P., Alves, S. & Zuin, A. (2014). Do changes to the local street environment alter behaviour and quality of life of older adults? The 'DIY Streets' intervention. *British journal of sports medicine*, *48*(13), 1059–1065. https://doi.org/10.1136/bjsports-2012-091718

Weierstall-Pust, R., Schnell, T., Heßmann, P., Feld, M., Höfer, M., Plate, A. & Müller, M [Matthias] (2022). Stressors related to the Covid-19 pandemic, climate change, and the Ukraine crisis, and their impact on stress symptoms in Germany: analysis of cross-sectional survey data. *BMC PUBLIC HEALTH*, *22*(1), 2233. https://doi.org/10.1186/s12889-022-14682-9

Weinreich, G., Wessendorf, T., Pundt, N., Weinmayr, G., Hennig, F., Moebus, S., Möhlenkamp, S., Erbel, R., Jöckel, K.-H., Teschler, H. & Hoffmann, B. (2015). Association of short-term ozone and temperature with sleep disordered breathing. *EUROPEAN RESPIRATORY JOURNAL*, *46*(5), 1361–1369. https://doi.org/10.1183/13993003.02255-2014

Wind, T., Fordham, M. & Komproe, I. (2011). Social capital and post-disaster mental health. *GLOBAL HEALTH ACTION*, *4.* https://doi.org/10.3402/gha.v4i0.6351

Wind, T., Kawachi, I. & Komproe, I. (2021). Multilevel Social Mechanisms of Post-Disaster Depression. *International journal of environmental research and public health*, *18*(2). https://doi.org/10.3390/ijerph18020391

Zare Sakhvidi, M. J., Lafontaine, A., Lequy, E., Berr, C [Claudine], Hoogh, K. de [Kees], Vienneau, D [Danielle], Goldberg, M., Zins, M., Lemogne, C. & Jacquemin, B [Benedicte] (2022). Ambient air pollution exposure and depressive symptoms: Findings from the French CONSTANCES cohort. *ENVIRONMENT INTERNATIONAL*, *170*, 107622. https://doi.org/10.1016/j.envint.2022.107622

Zaremba, D., Kulesza, M., Herman, A., Marczak, M., Kossowski, B., Budziszewska, M., Michalowski, J., Klöckner, C., Marchewka, A. & Wierzba, M. (2022). A wise person plants a tree a day before the end of the world: coping with the emotional experience of climate change in Poland. *CURRENT PSYCHOLOGY.* Vorab-Onlinepublikation. https://doi.org/10.1007/s12144-022-03807-3

Zock, J.-P., Verheij, R., Helbich, M., Volker, B., Spreeuwenberg, P., Strak, M [Maciek], Janssen, N [Nicole], Dijst, M. & Groenewegen, P. (2018). The impact of social capital, land use, air pollution and noise on individual morbidity in Dutch neighbourhoods. *ENVIRONMENT INTERNATIONAL*, *121*, 453–460. https://doi.org/10.1016/j.envint.2018.09.008
